# Graph Learning in Bioinformatics: A Survey of Graph Neural Network Architectures, Biological Graph Construction and Bioinformatics Applications

**DOI:** 10.3390/biom16020333

**Published:** 2026-02-23

**Authors:** Lijia Deng, Ziyang Dong, Zhengling Yang, Bo Gong, Le Zhang

**Affiliations:** 1 Clinical Medical Research Center, Sichuan Academy of Medical Sciences and Sichuan Provincial People’s Hospital, School of Medicine, University of Electronic Science and Technology of China, Chengdu 610054, China; denglijia626@uestc.edu.cn; 2 School of Computer Science, Sichuan University, Chengdu 610065, China; 3 Genetic Diseases Key Laboratory of Sichuan Province and the Center for Medical Genetics, Department of Laboratory Medicine, Sichuan Academy of Medical Sciences and Sichuan Provincial People’s Hospital, School of Medicine, University of Electronic Science and Technology of China, Chengdu 610054, China; 4 Department of Human Disease Genes Key Laboratory of Sichuan Province and Institute of Laboratory Medicine, Sichuan Academy of Medical Sciences and Sichuan Provincial People’s Hospital, School of Medicine, University of Electronic Science and Technology of China, Chengdu 610054, China

**Keywords:** graph neural network, graph convolutional networks, bioinformatics, biological graph construction

## Abstract

Graph Neural Networks (GNNs) have become a central methodology for modelling biological systems where entities and their interactions form inherently non-Euclidean structures. From protein interaction networks and gene regulatory circuits to molecular graphs and multi-omics integration, the relational nature of biological data makes GNNs particularly well-suited for capturing complex dependencies that traditional deep learning methods fail to represent. Despite their rapid adoption, the effectiveness of GNNs in bioinformatics depends not only on model design but also on how biological graphs are constructed, parameterised and trained. In this review, we provide a structured framework for understanding and applying GNNs in bioinformatics, organised around three key dimensions: (1) graph construction and representation, including strategies for deriving biological networks from heterogeneous sources and selecting biologically meaningful node and edge features; (2) GNN architectures, covering spectral and spatial formulations, representative models such as Graph Convolutional Networks (GCNs), Graph Attention Networks (GATs), Graph Sample and AggregatE (GraphSAGE) and Graph Isomorphism Network (GIN), and recent advances including transformer-based and self-supervised paradigms; and (3) applications in biomedical domains, spanning disease–gene association prediction, drug discovery, protein structure and function analysis, multi-omics integration and biomedical knowledge graphs. We further examine training considerations, including optimisation techniques, regularisation strategies and challenges posed by data sparsity and noise in biological settings. By synthesising methodological foundations with domain-specific applications, this review clarifies how graph quality, architectural choice and training dynamics jointly influence model performance. We also highlight emerging challenges such as modelling temporal biological processes, improving interpretability, and enabling robust multimodal fusion that will shape the next generation of GNNs in computational biology.

## 1. Introduction

Biological systems are fundamentally organised as multi-scale, interdependent networks wherein genes, proteins, metabolites and phenotypes interact through highly structured yet heterogeneous relationships. These interactions are not arbitrary but shaped by biological principles such as molecular specificity, hierarchical regulation, pathway modularity, and spatial or temporal constraints. Advances in high-throughput sequencing, proteomics, chemical biology, and functional genomics have enabled the systematic characterisation of these interactions on an unprecedented scale, yielding diverse graph-based representations including protein–protein interaction networks, gene regulatory networks, molecular graphs, and multi-omics association graphs [1,2,3].

Biological systems pose a distinctive class of inference problems in which scientific questions are fundamentally relational rather than independent. Central tasks in bioinformatics–including disease–gene association discovery, molecular property prediction, drug–target interaction modelling, protein structure and function inference, pathway analysis, and multi-omics integration–require reasoning over complex networks of interacting biological entities. In these settings, biological meaning emerges not from isolated features but from patterns of connectivity, hierarchy, and context across genes, proteins, molecules, and phenotypes.

Crucially, the complexity of biomolecular systems manifests not only in the volume of data but also in the relational dependencies encoded within these graphs, encompassing non-Euclidean topologies, variable neighbourhood structures, and multi-scale connectivity. Addressing such tasks is essential for advancing disease modelling, therapeutic design, and precision medicine. However, traditional computational approaches often struggle to jointly capture long-range dependencies, multiscale organisation, and uncertainty inherent in biological systems, while also providing interpretable outputs that can support experimental validation and hypothesis-driven research [4,5].

Graph-based representations provide a natural abstraction for modelling these biological systems, as they explicitly encode molecular interactions, regulatory relationships, spatial proximity, and functional associations. Graph neural networks (GNNs) extend this representation by enabling end-to-end learning over biological graphs, allowing models to propagate, integrate, and transform information across interacting entities [6]. Importantly, GNNs are not merely predictive tools; when appropriately designed, they offer a framework for generating experimentally testable hypotheses by identifying influential nodes, critical interactions, and dysregulated network modules.

In biomolecular research, the value of graph neural networks extends beyond improved predictive performance to the level of molecular interpretation and mechanistic hypothesis generation. By operating directly on molecular graphs, interaction networks, and regulatory architectures, GNNs enable the systematic integration of structural, biochemical, and contextual information. In drug discovery, this includes learning structure–activity relationships, identifying functionally relevant substructures, and prioritising candidate compound–target interactions for downstream validation [7]. In protein science, GNN-based models can capture residue-level dependencies, domain organisation, and interaction interfaces that underpin structure–function relationships. In systems and regulatory biology, graph-based learning supports the identification of dysregulated pathways, key regulatory nodes, and multi-step signalling dependencies that are difficult to infer from feature-based models alone.

Importantly, these capabilities position GNNs as computational tools for narrowing experimental search spaces and generating biologically grounded hypotheses, rather than as substitutes for biochemical or cellular validation. When combined with domain knowledge and experimental evidence, graph-based models can help translate large-scale molecular data into testable insights about biomolecular function, interaction, and regulation.

For biological and clinical researchers, the appeal of GNNs lies in their ability to balance predictive accuracy with interpretability, to model long-range biological dependencies, and to scale to large, sparse, and heterogeneous datasets commonly encountered in genomics, proteomics, pharmacology, and systems biology. These properties position GNNs as a promising computational paradigm for bridging high-throughput data analysis with mechanistic biological understanding [3,8,9].

Despite the rapid growth of GNN applications in bioinformatics, existing surveys remain largely method-driven and may be difficult to navigate for biologists and physicians seeking to understand the practical benefits and limitations of these approaches [6,10,11,12]. Many reviews focus on cataloguing architectural variants or benchmarking performance, while providing limited guidance on how modelling choices relate to biological assumptions, experimental constraints, or interpretability requirements. As a result, critical challenges such as data noise, incompleteness, heterogeneity, uncertainty, and biological plausibility are often discussed in isolation rather than as central design considerations. Additionally, existing reviews seldom provide systematic coverage of biological datasets, leaving gaps in the documentation of data provenance, scale, modality, and evaluation protocols, which limits reproducibility and cross-study comparison. Furthermore, most prior works introduce GNN models without integrating biological constraints such as graph noise, sparsity, heterogeneity, interpretability requirements, and temporal dynamics. Methodologically, most reviews enumerate models without a unified framework linking Graph -> GNN -> GCN, limiting both depth and generalisation [13,14,15,16,17].

With the goal of making graph learning approaches more accessible and actionable for biological and clinical researchers, this review provides a widely, biologically grounded, and process-oriented synthesis of graph neural networks in bioinformatics. Our work differs from the existing literature through a multi-level integrative framework that organises the field across four conceptual layers: (1) biological graph construction grounded in domain principles; (2) methodological taxonomy spanning classical to modern GNN architectures; (3) systematised benchmarking through harmonised datasets and evaluation protocols; and (4) domain-centred applications highlighting both performance trends and biological constraints. This hierarchical organisation enables a coherent narrative from theoretical origins to application-level insights, unifying components that existing reviews treat separately. Our contributions are fourfold:

First, we establish a unified framework for understanding how biological data are transformed into graph representations, clarifying the principles, assumptions and limitations underlying PPI networks, gene regulatory graphs, molecular structures and multi-omics association graphs.

Second, we systematically organise GNN methodologies, ranging from spectral and spatial formulations to modern architectures, such as attention-based, structure-aware and transformer-integrated models, and contextualise their design choices in terms of biological relevance.

Third, we synthesise recent advances across major bioinformatics tasks, including disease–gene association prediction, drug discovery, protein structure and function modelling, multi-omics integration and multimodal biomedical analysis, providing a coherent comparison of application settings, datasets and performance trends.

Fourth, we analyse a broad collection of graph-based biological datasets, including molecular-level benchmarks, protein interaction resources, gene regulatory networks, drug–target interaction datasets and multimodal biomedical graphs. It offers an organised reference that supports reproducible evaluation and facilitates future GNN research in the life sciences. We further highlight overarching challenges such as graph construction quality, data noise, scalability, interpretability and temporal dynamics, offering perspectives on future methodological directions essential for enabling robust and biologically meaningful GNN models.

In summary, our main contributions are as follows:

- We synthesise recent GNN developments relevant to biological network modelling, spanning both classical and emerging architectures.
- We introduce a unified methodological framework that links graph construction, model design and training principles.
- We curate and systematise biological graph datasets across molecular, protein, regulatory, pharmacological and multimodal domains.
- We review major applications in bioinformatics and clinical analysis, covering molecular prediction, disease association, drug discovery and protein modelling.
- We identify key limitations and outline future research directions centred on graph quality, heterogeneity, scalability, interpretability and dynamic modelling.

## 2. Biological Networks and Data Representation

### 2.1. Overview of Biological Graph Structures in Bioinformatics: PPI, GRN, Molecular Graphs, Knowledge Graphs

A central premise of graph learning in bioinformatics is that many core biological questions are inherently relational. Genes do not act in isolation, proteins exert function through physical and regulatory interactions, and molecular phenotypes emerge from coordinated activity across multiple biological scales. Importantly, different biological tasks give rise to distinct graph structures, each reflecting specific biological assumptions and constraints.

Biological systems are inherently relational. Genes regulate each other, proteins assemble into complexes, cells communicate through ligand–receptor interactions, and tissues maintain structural organisation through spatial coordination. These biological dependencies naturally induce graph structures, in which nodes represent biological entities and edges encode functional, physical, or statistical relationships. Graph representations provide a principled way to formalise these dependencies by encoding biological entities as nodes and their relationships as edges. Importantly, different biological tasks give rise to distinct graph structures, each reflecting specific biological assumptions and constraints.

#### 2.1.1. Protein–Protein Interaction (PPI) 

Protein–protein interaction (PPI) networks model physical or functional interactions among proteins and serve as a foundational abstraction for studying cellular machinery, signalling pathways, and disease mechanisms. In PPI graphs, nodes represent proteins and edges denote experimentally measured or computationally inferred interactions. These networks implicitly assume that protein function is shaped by local neighbourhood context and network topology, an assumption supported by observations that disease-associated proteins often cluster within interaction modules [18]. PPI graphs are therefore well suited for tasks such as disease–gene association prediction, pathway enrichment analysis, and drug target identification, where relational proximity and network centrality carry biological meaning.

#### 2.1.2. Gene Regulatory Networks (GRNs) 

Gene regulatory networks (GRNs) encode directed and often hierarchical relationships between transcription factors, regulatory elements, and target genes. Unlike PPIs, GRNs reflect causal or directional influence, capturing how regulatory signals propagate across molecular layers. Nodes correspond to genes or regulatory factors, while edges represent activation or repression relationships inferred from transcriptomic, epigenomic, or perturbation data. GRNs embody biological assumptions of regulatory hierarchy, context dependence, and temporal control, making them particularly relevant for modelling cell fate decisions, developmental processes, and disease-associated dysregulation [19]. Their directed and dynamic nature poses additional challenges for graph learning, as models must account for asymmetry, feedback loops, and condition-specific rewiring.

#### 2.1.3. Molecular Graphs 

Molecular graphs represent individual chemical compounds or biomolecules at atomic or residue resolution, where nodes correspond to atoms (or amino acid residues) and edges encode covalent bonds or spatial proximity. These graphs are grounded in physicochemical principles, assuming that molecular properties and biological activity arise from local chemical environments and structural configuration. Molecular graphs underpin tasks such as quantum property prediction, drug-like molecule screening, and drug–target interaction modelling [7]. In this setting, graph representations must preserve chemical validity, stereochemistry, and local geometric constraints, highlighting the importance of biologically and physically informed graph construction.

#### 2.1.4. Knowledge Graphs and Heterogeneous Biological Graphs

Knowledge graphs and heterogeneous biological graphs, beyond single-modality networks, integrate multiple entity types into a unified relational framework, such as genes, proteins, diseases, drugs, phenotypes, and pathways. These graphs capture cross-scale and cross-domain relationships derived from curated databases, literature mining, and experimental evidence. By explicitly modelling heterogeneous node and edge semantics, knowledge graphs support integrative tasks including drug repurposing, disease mechanism elucidation, and multi-omics association analysis. Their structure reflects the assumption that biological insight emerges from the interaction of diverse evidence sources rather than any single data modality [20,21].

Across these graph types, a common challenge lies in balancing biological fidelity with computational tractability. Biological graphs are often incomplete, noisy, and context dependent, reflecting limitations of experimental measurement and biological variability. Consequently, graph construction is not a neutral preprocessing step but a biologically informed modelling decision that strongly influences downstream learning and interpretation.

In general, nodes represent phenomena spanning multiple biological scales. At the molecular level, nodes may correspond to genes, proteins, miRNAs, or metabolites, each characterised by expression profiles, sequence features, or structural descriptors. Edges describe the relationships governing biological systems, reflecting diverse forms of biological knowledge, including physical interaction edges, regulatory edges, metabolic edges, spatial edges, and statistical relationships. The construction of such graphs plays a central and scientifically pivotal role in biosystem formation. The choice of how nodes and edges are defined determines the flow of biological logical information within GNNs, directly shaping the model’s interpretability, generalisability, and capacity to reflect underlying biological mechanisms. Understanding the assumptions embedded in different graph structures is therefore essential for selecting appropriate graph neural network architectures and for interpreting model outputs in a biologically meaningful way [20].

### 2.2. Data Characteristics and Challenges (Noise, Incompleteness, Heterogeneity)

Biological data are inherently relational. Genes regulate each other through transcriptional programmes, proteins interact to form functional complexes, and small molecules bind to targets to modulate physiological processes. These interactions naturally form networks whose topology encodes mechanistic and functional dependencies within living systems [20]. As a result, the construction and curation of biological graph datasets have become a central component in computational biology, particularly as graph neural networks (GNNs) increasingly serve as powerful tools for analysing such structured information.

Unlike generic graph datasets used in computer science, biological graph datasets are derived from experimental measurements, curated biomedical knowledge, or multi-omics integration pipelines. They are often noisy, incomplete, heterogeneous across data sources, and shaped by biological constraints such as molecular structure, evolutionary conservation, or physical interaction interfaces [22]. These characteristics introduce unique modelling challenges but also provide rich opportunities for extracting biologically meaningful patterns [19].

The rapid adoption of GNNs in bioinformatics has accelerated the development of diverse biological graph datasets—ranging from protein–protein interaction networks and drug–target interaction databases to molecular graphs, gene regulatory networks, knowledge graphs, and multimodal association graphs derived from genomics, transcriptomics, and clinical data. Systematic investigation of these datasets is essential not only for benchmarking GNN algorithms but also for understanding how graph construction choices, annotation quality, and biological context affect downstream model performance.

Therefore, summarising representative biological graph datasets provides a foundation for evaluating existing GNN-based approaches and guiding future research on more biologically informed, robust, and interpretable graph learning models in the life sciences.

In this section, we will briefly introduce some existing datasets related to graph neural networks. To avoid conceptual ambiguity and to improve clarity regarding data provenance and reproducibility, we explicitly distinguish three complementary levels of biological data resources used in graph learning research, which are summarised in Table 1, Table 2 and Table 3.

Table 1 presents representative benchmark datasets that are commonly used for training and evaluating graph neural networks in bioinformatics. These datasets correspond to fixed, task-defined collections of graphs (e.g., molecular property prediction benchmarks, protein classification datasets, or disease association datasets) with clearly specified learning objectives and labels. They serve as standardised evaluation benchmarks and enable controlled comparison of GNN architectures across studies.

Table 2 summarises major biological databases and knowledgebases that provide the raw experimental evidence or curated biological knowledge from which many benchmark datasets are derived. Resources such as ChEMBL, STRING, and KEGG are continuously updated and do not constitute datasets in a strict sense; instead, they function as foundational data sources supplying molecular interactions, pathways, and annotations that are subsequently filtered, sampled, or integrated to construct task-specific graph datasets.

Table 3 reports dataset statistics commonly cited in graph learning benchmarks, including the number of graphs, nodes, edges, feature dimensions, and class labels. For datasets originating from continuously evolving databases (e.g., ChEMBL or ZINC), the reported statistics correspond to representative subsets or snapshots adopted in prior studies, rather than the complete underlying resource. This table is included to facilitate methodological comparison and computational cost assessment across GNN models, while preserving transparency regarding the nature of the underlying data sources.

#### 2.2.1. Quantum/Property Prediction

##### QM9

Ramakrishnan et al. [23] assembled a dataset intending to offer quantum chemical attributes across a pertinent, coherent, and extensive range of minor organic molecules. This dataset encompasses computations encompassing geometric, energetic, electronic, and thermodynamic characteristics of stable minor organic molecules, totalling 134,000 entries. These molecules constitute a subset of the complete 133,885 distinct species possessing up to 9 heavy atoms (CONFs) within the expansive GDB-17 chemical domain, consisting of a staggering 166 billion organic molecules. This repository serves as a valuable resource for the assessment and validation of current methodologies, as well as the formulation of novel approaches like hybrid quantum mechanics/machine learning, and the elucidation of relationships between molecular structure and properties.

##### ZINC

Irwin and Shoichet [24] collected ZINC database is a curated collection of commercially available compounds designed to support large-scale virtual screening and molecular property prediction. It provides three-dimensional structures, molecular descriptors, and bioactive-relevant chemical scaffolds for millions of drug-like molecules, and in total 12,000 graphs. The subset commonly used in graph learning research contains carefully filtered small molecules with balanced physicochemical characteristics, ensuring their relevance for pharmaceutical discovery. Due to its breadth and diversity, ZINC serves as an essential benchmark for evaluating molecular representation learning methods, enabling the development of models that generalise across heterogeneous chemical spaces and support downstream applications such as de novo drug design, lead optimisation, and quantitative structure–activity relationship analysis.

#### 2.2.2. Drug-like Molecule Collections

##### D&D

Dobson and Doig [25] constructed a dataset containing 1178 protein structures and their corresponding secondary structure content, amino acid obligations, surface properties, and ligands. This dataset is used to predict protein structure or analyse protein function.

##### PROTEIN

Borgwardt et al. [26] collected a dataset for protein function prediction. This dataset contains information on the sequence, structure, chemical properties, amino acid motifs, and interaction partners or physiological profiles of proteins. Researchers can use protein information to predict functional class membership of enzymes and non-enzymes. This dataset contains 1113 samples.

##### ChEMBL

Gaulton et al. [27] collected the ChEMBL database, which is a manually curated repository of bioactive molecules with experimentally measured interactions against a diverse array of biological targets. It integrates chemical structures, binding affinities, pharmacokinetic metadata, and assay information derived from the medicinal chemistry literature. The dataset encompasses millions of compound–target associations, covering enzymes, GPCRs, ion channels, and other therapeutically relevant proteins. Owing to its scale and biological diversity, ChEMBL provides a rich foundation for training graph-based models in drug–target interaction prediction, mechanism-of-action inference, polypharmacology analysis, and multi-target drug discovery. Its comprehensive coverage positions it as a cornerstone dataset for method development in computational drug design. This dataset contains 5200 protein targets.

##### MoleculeNet

Wu et al. [28] introduced MoleculeNet, which is an extensive benchmark suite introduced to standardise the evaluation of machine learning models in molecular science. It aggregates a broad spectrum of datasets derived from quantum chemistry, physical chemistry, biophysics, and physiology, including Tox21, HIV, BACE, and FreeSolv. These datasets encompass tasks ranging from toxicity prediction and biochemical binding affinity estimation to solubility and hydration energy regression. MoleculeNet further provides split protocols, evaluation metrics, and consistent preprocessing pipelines, thereby enabling rigorous and reproducible comparisons across modelling approaches. As such, it has become a foundational resource for assessing the capability of graph neural networks to capture complex chemical semantics and molecular structure–property relationships.

#### 2.2.3. Systems Biology Datasets

##### PPI

Greene et al. [29] utilised low-throughput tissue-specific gene expression data to map genes onto the Human Protein Reference Database (HPRD) tissues. This gene-to-tissue mapping was then integrated with the human protein–protein interaction (PPI) network. The outcome was a multi-layer tissue network comprising 107 layers, each representing a tissue-specific PPI network. The initial human PPI network was curated from diverse sources, encompassing studies by Orchard et al. (2013) [30], Rolland et al. (2014) [31], Chatr-Aryamontri et al. (2014) [32], Prasad et al. (2008) [33], Ruepp et al. (2009) [34], and Menche et al. (2015) [35]. This collection specifically focused on physical PPIs that were substantiated by experimental evidence, excluding interactions based on gene expression and evolutionary data. The comprehensive, unweighted human PPI network consisted of 21,557 interconnected proteins, forming 342,353 interactions. For each of the 107 unique tissues, a tissue-specific PPI network was generated from the overarching PPI network. During this process, every link within the overarching PPI network was identified as specifically co-expressed within that particular tissue. This categorisation followed the criteria outlined by Greene, et al. [29], where interactions were labelled as specifically co-expressed if both associated proteins were tissue-exclusive or if one was tissue-specific and the other broadly expressed. The list of specifically co-expressed proteins was extracted from Greene, et al. [29]. Ultimately, a specific tissue’s PPI network constitutes a subset of the overall PPI network, formed by the collection of edges that are specifically co-expressed within that tissue.

#### 2.2.4. Bio-Chemical Graph Classification Benchmarks

##### NCI-I

Wale, et al. [36] extended the dataset, which is obtained from the National Cancer Institute’s DTP AIDS Antiviral Screen programme, based on vectors to enable classification and sorting retrieval in the process of studying the underlying molecular topology of drug compounds. The dataset has 4110 graphs and 37 features.

##### PTC

Toivonen, et al. [37] collected this dataset for studying the relationship between chemical molecular structure and carcinogenicity. The dataset contains 2694 samples, including 1954 for training and 740 for testing.

##### MUTAG

Debnath, et al. [38] collected the MUTAG dataset, which consists of nitroaromatic compounds labelled according to their mutagenic effects on the bacterium *Salmonella typhimurium*, which includes 7831 graphs. Each molecule is encoded as a graph with atoms as nodes and chemical bonds as edges, supplemented with functional group information known to influence mutagenicity. As one of the earliest benchmarks in chemical graph analysis, MUTAG offers a compact yet biologically meaningful classification task. Its well-characterised structure–activity relationships make it suitable for assessing the ability of graph neural networks to capture molecular substructures associated with toxicological outcomes.

### 2.3. Common Graph Construction Strategies in Bioinformatics

Constructing biologically meaningful graphs is a foundational step for applying graph neural networks (GNNs) to bioinformatics tasks. Biological datasets are inherently heterogeneous, noisy, and often incomplete, requiring graph construction strategies that balance biological plausibility with computational tractability. This section summarises common principles and methodological categories for constructing graphs from biological data, and highlights their relevance across molecular biology, systems biology, and biomedical informatics.

When applying graph neural networks to biomedical information systems (such as gene regulatory networks, single-cell atlases, and drug–target interaction graphs), raw data typically exists in high-dimensional, sparse, and heterogeneous forms (e.g., gene expression matrices, protein sequences, and electronic health record event streams). Consequently, the core task of data processing is to transform raw biomedical data into graph representations that are structurally coherent, noise-controlled, and information-rich.

It should be noted that graph structure optimisation can be categorised into two types: one involves learning a fixed optimised graph (such as GSL or curvature reconnection) prior to or during training, constituting data preprocessing; the other entails randomly perturbing the graph structure during each training iteration (such as DropEdge), representing a training regularisation strategy, which will be detailed in Section 2.3.

Graph construction involves mapping raw biological entities and their relationships onto nodes and edges. In biomedical contexts, common construction strategies include:

Co-occurrence/similarity-based: For instance, in single-cell RNA-seq analysis, cells serve as nodes, with edges constructed by calculating inter-cellular similarity via k-nearest neighbours (kNN) or Gaussian kernels; in disease–gene–gene association studies, nodes represent ICD codes or Gene Ontology (GO) terms, with co-occurrence frequency serving as edge weight.

Prior knowledge-based: For instance, utilising the STRING database [39] to construct protein–protein interaction (PPI) networks, or building metabolic reaction graphs based on KEGG pathways [40].

Multimodal fusion graphs, e.g., unifying heterogeneous entities such as genes, cells, and drugs into a heterogeneous graph, defining cross-type relationships via meta-paths.

High-quality graph construction directly impacts downstream task performance, necessitating a balance between biological plausibility and computational scalability.

In biomedical contexts, graph construction involves mapping biological entities and their relationships onto nodes and edges. These relationships are derived from experimental measurements, prior knowledge, or statistical associations. Common construction strategies include:

(1) Graph Structure Learning (GSL): Treating the adjacency matrix as learnable parameters, dynamically adjusting edge weights or topology via end-to-end optimisation. For instance, combining node embedding similarity with the original structure to generate an optimised adjacency matrix through attention or interpolation mechanisms.

(2) Curvature Rewiring: Uses Jost–Liu curvature [41], a graph-based analogue of Ricci curvature that measures how easily information can be transported across an edge, to distinguish bottleneck edges connecting different network modules from redundant edges within densely connected regions. Edges with negative curvature typically indicate structural bridges, whereas positive curvature reflects local redundancy. Curvature-guided edge addition or removal helps regulate message propagation and alleviates over-smoothing and over-squeezing in biological graphs.

(3) Effective Resistance Optimisation: By minimising the graph’s total effective resistance, this approach holistically regulates edge deletion (reducing redundancy) and edge insertion (alleviating sparsity), thereby enhancing connectivity and information propagation efficiency.

(4) Edge Removal Sampling: By probabilistically or strategically removing edges—particularly those connecting nodes of different classes or with low task-specific importance—this strategy sparsifies the graph to slow down over-convergence of node representations, thereby improving computational efficiency and mitigating over-smoothing while preserving essential topological properties.

**Figure 1:**
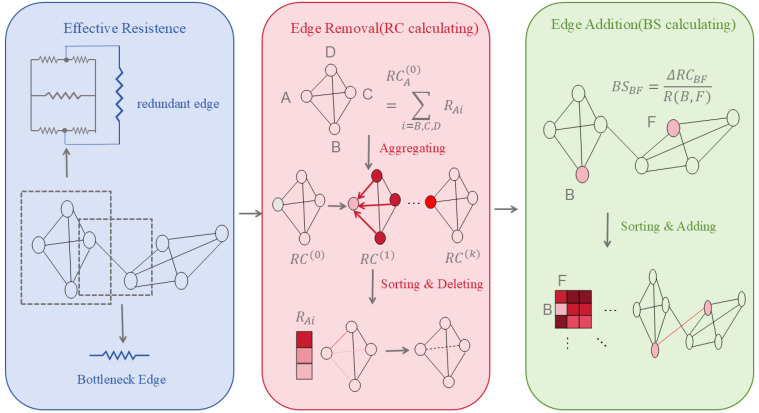
The Evolution of Effective Resistance Optimisation.

These methods prove particularly suited to biomedical scenarios characterised by sparse annotations and high structural noise (e.g., novel target discovery, rare disease network modelling). The changes are shown in Figure 1.

#### 2.3.1. Graph Structure Learning (GSL)

The most common graph optimisation technique is GSL, which treats graph data as the learnable entity itself. While using GCN to learn node representations, it dynamically learns/modifies the graph’s adjacency structure (adding edges, removing edges, adjusting weights). Since it dynamically adjusts the graph structure based on the actual problem and model, GSL can optimise graph neural networks in various aspects (such as preventing overfitting and over-smoothing) and significantly enhance model robustness against adversarial attacks [42,43,44,45].

Specifically, GSL’s structural modelling involves modifying the original graph’s adjacency matrix:

(1) $$A^{*}=g\left(A,{\overset{\sim }{A}}_{\theta }\left(X,Z\right)\right)$$

${\overset{\sim }{A}}_{\theta }\left(X,Z\right)$: Candidate adjacency obtained through a structural modelling function (dependent on node features X, embeddings Z, or learnable parameters θ), $g\left(\cdot ,\cdot \right)$: Update function (e.g., interpolation, attention fusion, direct replacement).

#### 2.3.2. Curvature Rewiring

However, in recent years, as graph neural network architectures have grown increasingly complex, over-smoothing and over-squashing have emerged as two opposing challenges. Over-smoothing occurs when node embeddings gradually converge as the number of convolutional layers increases, blending representations from different clusters and causing each node to lose its distinctive features. Over-squashing, on the other hand, refers to information compression issues during long-range dependencies: Features from distant nodes must propagate through a finite number of layers, forcing an exponential amount of neighbour information into a fixed-dimensional vector. This causes the contribution of long-range dependencies to sharply decay (especially in “bottleneck” scenarios with few connections between nodes). These two issues often come at the expense of each other—mitigating one typically exacerbates the other. To address this, researchers have introduced curvature reconnection methods.

Specifically, we have a definition to quantitatively describe the training characteristics: Let S⊂V be a node set, and let ∂S denote the set of edges crossing between S and V/S, i.e., ∂S=[(u,v)∈E:u∈S,v∈V⧵S]. The Cheeger constant of graph G is defined as [46]:

(2) $$h_{G}={min}_{S}\frac{\left|\partial S\right|}{min\left(vol\left(S\right),vol\left(V/S\right)\right)},$$

where $vol(S)=\sum_{i\in S}d_{i}$.

Intuitive interpretation: If there exists a bottleneck in the graph, then $h_{G}$ is small. It is known that for a connected graph, $h_{G}>0$, Furthermore, $\lambda_{2}$ is related to $h_{G}$ by the Cheeger inequality:

(3) $$2h_{G}\geq \lambda_{2}\geq \frac{h_{G}^{2}}{2}.$$

If there exists a partition S, where both sides have large volumes but few connecting edges, then this ratio will be small, which indicates a significant bottleneck in the graph. So, the $h_{G}$ will be larger and $\lambda_{2}$ will be larger.

The Stochastic Jost–Liu curvature-based Rewiring (SJLR) method can be defined as follows: 

Curvature Calculation: For each edge (i,j), compute the Jost–Liu curvature:

(4) $$JLC(i,j)=\frac{2}{d_{i}}+\frac{2}{d_{j}}-2+2\frac{\#(i,j)}{d_{i}\vee d_{j}}+\frac{(\#(i,j)-(d_{i}-1){)}_{+}}{d_{i}(d_{i}-1)}+\frac{(\#(i,j)-(d_{j}-1){)}_{+}}{d_{j}(d_{j}-1)}$$

Here, #(i,j) is the number of triangles containing the edge (i,j), $d_{i}$ and $d_{j}$ are the degrees of the nodes, and $(\cdot {)}_{+}$ indicates taking the positive value. The smaller the JLC, the more likely it is a bottleneck edge (more likely to be removed).

#### 2.3.3. Edge Removal Sampling

For existing edge E:

(5) $$P_{\mathrm{drop}}\left(i,j\right)=softmax(\alpha \phi_{d}+(1-\alpha )d_{n}^{\left(l\right)})$$

Specifically, $\phi_{d}$ is the result of concatenating the JLC values of each edge, followed by a normalisation process, and $d_{n}^{\left(l\right)}$ is the normalised node embedding distance (representing local information disparity).

Edge Addition Sampling

For candidate non-edges (u, v), we define σ(m) as the improvement score resulting from the addition of edge (r,s). A higher σ(m) value signifies that incorporating this edge leads to a substantial increase in local curvature, thereby mitigating bottleneck structures within the graph:

(6) $$\sigma (m)=\frac{1}{\left|E_{\left(r,s\right)}\right|}\sum_{(i^{\prime },j^{\prime })\in E_{\left(r,s\right)}}\left({JLC}^{\prime }(i^{\prime },j^{\prime })-JLC(i^{\prime },j^{\prime })\right)$$

After concatenating and normalising σ(m) we obtain $\phi_{a}$, we also introduced $a_{n}^{\left(l\right)}$, which represents the distance between the embeddings of the nodes connected by a candidate edge.

(7) $$P_{add}=\left(i,j\right)=\mathrm{softmax}(\alpha \phi_{a}-(1-\alpha )a_{n}^{\left(l\right)})$$

#### 2.3.4. Effective Resistance Optimisation

In 2024, Xu Shen et al. [47] introduced the concept of effective resistance to unify these two problems into a single framework: minimising the total constrained effective resistance. This is achieved by manipulating edge deletion and insertion, defined as:

(8) $${min}_{E}\sum_{\left(i,j\right)\in E}R_{G}\left(i,j\right)s.t.\;R_{G}\left(i,j\right)\geq \delta ,$$

where the resistance matrix is computed using the Laplacian pseudoinverse:

(9) $$R_{G}(u,v)=(\delta (u)-\delta (v){)}^{†}L^{†}(\delta (u)-\delta (v))$$

Thus, the condition for edge deletion is maximising the topological redundancy coefficient, calculated as follows:

(10) $$RC_{i}^{\left(k\right)}=\left(1-\alpha \right)\sum_{j\in N\left(i\right)}w_{ij}RC_{j}^{\left(k-1\right)}+\alpha RC_{i}^{\left(0\right)},for\;node\;i\in V,$$

The condition for edge insertion is minimising the bottleneck sparsity coefficient, calculated as follows:

(11) $$BS_{ij}^{\left(k\right)}=\frac{\left|RC_{i}-RC_{j}\right|}{R_{G}\left(i,j\right)},\;for\;node\;i\in V\;and\;j\in N\left(v\right),v$$

For the n nodes with the largest RC, delete one of their smallest adjacent edges; for the n candidate edges with the smallest BS, insert one new edge with the largest value in their respective neighbourhoods. Thus, by removing edges and adding edges, the issues of excessive smoothing and excessive compression can be mitigated.

## 3. Graph Neural Networks for Biological Data: Foundations and Key Architectures

The construction of biologically meaningful graphs defines the foundation upon which graph neural networks operate. Once biological entities and their relationships are encoded into graphs through molecular structures, interaction networks, or multi-omics integration, the next challenge is selecting computational frameworks that can learn from these complex, irregular topologies. Unlike traditional machine learning methods that assume grid-like or sequential structures, GNNs are explicitly designed to model relational dependencies embedded within biological systems. Understanding their theoretical underpinnings and architectural variations is therefore essential for bridging the transition from biological graph construction to effective downstream analysis. In this chapter, we introduce the core formulations and key model families that enable learning on non-Euclidean biological data. Additionally, this chapter emphasises the biological assumptions, applicability, and limitations of major GNN design paradigms, highlighting when specific modelling choices are appropriate or inappropriate for different classes of biomolecular graphs.

### 3.1. Spectral and Spatial Formulations

In the realm of graph theory, any dataset can establish topological relationships within a normed space [5]. Topological connectivity serves as a versatile data structure, offering the potential to harness this data for diverse tasks spanning various domains, including computer vision and natural language processing, despite differences in data categorisation [5,48,49]. However, a significant challenge arises when attempting to apply traditional Convolutional Neural Network (CNN) architectures to data with non-Euclidean structures. This challenge stems from the fact that in a topological graph, the number of adjacent vertices for each vertex may vary, rendering the utilisation of a fixed-size convolution kernel for convolutional processing impractical. In particular, relational data in the real world, such as protein interactions and patient–disease associations, naturally manifest as non-Euclidean graph structures. The number of neighbours for each node is not fixed, precluding the direct application of fixed-size convolutional kernels.

To overcome this limitation, researchers have innovatively introduced Graph Convolutional Networks (GCNs). These GCNs are specifically designed to extract spatial features from topological maps, enabling the effective processing of data characterised by variable connectivity patterns. This ‘message passing’ mechanism aligns with intuition: the semantic meaning of a node should be influenced by its local topological environment.

In the context of utilising Convolutional Neural Networks (CNNs) to extract spatial features from a topological map, a fundamental requirement is to enable the convolutional layer to compute the topological map. This entails the projection of the topological map from a non-Euclidean space to a Euclidean space.

As the property of a real symmetric positive semi-definite, the symmetrically normalised Laplacian matrix can be factored as $L=U\Lambda U^{T}$, where $U=\left[u_{0},u_{1},\ldots ,u_{n-1}\right]\in R^{n\times n}$ is a matrix consisting of eigenvectors $u_{0}$ of the Laplacian matrix where $u_{n-1}$ is also the well-known Fourier functions, and Λ is a diagonal matrix of eigenvalues $\lambda_{i}$ of the Laplacian matrix where $\lambda_{i}$ is also called the spectrum of a graph. These Fourier functions form an orthonormal basis; thus, $U\Lambda U^{T}=I$, $\left\langle u_{k},u_{k^{\prime }}\right\rangle =\delta_{kk^{\prime }}$.

The lead-in to graph convolution is the Fourier transformation defined by graph signal processing academics for use with graph data. A graph signal $x\in R^{n}$ in graph signal processing is a vector consisting of the features of all nodes in the graph, where $x_{i}$ is the feature of the ith node. The Fourier series can be calculated by decomposing the graph signal x with Fourier functions [50]:

(12) $$D=\left\{\begin{array}{c} \left(U_{1},v_{1}\right),\;\left(U_{2},v_{2}\right),\;\ldots ,\;\left(U_{n},v_{n}\right)\} \end{array}\right.$$

(13) $$x=\sum_{k=0}^{n-1}\left\langle u_{k},x\right\rangle u_{k}=UU^{T}x=U\hat{x}$$

Thus, the graph Fourier transform to a graph signal x can be defined as

(14) $$F\left(x\right)=U^{T}x=\hat{x}.$$

Since each column vector of U and each row vector of $U^{T}$ is an eigenvector of the graph Laplacian, and the property of mutual orthogonality of the graph Laplacian eigenvectors [51], the product of $U^{T}$ and x realises the orthogonal projection of the graph signal x and the orthogonal basis is formed by the eigenvectors of the graph Laplacian. In addition, the inverse graph Fourier transform can be defined as

(15) $$F^{\;-1}\left(\hat{x}\right)=U\hat{x}=U^{T}Ux=x,$$

where the $\hat{x}$ is the vector consists of the coordinates of the graph signal x in the frequency domain.

According to the convolution theorem, which states that the Fourier transform of the convolution of two functions is the product of their individual Fourier transforms, the convolution result of a graph signal x and a convolution kernel h can be obtained by applying the inverse Fourier transform to the product of their individual Fourier transforms:

(16) $${\left(x*h\right)}_{G}=\;U\left(\left(U^{T}h\right)⊙\left(U^{T}x\right)\right),$$

Building upon this foundation, investigators project the graph’s structure from a non-Euclidean space onto a Euclidean space, subsequently conducting convolution operations on the data within this projected space. The outcomes of these convolutions are subsequently reconstructed in the original space. This methodology facilitates the employment of Convolutional Neural Networks (CNNs) for the learning and prediction of relational graphs. This conceptualisation directly paved the way for the emergence of Spectral Graph Convolutional Networks (Spectral GCNs).

Spectral GCNs are a class of graph convolutional networks that primarily leverage the spectral domain to perform convolution operations on graph-structured data. These networks are built upon the theoretical foundation of graph signal processing, which involves using the eigenvalues and eigenvectors of a graph’s Laplacian matrix to analyse and process graph data.

Spectral GCNs use a convolution algorithm to define graph convolution from the spectral domain. As noted above, for the vector x, which is constituted by the feature extraction of the graph nodes, the basic formula of the convolutional output $y_{output}$ of a GCN is as follows:

(17) $$y_{output}=\sigma \left(UhU^{T}x\right),$$

where the σ() is the activation function.

In Spectral GCNs, convolution operations are performed by transforming the graph’s data into the spectral domain, applying spectral filters to this transformed data, and then converting it back to the spatial domain. This process allows the network to capture both local and global graph structures and dependencies. Spectral GCNs have shown promise in various applications, including node classification, link prediction, and graph classification. However, they also have limitations, such as scalability issues when dealing with large graphs and difficulties in handling dynamic graphs.

The approaches delineated above predominantly initiate graph convolution within the spectral domain, commencing from the convolution algorithm as a foundational basis. Conversely, the spatial approach embarks from the node domain, thereby individuating each central node and its associated neighbours via the specification of an aggregation function. Notably, the Chebyshev network and first-order Graph Convolutional Network can be likened to Laplacian matrices, where their variables play an analogous role akin to aggregation functions. In light of this, recent research endeavours have drawn inspiration from this perspective, endeavouring to directly acquire knowledge regarding aggregation functions from the node domains, employing mechanisms such as attention mechanisms [52] and Recurrent Neural Networks (RNNs) [53]. Furthermore, Danel [54] and Qin [55] have introduced a comprehensive framework for GCNs from a spatial vantage point, elucidating the internal intricacies of spatial GCNs. The spatial convolution-based method directly prescribes convolutional operations for the connected relationships of each node, resembling conventional convolution within traditional Convolutional Neural Networks. This universal framework’s formulation illuminates the core considerations in GCN and provides a foundation for the comparative analysis of existing research efforts.

#### Comparison Between Spectral and Spatial GCNs

In recent years, spatial models have gained increasing prominence, serving as complementary theoretical foundations alongside spectral models in the realm of graph processing within graph convolutional networks. It is noteworthy that spatial models offer several advantages that are challenging for spectral models to match.

Firstly, spatial models excel in terms of efficiency, particularly when dealing with expansive graphs. As graph size escalates, the computational complexity of spectral models undergoes a steep rise, posing significant hurdles in handling a diverse spectrum of graph structures. Spectral models typically necessitate the computation of feature vectors or the processing of the entire graph, thereby leading to scalability concerns. Conversely, spatial models have the capacity to operate on selected nodes rather than the entire graph, harnessing node sampling techniques to enhance efficiency.

Secondly, relative to spectral models, spatial models confer heightened flexibility. Spectral models are primarily tailored for undirected graphs, as Laplace matrices lack a definition for directed graphs. When spectral models are applied to directed graphs, they frequently convert them into undirected ones, thereby constraining their applicability. In contrast, spatial models demonstrate versatility by accommodating any graph structure, whether directed or undirected [56].

Furthermore, with regard to model universality, spectral models embrace a fixed graph structure, rendering them less adaptable to the addition of new nodes or the ever-evolving dynamics of networks [57]. Conversely, spatial models effectively facilitate weight sharing across diverse positions and structures. These models facilitate localised convolution at each node, enabling them to adapt to dynamically changing graph configurations and seamlessly integrate new nodes.

In conclusion, as depicted in Table 4, spatial GCNs have showcased notable advantages in computational efficiency, flexibility, and adaptability, positioning them as a promising avenue for advancing graph convolution technology.

Despite the remarkable success of GCNs, they still face three fundamental challenges that directly drive subsequent innovations in data processing, architectural design, and training strategies:

Over-smoothing: Node representations in deep networks tend to converge, losing discriminative power;

Structural dependency: Performance is highly dependent on the quality of the in-put graph, whereas real biomedical graphs often contain noise or missing edges;

Scalability and generalisation: Difficulty in processing large-scale graphs or generating embeddings for unseen nodes.

### 3.2. Core GNN Architectures for Molecular and Omics Graphs

In biomolecular systems, graph representations are not merely computational abstractions but reflect fundamental biological organisation principles. Nodes typically correspond to biologically meaningful entities such as genes, proteins, metabolites, or cells, while edges encode biochemical interactions, regulatory relationships, or spatial proximity. Consequently, the choice of graph neural network architecture is often guided by biological assumptions, including pathway modularity, hierarchical regulation, molecular specificity, and context-dependent interactions. GNNs are particularly well suited to biomolecular data because they enable structured information propagation that mirrors how biological signals are transmitted across molecular networks, rather than treating biological features as independent or exchangeable variables. In this section, we therefore reinterpret core GNN architectures through a biological lens, emphasising the modelling assumptions each design embodies and the types of biomolecular questions it is best suited to address. Specifically, this section outlines representative aggregation mechanisms, highlighting how each method family captures unique biological properties and supports a broad range of tasks in bioinformatics and computational biology.

#### 3.2.1. Mean Pooling-Based Aggregation

Mean pooling-based aggregation represents one of the earliest and most widely adopted paradigms in graph neural networks. From a biological perspective, this aggregation strategy embodies a simplifying but often biologically meaningful assumption: that the functional state of a biological entity can be approximated by the average influence of its local interaction partners. In many biomolecular systems, such averaging reflects collective or redundant effects, where no single interaction dominates system behaviour but coordinated activity across multiple neighbours determines function.

Formally, mean aggregation updates a node representation by computing the average of feature vectors from its neighbouring nodes, optionally including the node itself. This operation enforces local smoothness on the graph, encouraging connected biological entities to acquire similar representations. In protein–protein interaction networks, for example, mean pooling implicitly models the observation that proteins participating in the same functional module or pathway often share related biological roles. Likewise, in gene co-expression or regulatory graphs, mean aggregation reflects the assumption that gene activity is influenced by the collective regulatory environment rather than isolated regulators alone.

Mean pooling-based GNNs are particularly effective in biological settings characterised by relatively homogeneous interaction semantics and moderate network density. Representative architectures such as Graph Convolutional Networks (GCNs) and GraphSAGE with mean aggregation have been successfully applied to disease–gene association prediction, protein function annotation, and multi-omics patient stratification. In these tasks, averaging neighbour information can stabilise learning under noisy or incomplete interaction data, a common challenge in biological networks derived from high-throughput experiments.

However, the biological assumptions underlying mean aggregation also impose limitations. By treating all neighbours as equally informative, mean pooling neglects interaction specificity, regulatory directionality, and context-dependent effects that are central to many biological processes. For example, in gene regulatory networks, a master transcription factor may exert disproportionate influence compared to peripheral regulators, while in signalling pathways, upstream and downstream interactions are not interchangeable. Mean aggregation may therefore oversmooth biologically distinct signals, obscuring rare but functionally critical interactions such as tumour suppressor genes or low-degree regulatory hubs.

Consequently, while mean pooling-based aggregation provides a robust and interpretable baseline for biological graph learning, its suitability depends on the underlying biological task and graph structure. In practice, it often serves as a foundational model against which more expressive aggregation mechanisms are evaluated, such as attention-based, structure-aware, or hierarchical approaches. Understanding the biological assumptions encoded by mean aggregation is essential for interpreting its predictions and for determining when more specialised architectures are required to capture the complexity of biomolecular systems.

##### Neural-Fingerprint

**Figure 2:**
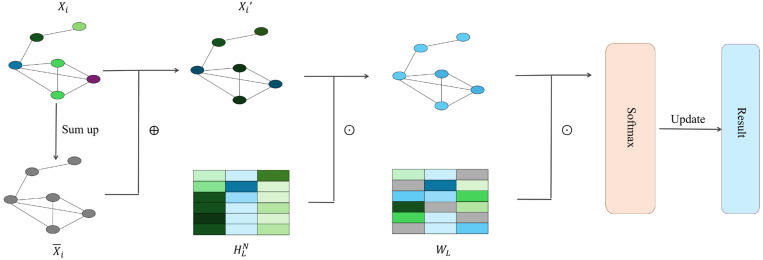
Neural-fingerprint’s procedure.

Traditionally, most machine learning pipelines for studying properties of novel molecules can only work on fixed-size input. Duvenaud et al. [58] introduced a differentiable neural network that can handle graphs of arbitrary size and shape, with vertices and edges respectively representing individual atoms and bonds of original molecules. Compared to circular fingerprints that compute a fixed-length binary molecular fingerprint vector for downstream analysis, Duvenaud et al. [58] used a graph as input to produce a real-valued vector representing information about atoms and their neighbouring substructures. In addition, discrete operations in circular fingerprints are replaced with differentiable operations. This network exhibits convolutional properties, specifically manifested in nodes with identical degrees sharing the same local filters to aggregate information from neighbouring nodes. The way of information aggregation between neighbours in the graph is shown as follows,

(18) $${\bar{X}}_{i}=\left(X_{i}+\sum_{j=1}^{N}X_{j}\right),$$

(19) $$X_{i}^{\prime }=\sigma \left({{\bar{X}}_{i}H}_{L}^{N}\right),$$

where $X_{i}$ is the features of the node i, N is the vertex degree of the node i, $H_{L}^{N}$ represents the weight matrixes for the vertex degree N in the $L^{th}$ iteration of information aggregation, and the σ is a smooth activation function in the single layer neural network. The smooth activation function can lead to similar activations for local molecular structure varying in different ways. For each time when features of a node are updated within an iteration of the whole graph, the fingerprint vector for the graph is updated accordingly as below,

(20) $$f=f+softmax\left(X_{i}^{\prime }W_{L}\right),$$

where f is the real-value fingerprint vector and $W_{L}$ is the output weight matrix in the $L^{th}$ iteration of information aggregation. The softmax is introduced to produce a classification label vector for each node, and the sum of these label vectors is the final fingerprint. Figure 2 illustrates how information flows in the neural network.

Compared to traditional fixed fingerprints, the neural graph fingerprints can achieve better predictive performance and only encode relevant features, considering the similarity between molecular fragments.

##### Diffusion-Convolution Neural Networks (DCNNs)

Many approaches have been proposed towards extending convolutional neural networks to graph-structured data since deep learning techniques have achieved promising results on image-based data. James, et.al. introduced a ‘diffusion-convolution’ operation that learns latent representations by performing a diffusion process across each node in the graph [59]. Their proposed diffusion-convolutional neural networks (DCNNs) are flexible and effective models that can handle general graphical data and various classification tasks, including node classification, edge classification and graph classification. The graph can be weighted or unweighted, directed, or undirected.

Each node is represented by F features. The vertices can be denoted as $N_{t}\times F$, where $N_{t}$ is the number of nodes in the graph $G_{t}$, and t identifies a graph in the set of T graphs $G=\left\{G_{t}|t\in 1\ldots T\right\}$. DCNNs use the power series of $P_{t}$ to model the graph diffusion process, where $P_{t}$ is a degree-normalised transition matrix for the graph $G_{t}$ which gives the probability of node i jumping to node j in one hop, and is defined by,

(21) $$P_{t}={D_{t}}^{-1}A_{t}\;,$$

where $A_{t}$ is the adjacency matrix of the graph $G_{t}$, and $D_{t}$ is the degree matrix.

In the node classification task, the information propagation through the graph $G_{t}$ can be formulated as follows:

(22) $$Z_{t}=f\left(W^{c}⊙P_{t}^{*}X_{t}\right),$$

where $X_{t}$ denotes $N_{t}\times F$ node features of the $G_{t}$, $P_{t}^{*}$ is an $N_{t}\times H\times F$ tensor representing the power series $\left[P_{t}^{1},\;\ldots ,\;P_{t}^{H-1}\right]$ with H as the number of hops of graph diffusion over F features, $W^{c}$ is the H×F trainable weight matrix, the operator ⊙ is an element-wise multiplication, and f is a nonlinear differentiable activation function. A dense layer is connected subsequently to output the hard prediction or a conditional probability distribution:

(23) $${\hat{Y}}_{t}=argmax\left(f\left(W^{d}⊙Z_{t}\right)\right),$$

(24) $$P\left(Y_{t}|X_{t}\right)=softmax\left(f\left(W^{d}⊙Z_{t}\right)\right),$$

where ${\hat{Y}}_{t}$ is the prediction for the label $Y_{t}$, and $P\left(Y_{t}|X_{t}\right)$ is a conditional probability distribution.

To extend the DCNNs to graph classification, the first equation is simply modified to take the mean activation over the nodes shown as follows,

(25) $$Z_{t}=f\left(W^{c}⊙1_{N_{t}}^{T}P_{t}^{*}X_{t}/N_{t}\right),$$

where $1_{N_{t}}$ is an $N_{t}\times 1$ vector of ones.

In the edge classification task, an edge is considered as a node which is adjacent to the pair of connected nodes $\left(i,\;j\right)$. The adjacency matrix $A_{t}$ for the graph $G_{t}$ is updated as follows,

(26) $$A_{t}^{\prime }=\left(\begin{array}{cc} A_{t} & B_{t}^{T}\\ B_{t} & 0 \end{array}\right),$$

where $B_{t}$ represents connections between the edge and its end nodes, and $B_{t}^{T}$ is the transpose of $B_{t}$. The new degree-normalised transition matrix $P_{t}^{\prime }$ is computed based on the updated adjacency matrix $A_{t}^{\prime }$.

##### GraphSAGE

Although many existing approaches have successfully extended convolution operations to fixed graphs where all nodes are included in network training, generalising such graph convolutional networks to unseen nodes under an inductive setting remains challenging. To address this challenge, Hamilton et al. [60] proposed a general framework, termed GraphSAGE (SAmple and aggreGatE), for learning aggregator functions that can generate embeddings on existing or extremely unseen nodes.

GraphSAGE has designed a unique forward propagation method, where at each iteration (or search depth), vertices aggregate information from their local neighbours, and as this process iterates, vertices obtain information from farther and farther away, which is shown in Algorithm 1. The forward propagation algorithm used by PinSAGE is the same as GraphSAGE, which is the theoretical foundation of PinSAGE.
**Algorithm 1:** GraphSAGE embedding generation (i.e., forward propagation) algorithm**Input**Graph G(V,ε); input features $\left\{x_{v},\forall v\in V\right\}$; depth *K*; weight matrices $W^{k},\;\forall k\in \left\{1,...K\right\}\;$; non-linearity σ; differentiable aggregator functions ${AGGREGATE}_{k},\;\forall \;k\;\in \left\{1,...K\right\}\;$; neighbourhood function $N:v\to 2^{V}$**Output**Vector representation $z_{v}$ for all v∈ V1$h_{v}^{0}\leftarrow x_{v},\forall v\in V$;2**For** k=1...K **do**3
**For** v∈ V **do**4

$h_{N_{\left(v\right)}}^{k}\leftarrow {AGGREGATE}_{k}\left(\left\{h_{u}^{k-1},\forall u\in N_{\left(v\right)}\right\}\right)$*,*5

$h_{v}^{k}\leftarrow \sigma \left(W^{k}\cdot CONCAT\left(h_{u}^{k-1},h_{N_{\left(v\right)}}^{k}\right)\right)$*,*6
**end**7
$h_{v}^{k}\leftarrow h_{v}^{k}/{\left\|h_{v}^{k}\right\|}_{2},\forall v\in V$*,*8**end**

9$z_{v}\leftarrow h_{v}^{k},,\forall v\in V$,

An unsupervised loss function was designed in the article, allowing GraphSAGE to train without task supervision.

(27) $$JG\left(z_{u}\right)=-\log\left(\sigma \left(z_{u}^{T}z_{v}\right)\right)-Q\cdot E_{v_{n}}\sim P_{n\left(v\right)}\log\left(\sigma \left(-z_{u}^{T}z_{v_{n}}\right)\right),$$

in which, $z_{u}$ is the embedding generated by GraphSAGE for node *u*; Node *v* is the “neighbour” that node *u* randomly walks to; σ is the sigmoid function; $P_{n}$ is the probability distribution of negative sampling, similar to negative sampling in Word2Vec; *Q* is the number of negative samples.

The representation $z_{u}$ of the input loss function in the text is generated from features containing a local neighbour of a vertex, unlike previous methods such as DeepWalk, which train a unique embedding for each vertex and then perform a simple embedding search operation to obtain it.

##### LightGCN

Although GCNs have shown promising results in learning latent features and performing prediction for collaborative filtering tasks, He et al. [61] empirically showed that feature transformation and nonlinear activation in the standard propagation rule can be removed in view of the user–item interaction graph. The reason is that, unlike those graphs where each node has rich features, the node in a user–item interaction graph is described by a one-hot ID without semantics. Their experiments showed that excluding feature transformation and nonlinear activation in Neural Graph Collaborative Filtering (NGCF) [62] surprisingly leads to substantial improvements in prediction accuracy. The embedding propagation rule in NGCF for each user u and node i is defined as follows,

(28) $$e_{u}^{\left(k+1\right)}=\sigma \left(W_{1}e_{u}^{\left(k\right)}+\sum_{i\in N_{u}}\frac{1}{\sqrt{\left|N_{u}\right|\left|N_{i}\right|}}\left(W_{1}e_{i}^{\left(k\right)}+W_{2}\left(e_{i}^{\left(k\right)}⊙e_{u}^{\left(k\right)}\right)\right)\right)\;,$$

(29) $$e_{i}^{\left(k+1\right)}=\sigma \left(W_{1}e_{i}^{\left(k\right)}+\sum_{u\in N_{i}}\frac{1}{\sqrt{\left|N_{i}\right|\left|N_{u}\right|}}\left(W_{1}e_{u}^{\left(k\right)}+W_{2}\left(e_{u}^{\left(k\right)}⊙e_{i}^{\left(k\right)}\right)\right)\right)\;.$$

where $e_{u}^{\left(k\right)}$ and $e_{i}^{\left(k\right)}$ respectively represent the learned embedding of user u and item i in the layer k, $N_{u}$ represents the neighbours of user node u, $N_{i}$ represents the neighbours of item node i, $W_{1}^{\left(k\right)}$ and $W_{2}^{\left(k\right)}$ represent a trainable weight matrix for performing feature transformation in the layer k, σ(·) denotes the nonlinear activation function, and ⊙ denotes the element-wise product. The embeddings learned by different layers for each user and item are respectively concatenated to obtain their final embedding. The prediction score for the interaction between user u and item i is generated by conducting an inner product between the final embeddings of user u and item i.

Inspired by the results of empirical experiments on NGCF [62], He, et al. [61] proposed a largely simplified GCN model, termed LightGCN, where only the core component of GCN’s ‘neighbourhood aggregation’ is reserved, but feature transformation and nonlinear activation function are removed. The embedding propagation rule in LightGCN is defined as follows,


(30) $$e_{u}^{\left(k+1\right)}=\sum_{i\in N_{u}}\frac{1}{\sqrt{\left|N_{u}\right|\left|N_{i}\right|}}e_{i}^{\left(k\right)},$$

(31) $$e_{i}^{\left(k+1\right)}=\sum_{u\in N_{i}}\frac{1}{\sqrt{\left|N_{i}\right|\left|N_{u}\right|}}e_{u}^{\left(k\right)}.$$

Different from most of the GCN models that combine information from neighbours and the target node, only the embeddings of neighbours contribute to information aggregation. The novelty of the model is that, instead of assigning a fixed non-semantic identifier for each user and item as input, the initial embeddings for them are the only trainable parameters in the model and can be learned by propagation.

The final representation for each entity in the graph is obtained by combining its embeddings learned in different layers:

(32) $$e_{u}=\sum_{k=0}^{K}\alpha_{k}e_{u}^{\left(k\right)};e_{i}=\sum_{k=0}^{K}\alpha_{k}e_{i}^{\left(k\right)},$$

where $\alpha_{k}\geq 0$ denotes the importance of the embedding in the k-th layer. Combining embeddings from different layers mitigates over-smoothing, enriches representation, and captures self-connections without an identity matrix.

By analysing the two layers of LightGCN, the smoothness of the embedding can be intuitively and reasonably demonstrated in the penultimate equation. Considering the embedding learned for a user in the second layer of LightGCN, it can be obtained as


(33) $$e_{u}^{\left(2\right)}=\sum_{i\in N_{u}}\frac{1}{\sqrt{\left|N_{u}\right|\left|N_{i}\right|}}e_{i}^{\left(1\right)}=\sum_{i\in N_{u}}\frac{1}{\sqrt{\left|N_{i}\right|}}\sum_{v\in N_{i}}\frac{1}{\sqrt{\left|N_{u}\right|\left|N_{v}\right|}}e_{v}^{\left(0\right)}\;.$$

The coefficient for two users $\left(u,\;v\right)$ who has co-interacted items that then can be obtained by


(34) $$c_{v\to u}=\frac{1}{\sqrt{\left|N_{u}\right|\left|N_{v}\right|}}\sum_{i\in N_{u}⋂N_{v}}\frac{1}{\sqrt{\left|N_{i}\right|}}.$$

The interpretation of the coefficient is straightforward: (1) the more co-interacted items they share, the more effects they have on each other; (2) the more popular the item is, the less influence it has on the smoothing strength between two users; and (3) the more active a user is, the less influence that user has on other users.

##### Multi-Graph Convolutional Collaborative Filtering (Multi-GCCF)

**Figure 3:**
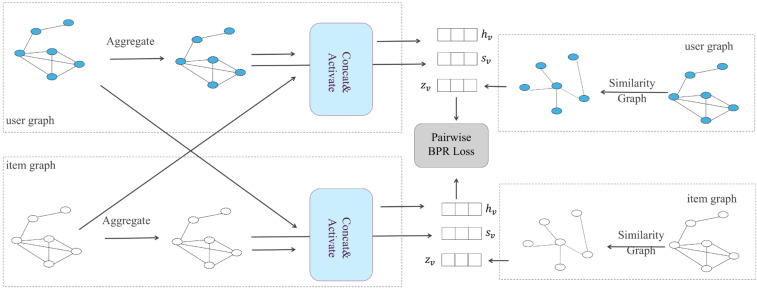
The overall architecture of Multi-GCCF.

A recommendation system is one of the applications of a graph convolutional network. The user–item interaction in a recommendation scenario can be represented by a bipartite graph whose nodes can be divided into two disjoint and independent sets: user nodes and item nodes. A GCN-based recommendation system framework, Multi-GCCF, proposed by Sun et al. [63], showed that using separate aggregation and transformation functions for user nodes and item nodes can learn more precise embeddings as the intrinsic difference between users and nodes is captured. This framework consists of three components: a bipartite convolutional graph (Bipar-GCN) representing the user–item interaction, two convolutional graphs with a Multi-Graph Encoding (MGE) layer for generating additional embedding nodes and a skip-connection mechanism. The architecture of Multi-GCCF is illustrated in Figure 3. The embeddings of the user node u in $k_{th}$ layer of the Bipar-GCN can be represented as:


(35) $$h_{u}^{k}=\sigma \left(W_{u}^{k}\cdot \left[h_{u}^{k-1};h_{N\left(u\right)}^{k-1}\right]\right),\;X_{u}^{0}=e_{u},$$

(36) $$h_{N\left(u\right)}^{k-1}={AGGREGATOR}_{u}\left(\left\{h_{v}^{k-1},v\in N\left(u\right)\right\}\right),\;{AGGREGATOR}_{u}=\sigma \left(MEAN\left(\left\{h_{v}^{k-1}\cdot Q_{u}^{k},\;v\in N\left(u\right)\right\}\right)\right),$$

where $e_{u}$ are the initial user embeddings, $\left[\;;\right]$ is vector concatenation, $\sigma \left(\cdot \right)$ is the tanh activation function, $W_{u}^{k}$ represents the transformation weight matrix shared across all user nodes and $v\in N\left(u\right)$ represents all neighbouring item nodes of the user node u. Information from neighbouring nodes is aggregated through a mean aggregator MEAN and an aggregator weight matrix $Q_{u}^{k}$ shared across all user nodes in the $k^{th}$ layer.

Similarly, the embeddings of the item node v in $k_{th}$ layer of the Bipar-GCN can be represented as:


(37) $$h_{v}^{k}=\sigma \left(W_{v}^{k}\cdot \left[h_{v}^{k-1};h_{N\left(v\right)}^{k-1}\right]\right),\;X_{v}^{0}=e_{v},$$

(38) $$h_{N\left(v\right)}^{k-1}={AGGREGATOR}_{v}\left(\left\{h_{u}^{k-1},\;u\in N\left(v\right)\right\}\right),\;{AGGREGATOR}_{v}=\sigma \left(MEAN\left(\left\{h_{u}^{k-1}\cdot Q_{v}^{k},\;u\in N\left(v\right)\right\}\right)\right).$$

The user–user graph and item–item graph constructed by computing pairwise cosine similarities aim to generate additional embedding for user nodes and item nodes to alleviate the data sparsity problem. The MGE layer consists of a one-hope convolution layer and a sum aggregator. The embeddings for target nodes can be represented as:

(39) $$z_{u}=\sigma \left(\sum_{i\in N^{\prime }\left(u\right)}e_{i}\cdot M_{u}\right),\;z_{v}=\sigma \left(\sum_{j\in N^{\prime }\left(v\right)}e_{j}\cdot M_{v}\right),$$

where $N^{\prime }\left(u\right)$ represents the neighbours of user node u, $N^{\prime }\left(v\right)$ represents the neighbours of item node v, $M_{u}$ and $M_{v}$ are learnable aggregation weight matrixes in two graphs. Since the embeddings generated in Bipar-GCN and MGE are primarily learned by aggregating neighbouring information, a skip-connection is introduced to re-emphasise the initial node embeddings. Experiments showed that summarising the obtained three embeddings via element-wise sum achieves better performance than via concatenation and attention mechanism [63].

#### 3.2.2. Attention-Based Aggregation

Attention-based aggregation extends mean pooling by relaxing the assumption that all biological interactions contribute equally to a node’s functional state. From a biological perspective, attention mechanisms encode the hypothesis that interaction strength, regulatory influence, or functional relevance varies across neighbours, and that selectively weighting these interactions is critical for capturing biologically meaningful dependencies. This assumption aligns closely with many biomolecular systems, where a small number of key regulators, binding partners, or signalling interactions exert disproportionate influence.

In attention-based graph neural networks, such as Graph Attention Networks (GATs), node representations are updated through a weighted combination of neighbour features, where attention coefficients are learned as a function of node attributes and local graph structure. Biologically, these coefficients can be interpreted as context-dependent measures of interaction importance. For example, in gene regulatory networks, attention weights may reflect differential regulatory influence among transcription factors; in protein–protein interaction networks, they may prioritise interfaces critical for complex formation or signal transduction.

Attention-based aggregation is particularly well suited for biological graphs characterised by heterogeneity, sparsity, and interaction specificity. In disease–gene association studies, attention mechanisms can emphasise disease-relevant subnetworks while suppressing background interactions. In protein structure and function modelling, attention can highlight spatially or chemically significant residue interactions over structurally adjacent but functionally neutral contacts. Similarly, in multi-omics integration, attention allows models to dynamically balance contributions from different molecular layers, such as transcriptomic, epigenomic, and proteomic signals, depending on biological context.

Beyond predictive performance, attention-based GNNs offer enhanced interpretability, a property of central importance for biological and clinical applications. Learned attention weights provide a transparent mechanism for identifying influential nodes, edges, or pathways, thereby supporting hypothesis generation and experimental follow-up. For biologists and physicians, this interpretability facilitates reasoning about why a model associates a gene with a disease, prioritises a drug–target interaction, or stratifies patients into molecular subtypes.

Nevertheless, attention-based aggregation also introduces challenges that must be considered in biological settings. Attention weights are learned from data and may reflect dataset biases, noise, or confounding correlations rather than true causal influence. Moreover, attention scores are not guaranteed to correspond to mechanistic importance unless supported by biological priors or experimental validation. Computationally, attention mechanisms increase model complexity and may limit scalability when applied to very large biological graphs, such as single-cell atlases or population-scale interaction networks.

In summary, attention-based aggregation provides a biologically motivated extension of mean pooling by modelling interaction heterogeneity and context-dependent influence. When applied judiciously and interpreted with appropriate biological caution, attention mechanisms enable GNNs to move beyond uniform smoothing toward more selective and interpretable representations of complex biomolecular systems.

##### Graph Attention Networks (GAT)

Most prior approaches that operate convolution operations on graph-structured data explicitly specify the importance of neighbours to a target node based on the structure of graphs or as a costly learnable weight matrix when aggregating information across neighbourhoods. Motivated by the fact that the self-attention mechanism has shown tremendous ability to achieve state-of-the-art performance in machine translations [64], Veličković et al. [65] proposed graph attention networks (GAT) that utilise self-attention to efficiently compute the attention coefficients across pairs of nodes by using a single-layer neural network. Let $h=\left\{{\vec{h}}_{1},\;{\vec{h}}_{2},\;\ldots ,\;{\vec{h}}_{N}\right\},\;{\vec{h}}_{i}\in R^{F}$ and $h^{\prime }=\left\{{\vec{h}}_{1}^{\prime },\;{\vec{h}}_{2}^{\prime },\;\ldots ,\;{\vec{h}}_{N}^{\prime }\right\},\;{\vec{h}}_{i}^{\prime }\in R^{F^{\prime }}$ be node features before and after an aggregation of information, where F and $F^{\prime }$ are the number of features. The computation of the attention coefficient for two connected nodes $\left(i,\;j\right)$ can be expressed as

(40) $$e_{ij}=a\left(W{\vec{h}}_{i},\;W{\vec{h}}_{j}\right),$$

where $W\in R^{F\times F^{\prime }}$ denotes a shared learnable linear transformation matrix, $a\;:\;R^{F^{\prime }}\times R^{F^{\prime }}\to R$ is a shared attentional mechanism, an $e_{ij}$ is an unnormalised coefficient which weights the importance of neighbouring node j to target node i. The coefficients are then normalised using the softmax function to enable comparison across neighbourhoods of different nodes:

(41) $$\alpha_{ij}=\frac{exp\left(e_{ij}\right)}{\sum_{k\in N_{i}}\exp\left(\alpha_{ik}\right)},$$

where $k\in N_{i}$ denotes a set of neighbouring nodes of node i. The aggregation of information across the neighbourhood of node i can be formulated as follows,

(42) $${\vec{h}}_{i}^{\prime }=\sigma \left(\sum_{k\in N_{i}}\alpha_{ij}W{\vec{h}}_{j}\right),$$

where $\sigma \left(\cdot \right)$ is a non-linear activation function. Multi-head attention mechanism is applied by combining several self-attention heads obtained from independently replicating the penultimate equation K times, aiming to make the learning process of self-attention more stable and accurate:

(43) h→i′=Kk=1σ∑k∈NiαijkWkh→j,

where $W^{k}$ denotes the linear transformation in the $k^{th}$ replica of the self-attention mechanism $a^{k}$, the operator ∥ represents the concatenation operation, and the dimension of the ${\vec{h}}_{i}^{\prime }$ is $K\times F^{\prime }$. The aggregated features can also be summarised by taking the element-wise mean of feature vectors before a non-linear operation:

(44) $${\vec{h}}_{i}^{\prime }=\sigma \left(\frac{1}{K}\sum_{k=1}^{K}\sum_{k\in N_{i}}\alpha_{ij}^{k}W^{k}{\vec{h}}_{j}\right).$$

The GAT layer satisfies many properties that a standard convolution operation has, including a fixed number of parameters regardless of the structure of the graph, and information aggregation from the local neighbourhood based on learned attentional coefficients and the capability to handle inductive problems. Compared to prior GCN methods, it implicitly assigns different importance to a node and its neighbours and enables efficient computation without eigen decomposition or other costly matrix operations.

Despite its flexibility, the original GAT exhibits a known limitation: the attention coefficients are computed using a static linear transformation of node features, which can restrict the expressive power of attention and prevent effective discrimination between structurally similar neighbours [66]. This limitation may lead to reduced sensitivity to feature interactions and neighbourhood context, particularly in deep or heterogeneous biological graphs.

To address this issue, Graph Attention Network v2 (GATv2) [66] was proposed as a refinement that allows attention scores to be computed as a dynamic function of interacting node features, thereby increasing expressiveness and mitigating attention collapse. GATv2 has been shown to provide more stable optimisation and improved performance across diverse graph learning tasks, and it has therefore become the default attention-based architecture in many recent bioinformatics applications.

Overall, attention-based GNNs provide an adaptive mechanism for weighting biological relationships, but their effectiveness depends critically on the design of the attention function and its ability to capture context-dependent molecular or regulatory interactions.

##### Factorizable Graph Convolutional Networks (FactorGCN)

In many real-world graphs, a pair of nodes is only connected via a single collapsed edge, even though the relations between them are heterogeneous. This might lead to the concealment of latent intrinsic connections. The work of Yang et al. [67] focuses on factorising relations on the graph level to learn those latent disentangled features, which shows improvement in performance for downstream tasks. Their proposed GCN framework, termed FactorGCN, is made up of several layers, each of which contains three steps: disentangling a single graph into several factorised graphs, performing information aggregation independently of each graph from the other and merging latent disentangled features together by concatenation to form the final features for nodes in the original graph. By stacking several such disentangle layers, FactorGCN can decompose the input data at different levels in different relation spaces, allowing information propagation to perform in disjoint spaces.

In the disentangling step, factor graphs are generated separately through the attention mechanism, where the coefficients $E_{ije}$ for a pair of nodes $\left(i,\;j\right)$ in the factor graph are learned, and with the range of $\left[0,\;1\right]$:

(45) $$E_{ije}=1/\left(1+e^{-\Psi_{e}\left(h_{i}^{\prime },\;h_{j}^{\prime }\right)}\right);\;h^{\prime }=Wh,$$

where h denotes the set of nodes with features, W is a linear transformation matrix, $\Psi_{e}$ is a one-layer MLP that takes the features of the pair of nodes $\left(i,\;j\right)$ as input to compute the attention score of the edge for factor graph e, $E_{ije}$ is the normalised attention score of the edge from node i to node j, $h^{\prime }$ is the transformed features of nodes shared across all $\Psi_{*}$. An additional head is introduced to the disentangle layer to make sure that no factor graphs are redundant and that each factor graph can be distinguished to the rest.

In the aggregation step, the process of information aggregation takes place independently in different factor graphs as follows,

(46) $$h_{i}^{{\left(l+1\right)}_{e}}=\sigma \left(\sum_{j\in N_{i}}E_{ije}/c_{ij}h_{j}^{\left(l\right)}W^{\left(l\right)}\right)\;,\;c_{ij}={\left(\left|N_{i}\right|\left|N_{j}\right|\right)}^{1/2},$$

where $h_{i}^{{\left(l+1\right)}_{e}}$ denotes the updated features of node i in the ${\left(l+1\right)}^{th}$ layer of the factor graph e, $E_{ije}$ is the coefficient of the edge in the factor graph e, $c_{ij}$ is a normalisation term, and $W^{\left(l\right)}$ is the same linear transformation matrix as the one used in the disentangling step. All disentangled features derived from the aggregation step are concatenated to produce the final form of features for node i as follows,

(47) hil+1=Nee=1hil+1e,

where $h_{i}^{\left(l+1\right)}$ is the final form of features for node i, $N_{e}$ is the number of factor graphs, and the operator ∥ represents the concatenation operation on disentangled features.

#### 3.2.3. Structure-Aware/Spatial-Aware Aggregation

Structure-aware and spatial-aware aggregation mechanisms extend graph neural networks beyond abstract connectivity by explicitly incorporating geometric, topological, and spatial constraints that are intrinsic to biological systems. From a biological standpoint, these approaches reflect the fundamental assumption that the function of biomolecules and cells is not determined solely by interaction existence but by the spatial organisation, relative positioning, and structural context in which interactions occur.

In many biological settings, spatial proximity and geometric arrangement play a decisive role. In molecular graphs, chemical reactivity and binding affinity depend on three-dimensional conformation rather than graph connectivity alone. In protein structures, residue interactions are governed by spatial distance, orientation, and physicochemical compatibility, while in tissues or spatial transcriptomics data, cellular behaviour is shaped by local neighbourhood architecture and microenvironmental context. Structure-aware aggregation mechanisms are designed to capture these constraints by modulating message passing according to geometric features, spatial distance metrics, or higher-order structural descriptors.

Computationally, structure-aware GNNs incorporate additional information such as inter-node distances, angles, relative coordinates, or topological roles when aggregating neighbour information. Rather than treating neighbours as an unordered set, these models preserve structural patterns that distinguish biologically meaningful interactions from incidental proximity. For example, in protein structure modelling, spatial-aware aggregation enables the network to prioritise contacts that are close in three-dimensional space but distant along the amino acid sequence, a key requirement for identifying functional sites and allosteric interactions. Similarly, in molecular property prediction, geometry-aware aggregation supports learning representations that respect chemical validity and stereochemical constraints.

Structure-aware aggregation is also critical for modelling higher-level biological organisation. In cellular and tissue-scale graphs, spatial-aware GNNs can capture gradients, niches, and boundary effects that influence cell fate and function. In brain networks and other anatomical systems, preserving spatial topology allows models to distinguish between structurally conserved regions and functionally specialised modules. These capabilities are essential for moving from pattern recognition toward mechanistic interpretation, particularly in applications involving spatial omics, histopathology, and organ-level modelling.

Despite their biological relevance, structure-aware and spatial-aware approaches introduce additional challenges. Incorporating geometric information increases model complexity and computational cost, and spatial data are often incomplete, noisy, or platform dependent. Moreover, structural features alone do not guarantee biological causality; spatial correlation may arise from shared developmental origin or measurement artefacts rather than direct functional interaction. Consequently, structure-aware aggregation is most effective when combined with biological priors, experimental annotations, or complementary data modalities that help disambiguate structural association from functional relevance.

In summary, structure-aware and spatial-aware aggregation mechanisms represent a critical step toward biologically faithful graph learning. By embedding spatial organisation and structural constraints directly into message passing, these approaches enable GNNs to better reflect the physical and organisational principles governing biomolecular systems, thereby enhancing both predictive accuracy and biological interpretability.

##### Spatial Graph Convolution Network (SGCN)

Most graph convolutional networks ignore nodes’ spatial positions despite assigning different weights to neighbours. Danel et al. [54] proposed a model called SGCN, which processes graph-based data with spatial features. The experiments conducted on SGCN have shown that introducing positional features of nodes into models consistently leads to improvements in performance across tasks. In addition, identification of each node by its coordinates allows for performing data augmentation with random rotation on the given dataset, which can extend the dataset and, in consequence, improve network generalisation with a limited amount of data. Compared to a typical graph convolution, information of neighbours is aggregated with spatial relations being considered:

(48) $${\bar{X}}_{i}\left(U,\;b\right)=\sum_{j\in N_{i}}ReLU\left(U^{T}\left(p_{j}-p_{i}\right)+b\right)⊙X_{j},$$

where $U\in R^{t\times d}$, $b\in R^{d}$ are trainable parameters, $p_{i}\in R^{t}$ is the coordinates of the node in the spatial space, t is the dimension of the coordinate, d is the dimension of $X_{j}$ and the operator ⊙ is element-wise multiplication. The relative position between adjacent nodes is transformed through both linear and non-linear operations to be a scalar on the standard features of a neighbouring node. SGCN can be easily generalised to typical graph convolution by setting spatial features to 0.

Analogous to applying multiple filters on feature maps in classical convolution, multiple transformations can be operated on each node. For this purpose, the new feature representation of a node is defined by:

(49) $${\bar{X}}_{i}\left(U,\;B\right)={\bar{X}}_{i}\left(U^{\left(1\right)},\;b^{\left(1\right)}\right)⊕\;\cdot \;\cdot \;\cdot \;⊕{\bar{X}}_{i}\left(U^{\left(k\right)},\;b^{\left(k\right)}\right),$$

where $U=\left[\;U^{\left(1\right)},\ldots ,\;U^{\left(k\right)}\right]$ and $B=\left[\;b^{\left(1\right)},\ldots ,\;b^{\left(k\right)}\right]$ define k filters, and the operator ⊕ is vector concatenation.

#### 3.2.4. Feature Interaction-Enhanced Aggregation

Feature interaction-enhanced aggregation mechanisms are motivated by the biological principle that functional outcomes often arise from the combined and non-linear interaction of multiple molecular factors rather than from independent effects. In biomolecular systems, regulatory logic is frequently combinatorial: gene expression depends on the coordinated action of multiple transcription factors, protein function emerges from the interplay of residues and domains, and cellular phenotypes reflect interactions across diverse molecular layers. Capturing such synergistic or antagonistic effects is essential for modelling biological complexity.

In graph neural networks, feature interaction-enhanced aggregation explicitly models higher-order relationships among node features during message passing. Rather than aggregating neighbour features through simple linear combinations, these approaches incorporate mechanisms such as feature-wise transformations, bilinear or tensor interactions, gating functions, or cross-feature attention to capture non-additive dependencies. Biologically, this enables models to represent conditional effects, where the influence of one molecular feature depends on the presence or state of others.

These mechanisms are particularly relevant for biological tasks characterised by combinatorial regulation and context-dependent effects. In gene regulatory networks, feature interaction-enhanced aggregation can model cooperative or competitive binding of transcription factors and the integration of epigenetic signals. In protein structure and function analysis, interactions between amino acid properties, secondary structure elements, and spatial context jointly determine catalytic activity or binding specificity. In multi-omics integration, such aggregation allows models to capture cross-modal interactions, for example, how genetic variation modifies transcriptional responses or how epigenetic states modulate protein abundance.

By modelling feature interactions explicitly, these GNN architectures improve both expressive power and biological interpretability. Interaction terms can highlight feature combinations that drive predictions, offering insights into potential molecular mechanisms and facilitating hypothesis generation. For biologists and clinicians, such models provide a pathway toward understanding not only which genes or molecules are important but how their interactions contribute to observed phenotypes or disease states.

However, feature interaction-enhanced aggregation also introduces additional complexity and risks. Higher-order interaction models are more prone to overfitting, particularly in biological datasets where sample sizes are limited and noise is pervasive. Moreover, not all learned feature interactions correspond to true biological mechanisms; some may reflect dataset-specific correlations or technical artefacts. Effective application therefore requires careful regularisation, biologically informed feature design, and validation against experimental or curated knowledge.

In summary, feature interaction-enhanced aggregation addresses a central limitation of simpler GNN architectures by capturing the combinatorial and context-dependent nature of biomolecular regulation. When applied with appropriate biological constraints and interpretive caution, these approaches enable more faithful modelling of complex biological processes and support mechanistic insight beyond additive representations.

##### Graph Convolutional Networks with Categorical Node Features (CatGCN)

Node features of many real-world applications, such as user profiling and recommendation systems, are categorical. However, the feature interactions are often ignored by simply applying a sum of feature embeddings as the initial node representation in most GCNs. Considering feature interaction is important as it carries information for predictive analytics. For example, people with feature pairwise gender_age =[male, 20−25] tend to be digital enthusiasts. In addition, the quality of initial node representations can largely affect the learning of the follow-up graph convolution and the model performance. To integrate interactions among the features into initial node representations for better learning, Chen et al. [68] proposed a novel GCN model named CatGCN. This model is specially designed for categorical node features.

In CatGCN, two kinds of feature interactions are integrated into node representations: (1) local interaction between feature pairs [69,70,71] and (2) global interaction amongst the whole feature set. To mine the relations among features in the embedding space, non-zero features are first projected into feature embeddings. The local feature interactions are captured by applying the element-wise product of feature embeddings [69] as formulated below,

(50) $$h_{l}=\sum_{i,j\in S\;\&\;j>i\;}e_{i}⊙e_{j}=\frac{1}{2}\left[{\left(\sum_{i\in S}e_{i}\right)}^{2}-{\left(\sum_{j\in S}e_{j}\right)}^{2}\right]\;,$$

where S denotes a set of nonzero features of a node, $e_{i}\in R^{D}$ denotes the embedding of feature i, and $h_{l}$ denotes the learned initial node representations that consider information of local feature interaction. To capture global feature interactions, an artificial complete graph $\left(P,E\right)$ is constructed to model the structure of the features in the embedding space, where $P\in R^{\left|S\right|\times \left|S\right|}$ is the adjacency matrix and $E\in R^{\left|S\right|\times d}$ Represents feature embeddings. Graph convolution is performed on the artificial graph to learn the initial node representations as follows,

(51) $$h_{g}=p\left(\sigma \left(\tilde{P}EW\right)\right),\;\tilde{P}=D^{-\frac{1}{2}}\left(P+\rho I\right)D^{-\frac{1}{2}}=\frac{P+\rho I}{\left|S\right|+\rho }\;,$$

where $\tilde{P}$ is the normalised adjacency matrix with probe coefficient ρ, W is a trainable weight matrix, $D=\left(\left|S\right|+\rho \right)I$ is the degree matrix with I as the identity matrix, $\sigma \left(\cdot \right)$ is an activation function, $p\left(\cdot \right)$ is a pooling function across features, and $h_{g}$ denotes the learned initial node representations that consider information of global feature interaction.

The initial node representations learned through two kinds of feature interactions are projected back to the label space and fused through an aggregation layer as follows,

(52) $$h=\alpha h_{g}^{\prime }+\left(1-\alpha \right)h_{l}^{\prime },\;h_{g}^{\prime }=\sigma \left(W_{g}h_{g}+b_{g}\right),\;h_{l}^{\prime }=\sigma \left(W_{l}h_{l}+b_{l}\right)\;,$$

where $W_{g}$ and $W_{l}$ are projection matrices, $\alpha \in \left[0,\;1\right]$ is a hyper-parameter to fuse node representations, and h is the fused initial node representation. CatGCN takes the fused initial node representations as input and performs pure neighbourhood aggregation (PNA) over the graph to obtain the final node representations. The prediction label for a node is the distribution of its final node representations computed by softmax function, while the ground-truth of the node is a one-hot vector.

#### 3.2.5. Probabilistic/Generative Aggregation

Probabilistic and generative aggregation mechanisms are motivated by a fundamental characteristic of biological systems: biological observations are inherently uncertain, incomplete, and context dependent. Molecular interactions may be transient, regulatory relationships may vary across conditions, and experimental measurements are subject to technical noise and biological variability. From a biological perspective, deterministic aggregation alone is often insufficient to capture such stochasticity and latent structure.

Probabilistic graph neural networks incorporate uncertainty directly into representation learning by modelling node states, edge relationships, or aggregation outcomes as random variables rather than fixed quantities. Variational graph autoencoders, Bayesian GNNs, and stochastic message-passing frameworks exemplify this paradigm. Biologically, these approaches align with the recognition that observed networks, such as protein–protein interaction maps or gene regulatory graphs. This represent incomplete and noisy samples of an underlying biological process rather than exact ground truth.

Generative aggregation extends this perspective by learning to model the data-generating process itself. Instead of only predicting labels or properties, generative GNNs aim to infer latent biological structure, simulate plausible network configurations, or generate new molecular or interaction patterns consistent with learned constraints. In molecular design and protein engineering, generative aggregation enables exploration of chemical or structural space while respecting physicochemical and biological priors. In regulatory network inference, it supports the identification of hidden regulators, missing interactions, or alternative network topologies that are consistent with observed data.

These probabilistic and generative approaches offer distinct advantages for biological and clinical research. By explicitly modelling uncertainty, they enable confidence estimation and risk-aware decision making, which are essential for experimental prioritisation and translational applications. For example, probabilistic predictions can guide which gene–disease associations or drug–target interactions warrant experimental validation, while generative models can propose candidate molecules or regulatory hypotheses for downstream testing.

However, the biological interpretability of probabilistic and generative aggregation requires careful consideration. Learned latent variables may not correspond directly to identifiable biological entities, and generated structures may reflect statistical plausibility rather than mechanistic feasibility. Moreover, these models often demand greater computational resources and larger datasets, posing challenges in data-scarce biological domains. Integrating biological constraints, curated knowledge, and experimental feedback is therefore critical for ensuring that probabilistic inference remains biologically meaningful.

In summary, probabilistic and generative aggregation mechanisms provide a powerful framework for modelling uncertainty, latent structure, and hypothesis space in biomolecular systems. By moving beyond deterministic representations, these approaches enable graph neural networks to support not only prediction but also uncertainty-aware inference and hypothesis generation, aligning closely with the needs of experimental biology and precision medicine.

##### Variational Graph Convolutional Network (VGCN)

Z Ren et al. [72] proposed the VGCN model, which integrates a variational structure with a dynamic edge weight learning mechanism. This approach not only learns latent representations of textual data but also forms a continuous high-dimensional graph structure distribution, significantly enhancing the expressiveness of relative information.

**Figure 4:**
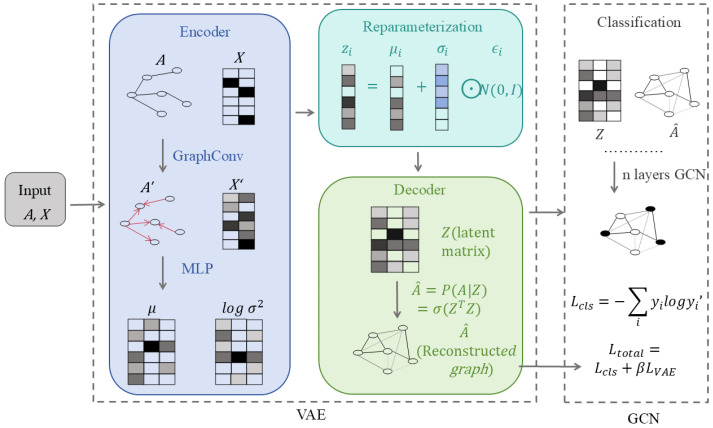
The pipeline of VGCN.

As shown in Figure 4, the graph is first processed through a layer of graph convolutional networks to aggregate information from neighbouring nodes and learn low-dimensional feature representations. Subsequently, a variational structure is introduced, where nodes pass through two fully connected layers to obtain mean and variance representations for each node’s features:

(53) $$\mu =f_{\mu }\left(H\right),log\sigma^{2}=f_{\sigma }\left(H\right)$$

Next, the Reparameterisation Trick is applied to sample from this distribution, yielding the latent vector representations for each node.

(54) $$z=\mu +\sigma ⊙\epsilon ,\epsilon \sim N\left(0,I\right)$$

This step transforms the determined node features into random variables conforming to a specific distribution, enhancing the model’s expressive power and robustness.

(55) A^ij=σ(zi⊤zj)

The sampled latent representations are then fed into a decoder tasked with reconstructing the original graph structure information.

Concurrently, the model employs a dynamic edge weight learning mechanism, treating the edge weights in the adjacency matrix as learnable parameters. These are optimised via gradient descent during training, enabling the graph structure to adaptively align with downstream classification tasks.

(56) $$A_{ij}^{\left(t+1\right)}=\sigma \left(W_{e}^{⊤}\left[h_{i}^{\left(t\right)}∥h_{j}^{\left(t\right)}\right]+b_{e}\right)$$

Experiments conducted on the THUCNews (long-form text) and Toutiao (short-form text) datasets demonstrate that VGCN (referred to as VSD-GCN in the table) achieves the best performance compared to multiple baseline models.

#### 3.2.6. Other Clustering Methods

Beyond generalised aggregation and propagation mechanisms, certain studies focus on reconstructing or transforming the graph structure itself to accommodate standard deep learning modules, tailored to specific data formats or task requirements. The core principle of such approaches is to map unordered, variable-length graph data into ordered, fixed-length sequence representations, thereby bridging graph neural networks with classical CNN or Transformer architectures. Whilst sacrificing some native topological properties of the graph, these methods demonstrate exceptional engineering utility in tasks such as graph classification and molecular property prediction. They are particularly well-suited for multimodal biomedical scenarios requiring integration with existing visual or linguistic models.

##### PATCHY-SAN

**Figure 5:**
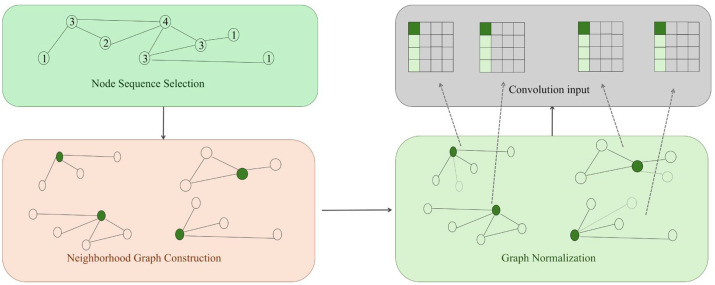
The pipeline of PATCHY_SAN.

Analogous to the behaviour of the receptive field in image-based CNNs that moves over locally connected regions of non-edge pixels of the image in a certain order, from left to right and top to bottom, Niepert et al. [9] proposed a general framework, termed PATCHY-SAN, that follows this idea to learn feature representations for an arbitrary graph, shown in Figure 5. For a given graph, a node sequence is selected via a graph labelling procedure. For the top ω nodes in the sequence, their neighbouring nodes are selected to construct neighbourhood graphs, where a graph labelling procedure is taken to rank the nodes and create the normalised receptive fields of size k. Graph normalisation refers to assigning nodes with similar structural roles in different graphs to a similar relative position in the vector space representation. Each attribute of an entity (node or edge) matches an input channel in the convolution. Finally, a 1-D convolutional layer can be applied to nodes according to the normalised receptive fields. PATCH-SAN provides a method to create a receptive field for each selected node in the graph so that the standard convolution operation can be performed.

##### Learnable Graph Convolutional Network (LGCN)

**Figure 6:**
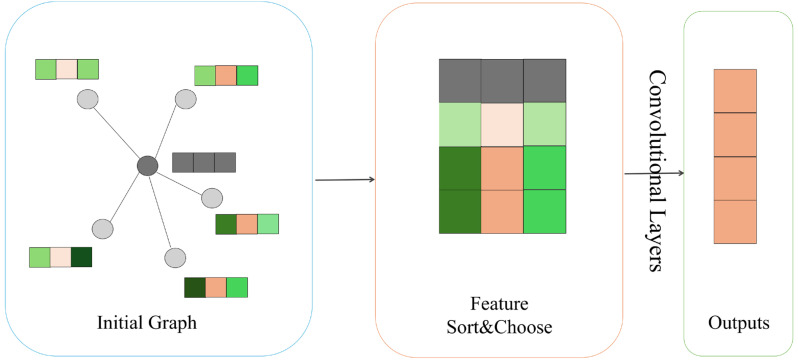
The pipeline of LGCL.

As mentioned above in PATCHY-SAN, the standard convolution operations require a fixed number of ordered units in the receptive fields, while the degree of node is not fixed, and nodes are not ordered in the graphical data. Gao et al. [73] proposed the learnable graph convolutional layer (LGCL) to achieve transformation from graph to grid-like data, where a standard 1-D convolution operation can be applied. The updating process of features over an LGCL can be formulated as

(57) $${\tilde{X}}_{l}=g\left(X_{l},\;A,\;k\right),\;X_{l+1}=c\left({\tilde{X}}_{l}\right)$$

where $X_{l}$ represent feature representations for all nodes in $l_{th}$ layer, A is the adjacency matrix, k is the number of neighbouring nodes to be selected for each feature, $g\left(\cdot \right)$ is an operation that prepares grid-like data for further regular convolution, and $c\left(\cdot \right)$ denotes 1-D convolutions that aggregate information from neighbouring nodes. Figure 6 illustrates the structure of LGCL.

Given a node, its neighbouring nodes’ feature values are ranked separately and independently. For each feature, the k-largest values are reserved and concatenated with the feature values of the target node. Through the selection process, $g\left(\cdot \right)$ transforms $X_{l}\in R^{N\times 1\times C}$ to ${\tilde{X}}_{l}\in R^{N\times \left(k+1\right)\times C}$, where N is the training batch size, k+1 is the spatial size, and C is the number of features corresponding to channels in convolution. 1-D convolution operations can be applied to the grid-like data ${\tilde{X}}_{l}$ to aggregate information and output the next-level feature representations $X_{l+1}\in R^{N\times 1\times D}$, where D denotes the number of features for each node in the new feature space.

A learnable graph convolutional network (LGCN) consists of several LGCLs stacked with skip concatenation connections, which enable a deeper design of a network [74,75], and a fully connected layer at the end for node classification.

#### 3.2.7. Pooling and Hierarchical Representation Methods

Pooling and hierarchical representation methods are motivated by a defining characteristic of biological systems: biological organisation is inherently hierarchical. Atoms assemble into residues, residues form functional domains, proteins participate in complexes and pathways, cells organise into tissues, and tissues coordinate to produce organism-level phenotypes. Effective computational models must therefore capture not only local interactions but also how information aggregates and propagates across biological scales.

In graph neural networks, pooling mechanisms aim to summarise node-level representations into higher-level abstractions, enabling learning at multiple resolutions. From a biological perspective, pooling reflects the assumption that meaningful biological function often emerges at intermediate or higher organisational levels rather than at individual nodes alone. For example, disease phenotypes are more closely associated with dysregulated pathways or modules than with single genes, and drug response is frequently mediated by coordinated network effects rather than isolated molecular interactions.

Hierarchical GNNs implement this principle by progressively coarsening graphs, grouping nodes into clusters or super-nodes based on structural, functional, or learned similarity. Such approaches enable models to learn representations that reflect biological modules, domains, or cell populations, while preserving the relationships between these higher-order units. In protein structure analysis, hierarchical pooling can aggregate residue-level features into domain-level representations, supporting function prediction and binding site identification. In systems biology and multi-omics analysis, hierarchical representations facilitate pathway-level inference and patient stratification by capturing coordinated molecular programmes.

Different pooling strategies encode different biological assumptions. Global pooling methods, such as sum or mean pooling, assume that overall system behaviour can be summarised by aggregate activity, which may be appropriate for tasks like molecular property prediction. In contrast, adaptive or structure-aware pooling methods aim to identify salient substructures, reflecting the biological intuition that specific modules or subnetworks drive functional outcomes. Learned pooling mechanisms can prioritise biologically relevant nodes or interactions, enabling the model to focus on key regulators, functional domains, or disease-associated modules.

Hierarchical representation learning also enhances interpretability, a central requirement for biological and clinical applications. By explicitly modelling intermediate organisational levels, hierarchical GNNs provide natural points for biological interpretation and hypothesis generation. For instance, identifying which protein domains, regulatory modules, or cell clusters contribute most to a prediction can guide experimental validation and mechanistic investigation. This multi-scale interpretability aligns closely with how biologists conceptualise systems-level organisation.

Nevertheless, pooling and hierarchical methods introduce challenges. Biological hierarchies are often overlapping, context dependent, and incompletely characterised, whereas computational pooling typically enforces discrete and static cluster assignments. Over-aggressive pooling may discard biologically meaningful heterogeneity or obscure rare but critical subpopulations, such as low-frequency cell states or regulatory hubs. Consequently, effective hierarchical modelling requires careful alignment with biological knowledge, appropriate resolution selection, and validation against known structural or functional annotations.

In summary, pooling and hierarchical representation methods provide a principled framework for capturing the multi-scale organisation of biomolecular systems. By enabling graph neural networks to learn representations across hierarchical levels, these approaches support both scalable computation and biologically interpretable inference, bridging local molecular interactions and system-level biological function.

##### SortPooling

To enable CNNs to be better applied to graph-structured data, Zhang et al. [76] proposed a novel deep learning architecture that operates on arbitrary graphs. This architecture, termed Deep Graph Convolution Neural Network (DGCNN), contains three parts: (1) multiple graph convolution layers for neighbouring information aggregation, (2) a SortPooling layer to sort nodes in a consistent order for producing a graph representation, and (3) traditional convolutional and dense layers to perform classification.

For each vertex in the graph, the outputs of multiple graph convolution layers are concatenated horizontally as its final representation $\left[Z^{1},\ldots ,\;Z^{h}\;\right]$ with the size of $R^{1\times \sum_{1}^{h}c_{t}}$, where h represents the number of graph convolution layers, $c_{t}$ represents the number of feature channels in the $t^{th}$ layer.

The SortPooling layer is the main innovation of the model, which can learn from global graph topology and form a fixed-size input for a downstream traditional convolutional structure, acting as a bridge between graph convolution and traditional convolutional structure. It sorts the vertices based on the output. $Z^{h}$ of the last graph convolution layer. Vertices are arranged in a descending order according to the value of the last feature channel in $Z^{h}$. To unify the size of the tensors fed into the traditional convolutional structure, the SortPooling layer either truncates or extends vertices to match a unified number k across graphs.

Finally, the final representations of selected vertices are reshaped to a $k\left(\sum_{1}^{h}c_{t}\right)\times 1$ vector representing the graph and fed into the standard convolutional structure for prediction and classification.

Compared to PATCHY-SAN, the main difference is that DGCNN integrates the process of sorting vertices into the model, as the SortPooling layer allows for passing loss gradients to previous graph convolution layers.

##### DiffPool

In 2018, Rex Ying et al. [77] proposed a pooling method on GNNs: DiffPool, where he achieves cluster representation through the assignment matrix S, thereby generating the corresponding new node/cluster embedding Z:

(58) $$S^{\left(l\right)}=softmax\left({GNN}_{pool}\left(A^{\left(l\right)},X^{\left(l\right)}\right)\right)$$

(59) $$Z^{\left(l\right)}={GNN}_{embed}\left(A^{\left(l\right)},X^{\left(l\right)}\right),$$

where $S^{\left(l\right)}\in R^{n_{l}\times n_{l+1}}$, Each row in $S^{\left(l\right)}$ represents a node/cluster in row l, and each column represents the clusters in row l+1. Then, we use the obtained cluster assignment matrix and cluster embedding matrix to depool the feature matrix and adjacency matrix of the next layer. After n layers of learning, a graph representation that aggregates key information was ultimately obtained.

Since graph data is predominantly unsupervised, the processing of adjacency matrices becomes particularly crucial. The mincut method [78] proposes a novel loss function that optimises the classification of clusters under unsupervised conditions:

(60) $$L_{u}=L_{c}+L_{o},$$

(61) $$L_{c}=-\frac{Tr\left(S^{T}\overset{\sim }{A}S\right)}{Tr\left(S^{T}\overset{\sim }{D}S\right)},$$

(62) $$L_{o}={\left\|\frac{S^{⊤}S}{\|S^{⊤}S{\|}_{F}}-\frac{I_{K}}{\sqrt{K}}\right\|}_{F},$$

where $S=softmax(ReLU(XW_{1})W_{2})\in R^{N\times K}$, K denotes the target number of clusters; $S_{i}$ represents the probability that the i’th node belongs to each cluster. The cut loss $L_{c}$ encourages the pooling assignment matrix S to group together nodes that are strongly connected in the original graph. Maximising the trace ratio means maximising within-cluster connections relative to the overall volume. $L_{o\;}$ encourages each cluster to be distinct and evenly distributed.

##### SAGPool

In 2019, Junhyun Lee et al. [79] combined pooling with attention mechanisms: SAGPool, which introduces an attention score:

(63) $$Z=\sigma \left({\bar{D}}^{-1/2}\overset{\sim }{A}{\bar{D}}^{-1/2}X\Theta_{\mathrm{att}}\right),Z\in R^{N\times 1},$$

where $\Theta_{\mathrm{att}}$ It is the only learnable vector parameter. $Z\in R^{N\times 1}$ refers to each node’s attention score.

Given a pooling ratio k∈(0,1], the top kN nodes with the highest scores are selected. Their indices are denoted as:

(64) $$idx=top\text{-}rank\left(Z,\left\lceil kN\right\rceil \right),{\;Z}_{mask}=Z_{idx}$$

Subsequently, masking and subgraph extraction are performed:

(65) $$X_{out}=X_{idx,:}⊙Z_{mask},A_{out}=A_{idx,idx},$$

where ⊙denotes the row-wise broadcasted element-wise multiplication

**Figure 7:**
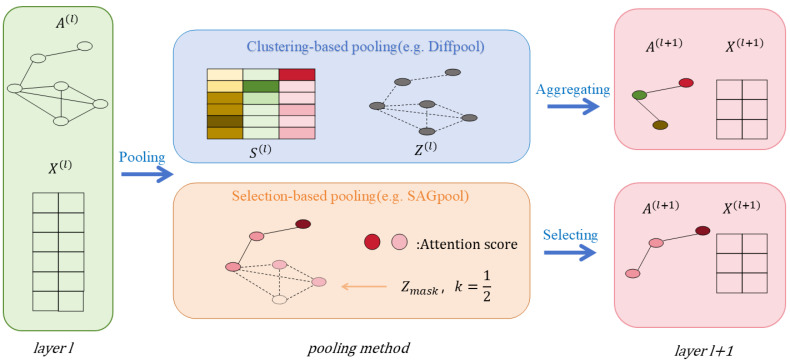
The difference between Diffpool and SAGpool.

This process results in a new, smaller-scale graph that preserves the most important nodes and edges. This step combines the topological term ${\overset{\sim }{D}}^{-1/2}\overset{\sim }{A}{\overset{\sim }{D}}^{-1/2}$ with the node features X to generate the attention scores. As a top-k selection method, SAGpool is independent of the number of input nodes, with a time complexity of O(|V|+|E|). This outperforms diffpool’s $O(kV^{2})$ approach and demonstrates significantly better performance on sparse graphs than diffpool. And Figure 7 shows the difference between the Diffpool and SAGpool methods.

#### 3.2.8. Decoupled Propagation

Decoupled propagation mechanisms arise from the recognition that, in many biological systems, information transformation and information diffusion are governed by distinct processes. Biologically, feature transformation often reflects intrinsic properties of molecular entities, such as sequence composition, structural motifs, or biochemical states. Additionally, information propagation reflects interaction-mediated influence across networks. Treating these processes as inseparable, as in tightly coupled message-passing architectures, can obscure biological interpretation and exacerbate noise sensitivity.

In graph neural networks, decoupled propagation separates feature transformation from neighbourhood aggregation. Typically, node features are first transformed through learnable functions independent of the graph structure, followed by a propagation or diffusion step that spreads information according to network connectivity. This design embodies the biological assumption that intrinsic molecular characteristics and relational influence can be modelled as complementary but distinct components. For example, a gene’s sequence-derived or epigenetically informed features can be learned independently, while regulatory or interaction networks determine how such features influence downstream genes or pathways.

Decoupled propagation offers several advantages for biological graph learning. By reducing repeated entanglement of feature mixing and propagation, these models mitigate over-smoothing, a phenomenon in which biologically distinct entities become indistinguishable after multiple message-passing layers. This is particularly important in large, sparse biological graphs, such as protein–protein interaction networks or single-cell similarity graphs, where excessive smoothing can erase rare but functionally critical signals. Decoupled designs also improve robustness to graph noise, as propagation depth and strength can be controlled without repeatedly reparameterising feature transformations.

From an interpretability standpoint, decoupled propagation facilitates clearer attribution of model behaviour. Separating intrinsic feature encoding from relational diffusion allows researchers to more readily assess whether predictions are driven by molecular properties, network context, or their interaction. This distinction aligns with biological reasoning, where mechanistic hypotheses often differentiate between cell-intrinsic factors and extrinsic regulatory influences. For clinicians and experimental biologists, such separation can support more transparent interpretation and experimental validation.

Nevertheless, decoupling also introduces trade-offs. Overly simplistic propagation schemes may underutilise rich local interaction patterns, while excessive reliance on precomputed features may limit the model’s ability to adapt representations to specific biological contexts. Moreover, decoupled approaches still depend on the quality of the underlying graph; erroneous or biassed connectivity can propagate misleading signals even when feature learning is well regularised. Effective application therefore requires careful calibration of propagation depth, incorporation of biological priors, and validation against known interaction structures.

In summary, decoupled propagation provides a biologically motivated architectural strategy that enhances robustness, scalability, and interpretability in graph neural networks. By explicitly separating feature transformation from information diffusion, these methods align more closely with the modular organisation of biological systems and offer a flexible framework for learning on large, noisy, and heterogeneous biomolecular graphs.

##### Personalised Propagation of Neural Predictions (PPNP)

Since graph convolutional networks rely on the neighbourhood aggregation scheme to aggregate neighbour information, nodes become more and more dependent on their neighbours with the increased number of layers. High aggregation degrees can lead to the over-smoothing problem [67,80,81,82]. In addition, leveraging information from a large neighbourhood requires increasing the depth of the neural network. To solve these two issues, Klicpera et al. [83] recognised the relationship between GCNs and PageRank [84] and proposed a novel propagation scheme based on personalised PageRank. The proposed propagation scheme, termed personalised propagation of neural predictions (PPNP), allows models to preserve locality and leverage the information from a larger neighbourhood in propagation without causing over-smoothing.

This propagation scheme separates the neural network from neighbourhood information aggregation. It, therefore, can be combined with any state-of-the-art neural networks. PPNP-based model first uses a convolutional neural network to generate node representations (with the same size as the label) for each node separately based on its own node features. The adapted personalised PageRank scheme is finally applied to aggregate neighbourhood information based on learned node representations for final predictions. The personalised PageRank for node x specifies the influence score of it on node y, and it can be obtained as follows,

(66) $$\pi_{ppr}\left(i_{x}\right)=\;\alpha {\left(I-\left(1-\alpha \right)\hat{\tilde{A}}\right)}^{-1}i_{x},$$

where $i_{x}$ is a one-hot teleport vector for node x, $\alpha \in \left(0,\;\left.1\right]\right.$ is the teleport probability. The introduction of teleport vector and teleport probability allows for preserving locality. The fully personalised PageRank matrix can be obtained by substituting the teleport vector $i_{x}$ with the identity matrix I as follows,

(67) $$\Pi_{ppr}=\;\alpha {\left(I-\left(1-\alpha \right)\hat{\tilde{A}}\right)}^{-1},$$

whose element specifies the influence score of a node on the rest. The PPNP-based model can be formulated as:

(68) $$Z_{PPNP}=softmax\left(\Pi_{ppr}H\right),\;H_{i,:}=f_{\theta }\left(X_{i,:}\right),$$

where X is the node feature matrix, $f_{\theta }$ is the neural network, $H\in R^{n\times c}$ is the learned node representation matrix for n nodes with the representation size of c, and $Z_{PPNP}$ denotes the predictions.

Through the decoupling feature transformation from neighbourhood aggregation, PPNP breaks the fixed relationship between neighbourhood size and the depth of neural networks. This procedure solves the limited range problem of graph convolutional networks, allowing networks to aggregate information from a larger neighbourhood without increasing the number of layers in the neural network.

### 3.3. Optimisation Techniques for Large-Scale Biological Graphs

Optimisation strategies in graph neural networks are often presented as generic solutions to computational scalability or training efficiency. In biological applications, however, optimisation choices are closely tied to the characteristics and limitations of experimental data. Biological graphs are typically large, sparse, noisy, and incomplete, reflecting both biological variability and technical constraints of high-throughput measurements. As a result, optimisation techniques play a critical role not only in enabling scalable learning but also in stabilising training, mitigating noise propagation, and preserving biologically meaningful signals.

In this section, we frame optimisation methods as biologically motivated design choices rather than purely algorithmic refinements. We discuss how sampling strategies, loss function design, and training protocols address challenges specific to biomolecular data, including class imbalance, uncertainty, data sparsity, and limited labelled samples. This perspective highlights how advances in optimisation directly enable the application of GNNs to realistic biological and clinical datasets, thereby supporting robust inference and translational research.

#### 3.3.1. Random Sampling

From a biological perspective, sampling strategies help control the propagation of experimental noise and spurious interactions in large, sparsely validated biological graphs, where exhaustive neighbourhood aggregation may amplify measurement artefacts. During training, graph neural networks encounter information propagation challenges. The most common issue is over-smoothing, which causes node information to converge excessively. This is why graph neural networks typically have only one or two layers. However, too few layers can lead to insufficient information aggregation. Therefore, Operations on graphs during training are crucial. Unlike graph structure optimisation, random sampling is temporary and does not alter the original structure.

In traditional neural networks, Dropout serves as an effective regularisation method [85]. Randomly dropping hidden units and their connections during training reduces neuron co-adaptation, lowering overfitting risk and improving generalisation. In GNNs, a similar Dropout operation applied to the feature vector X remains applicable. However, due to the unique nature of graph structures, random sampling/dropping can be performed at the structural level (nodes or edges) on graph data.

In graph neural networks (GNNs), random sampling methods such as Dropout, DropEdge, and DropNode help prevent overfitting and improve generalisation, but they also introduce information loss. Because nodes in a graph are highly interdependent, once certain nodes or edges are randomly dropped, their features vanish, and their neighbours’ message aggregation becomes incomplete, breaking the continuity of information flow. As the network depth increases, this loss is amplified layer by layer, leading to representation degradation and over-smoothing, which severely weakens deep GNN performance. To address this issue, an information compensation mechanism is introduced. This mechanism employs a complementary mask to identify dropped nodes and reintroduces their original features from shallower layers, thereby preserving message flow without altering the random sampling strategy. The procedure consists of three main steps: (1) randomly generating a node mask. $b^{\left(l\right)}$; (2) using its complementary mask ${1-b}^{\left(i\right)}$ to extract the dropped nodes’ features from a shallower representation $H^{\left(i\right)}$; and (3) adding them back to the current layer output $H^{\left(l\right)}$ for compensation. This process effectively alleviates over-smoothing, maintains inter-layer representation diversity, and enhances the stability and robustness of deep GNNs [86].

**Figure 8:**
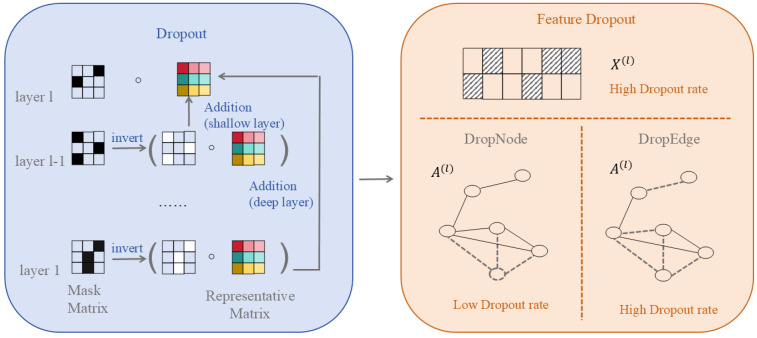
The Evolution of Random Sampling Methods.

Unlike structured learning approaches such as GSL, methods like DropEdge do not alter the original graph’s semantics. Instead, they introduce noise through random perturbations, serving a function analogous to “data augmentation” in the image domain. To enhance model robustness and generalisation capability, temporary random perturbations may be applied to the graph structure during training without altering the original graph, and the changes are shown in Figure 8:

DropEdge: Randomly removes a proportion of edges per training iteration, mitigating over-smoothing in deep GCNs while serving a regularisation function.

DropNode: Generates subgraphs via Bernoulli sampling or random walks to reduce inter-node dependencies, particularly suited for large-scale graphs (e.g., whole-genome regulatory networks).

Such strategies may be regarded as graph data augmentation, proving especially effective for biomedical tasks with limited samples.

##### DropNode

In mature GCN models, such as GraphSAGE (Hamilton et al., 2017 [60]), FastGCN (Chen et al., 2018 [87]), the process of randomly sampling nodes had already emerged. In 2021, Tien Huu Do et al. [88] officially named a DropNode sampling approach. The fundamental idea is to randomly sample subgraphs from the input graph during each training iteration. If the nodes connected by an edge are retained, the edge is also retained; otherwise, the edge is discarded. The specific sampling procedure is as follows:

(69) $$r={\left(r_{1},\ldots ,r_{N}\right)}^{T}\;\;\;\;\;\;\;\;\;\;\;\;H^{\left(l+1\right)},ID^{\left(l\right)}=SELECT\left(H^{\left(l\right)},r\right),$$

where the r vector determines which nodes should be retained, and the corresponding adjacency matrix must be modified accordingly:

(70) $${\overset{\sim }{A}}^{\left(l+1\right)}=SELECT\left({\overset{\sim }{A}}^{\left(l\right)},r\right)$$

Regarding subgraph selection, two approaches exist: Bernoulli sampling and random walk sampling. Table 5 lists the differences between these methods:

During training, the model does not observe all nodes. Consequently, the model utilises information from all nodes in the graph. This reduces the risk of the model memorising training samples, thereby preventing overfitting. Additionally, the model is trained using multiple transformed versions of the original graph, which can be viewed as a form of data augmentation. On the other hand, the DropNode method reduces connectivity between nodes in the graph. Lower connectivity helps mitigate the smoothing phenomenon in node representations as GCNN models become deeper.

##### DropEdge

Corresponding to DropNode, in 2020, Yu Rong et al. [89] proposed DropEdge method. DropEdge is a method that randomly removes edges during training. At each epoch, DropEdge randomly discards a certain proportion of edges from the input graph. Formally, it randomly sets $V_{p}$ nodes in the adjacency matrix A to zero, where V is the total number of edges in the graph and p is the drop rate.

If the adjacency matrix after dropping is denoted as $A_{drop}$, then its relationship with A is:

(71) $$A_{drop}=A-A^{\prime },$$

where A is a sparse matrix expanded from a randomly selected subset of size Vp from the original edge set.

DropEdge simultaneously prevents overfitting and oversmoothing:

Overfitting: DropEdge ensures that training aggregation relies only on a random subset of neighbours, not the entire set. Furthermore, if edges are discarded with probability p, the expected value of neighbour aggregation is multiplied by p. After weight normalisation, this factor cancels out, meaning DropEdge does not alter the expected value of neighbour aggregation.

Oversmoothing: Based on the definition of ε-smoothing, the paper introduces the relaxed ε-smoothing layer to describe the convergence speed:

(72) l^M,ε=logε/dMXlogsλ,

where s is the supremum of the singular values of all convolutional filters, and λ is the second largest eigenvalue of A^. By randomly dropping edges, DropEdge increases λ, which makes l^M,ε larger and slows down the convergence of node representations, thus mitigating over-smoothing. At the same time, removing edges enlarges the dimension of the converging subspace, which reduces information loss and helps preserve more discriminative features.

The Table 6 compares four graph production methods.

#### 3.3.2. Loss Function

In bioinformatics tasks, loss function design reflects biological priorities, such as handling severe class imbalance, accounting for uncertain or weak labels, and balancing predictive accuracy with interpretability.

Regularisation is a technique commonly used in machine learning and data analysis. Its core principle involves leveraging similarities or relationships between data points (typically represented as graph structures) to guide the model learning process. This enables the model to better preserve the inherent local or global structural information within the data, thereby enhancing learning effectiveness, improving generalisation capabilities, and increasing the interpretability of results. Similarly, graph regularisation is typically achieved by incorporating a graph-based regularisation term into the objective function. The most classic form of graph regularisation is Laplacian regularisation, where the added term is:

(73) $$L_{reg}=\frac{1}{2}\sum_{i,j}A_{ij}\|f(x_{i})-f(x_{j}){\|}^{2}=Tr\left(V^{⊤}LV\right),$$

where V is the feature representation matrix for all nodes, and L is the graph’s Laplacian matrix.

In 2011, D. Cai et al. [90] introduced graph regularisation into non-negative matrix factorisation to address a limitation of standard NMF, which performs low-dimensional decomposition solely in Euclidean space while neglecting the inherent neighbourhood geometric relationships within graph data. Consequently, GNMF (Graph-Regularised Non-Negative Matrix Factorisation) was proposed, combining graph regularisation with non-negative matrix factorisation:

(74) $$O_{1}=\|X-UV^{T}{\|}^{2}+\lambda Tr(V^{T}LV)$$

where $\lambda Tr(V^{T}LV)$ represents the graph regularisation term, which penalises points that are connected in the graph but exhibit excessive divergence in their low-dimensional representations. This mechanism enables the data representation to balance both “modular interpretability” and “manifold geometric preservation.”

In 2021, Yang H. et al. [91] introduced Propagation Regularisation (P-reg), whose core idea is to align each node’s current prediction Z with its prediction after one round of propagation among neighbours A^Z:

(75) LP−reg=1NφZ,A^Z.

There are 3 φ functions to choose:

(76) φSEZ,A^Z=12∑i=1N‖(A^Z)i−Zi‖22.

(77) φCEZ,A^Z=−∑i=1N∑j=1CPijlogQij.

(78) φKLZ,A^Z=∑i=1N∑j=1CPijlogPijQij.

Since mainstream GNN convolutions inherently incorporate first-order Laplacian information, adding Laplacian regularisation to GCN/GAT typically yields minimal gains. P-reg is equivalent to “squared Laplacian regularisation”: its corresponding regularisation matrix belongs to another class of operators within the spectral regularisation framework, thus offering a distinct supervision mechanism from traditional Laplacian regularisation. Simultaneously, as it performs soft target alignment between nodes and their neighbourhoods, it can inject additional categorical information to all nodes (including unlabelled ones).

(79) $$L_{lap}=\sum_{\left(i,j\right)\in E}\left\|Z_{i}-Z_{j}{\|}^{2}=Tr(Z^{⊤}LZ\right),$$

(80) $$L_{P-SE}=\frac{1}{2N}\left\langle Z,{\overset{\sim }{\Delta }}^{T}\overset{\sim }{\Delta }Z\right\rangle ,$$

### 3.4. Recent Trends in GNNs Development Relevant to Bioinformatics

Biological data are increasingly represented as complex graphs—ranging from molecular interaction networks and protein structures to brain connectomes, patient trajectories, and single-cell multi-omics. Modelling these irregular, high-dimensional structures has therefore become central to modern bioinformatics. Recent advances in graph neural networks (GNNs) have enabled deeper integration of topological, molecular, and temporal information, driving substantial progress in biological prediction, discovery, and generative modelling.

To address the scarcity of high-quality labels in biological datasets, semi-supervised and self-supervised learning have emerged as essential strategies. Semi-supervised methods propagate sparse labels over similarity graphs, while self-supervised approaches, such as generative, auxiliary, contrastive, or hybrid methods, derive supervision directly from graph structure and node features to enhance robustness and generalisation in biological tasks.

Many biological processes are dynamic, motivating the development of “spatio-temporal-based GNNs”, which couple graph convolutions with temporal models to capture evolving patterns in brain activity, disease progression, and cellular development. In parallel, “GNN-based diffusion model” merges probabilistic diffusion models with graph encoders to generate molecular structures, model gene expression trajectories, and simulate cell-state transitions.

Given the limited annotations typical in bioinformatics, contrastive learning has become particularly influential. By contrasting augmented or cross-modal views of molecules, proteins, or cells, these methods learn invariant, biologically meaningful representations that enhance downstream performance. “Transformer-enhanced GNNs” integrate global attention mechanisms with graph structure, enabling more effective modelling of long-range dependencies in protein sequences, molecular graphs, and heterogeneous biomedical networks.

Together, these methodological advances are reshaping computational biology by providing powerful, scalable frameworks for prediction, integration, and generation across diverse biological systems. The following sections outline their core principles and highlight their growing impact in bioinformatics research.

#### 3.4.1. Semi-Supervised Learning

Semi-supervised learning bridges the gap between supervised and unsupervised paradigms by exploiting both labelled and unlabelled data. Since graph data typically contains a large number of nodes and involves substantial data volumes, labelled examples are often scarce and costly to obtain, while unlabelled data are abundant, semi-supervised methods leverage the underlying structure of data distributions to propagate label information. This approach improves generalisation, reduces annotation requirements, and has been widely applied in text classification, image recognition, and graph-structured data analysis.

In 2003, Xiaojin Zhu et al. [92] first proposed a method for performing semi-supervised learning on graph data. They represent labelled and unlabelled samples as nodes in a weighted graph, where edge weights characterise similarity between instances. A Gaussian random field is defined on this graph, whose mean satisfies the harmonic function condition—meaning the value of an unlabelled point equals the weighted average of its neighbours:

(81) $$E\left(f\right)=\frac{1}{2}\sum_{i,j}w_{ij}(f_{i}-f_{j}{)}^{2}⟹\left(\partial E/\partial f_{i}=0\right)\Rightarrow f_{i}=\frac{\sum_{j}w_{ij}f_{j}}{\sum_{j}w_{ij}}(\mathrm{for}\;\mathrm{unlabeled}\;i),$$

where f means the function value (or label assignment) at node i, E means the energy function measuring the smoothness of the graph. This enables an efficient solution via matrix methods or belief propagation.

After that, Mikhail Belkin et al. [93] have proposed Manifold Regularisation. By precisely constructing dual regularisation terms to adjust the relationship between parameters, the goal of effectively utilising labelled data and graph data is achieved. Specifically, the formula is as follows:

(82) $$f^{*}=arg{min}_{f\in H_{K}}\frac{1}{l}\sum_{i=1}^{l}V(x_{i},y_{i},f)+\gamma_{A}\|f{\|}_{K}^{2}+\gamma_{I}\|f{\|}_{I}^{2},$$

where the first term represents labelled data, ensuring the model accurately fits labelled information; the second term is an ambient regularisation term preventing overfitting; and the third term is an intrinsic regularisation term characterising the smoothness of the function on the data manifold, which depends on the geometric structure of all data. The third regularisation term is computed as follows:

(83) $$\|f{\|}_{I}^{2}=\int_{M}\left\|\nabla_{M}f(x){\|}^{2}dP_{X}(x\right).$$

Here, $\|f{\|}_{I}^{2}$ denotes the intrinsic regularity term of the function f on the manifold M, $\nabla_{M}f(x)$ represents the gradient of f on the manifold, and $P_{X}$ is the marginal distribution of the input data. This term measures the smoothness of the function f on the data manifold.

This approach incorporates unlabelled samples through similarity graphs, forcing the function to smooth between neighbouring samples and thereby leveraging unlabelled data for learning.

In 2017, Kipf, T. N. et al. [49] directly incorporated the graph structure into the neural network f(X, A), supervising only labelled nodes while allowing gradients to propagate along the graph. This enables simultaneous learning of representations for both labelled and unlabelled nodes, directly embedding semi-supervised learning into GCNs. Thus became the mainstream formula for reasoning.

(84) A^=D~−1/2A¯D~−1/2

(85) Z=f(X,A)=softmax(A^ReLU(A^XW(0))W(1))

In 2019, Jiang et al. [94] proposed a dynamic learning approach that simultaneously learns graphs and convolutions within the network, enabling the graph structure to adapt to the task and thereby enhancing semi-supervised performance.

It learned $S_{ij}=g(i,j)$ to dynamically measure the importance of nodes in relationships.

(86) $$S_{ij}=\frac{exp(ReLU(a^{⊤}|x_{i}-x_{j}|))}{\sum_{j}exp(ReLU(a^{⊤}|x_{i}-x_{j}|))}$$

And utilises loss functions is:

(87) $$L_{GL}=\sum_{i,j}\frac{1}{2}\|x_{i}-x_{j}{\|}^{2}S_{ij}+\gamma \|S{\|}_{F}^{2}$$

Subsequently, the fixed matrix A in the GCN is entirely replaced with an adaptive S, yielding the total loss:

(88) $$L_{\mathrm{Semi}\text{-}\mathrm{GLCN}}=L_{\mathrm{Semi}\text{-}\mathrm{GCN}}+\lambda L_{\mathrm{GL}}$$

#### 3.4.2. Self-Supervised Learning

In the context of graph learning, the fundamental distinction between supervised/semi-supervised learning and self-supervised learning/weakly supervised learning (WSL, here, weak supervision refers to scenarios where labels are available only at the slide or patient level, rather than at the level of individual cells or tissue regions.) lies in the “source of supervision signals.” The former relies on manually annotated downstream task labels to train models, whereas self-supervised learning (SSL) directly constructs supervision signals from the data itself through pretext tasks, eliminating the need for human annotation. In biological settings, pretext tasks are most effective when they correspond to experimentally interpretable perturbations rather than abstract graph augmentations. The purely supervised paradigm suffers from three prominent issues: high annotation costs, susceptibility to overfitting, and weak robustness. In contrast, SSL learns more informative representations from unlabelled data through pretext tasks like structure recovery, contrastive consistency, and attribute mining. It typically delivers superior performance, stronger generalisation, and higher robustness across various downstream tasks, achieving significant success in computer vision and natural language processing [95].

In terms of applicability, SSL is well-suited for graph data scenarios with sparse or costly labels, such as e-commerce recommendations, transportation networks, chemical molecules, and knowledge graphs—all of which involve widespread graph structures. Furthermore, SSL on graphs must handle non-Euclidean, irregular structures and inherent node correlations. This necessitates that pre-defined tasks simultaneously focus on both topological and node features, rather than merely performing local recovery or consistency transformations on regular grids (e.g., images/text).

In 2020, Qikui Zhu et al. [96] proposed generative SSL, which employs two approaches: Randomly Removing Links (RRL) and Randomly Covering Features (RCF), enabling GCNs to learn node representations directly from the graph data itself. Both self-supervised tasks are implemented through a link prediction module. This module aims to predict whether an edge exists between two nodes, expressed as:

(89) A^=sigmoid(Hi(Hi)T),

here $H^{i}$ represents the node representation learned by the GCN layer, and A^ denotes the reconstructed adjacency matrix.

During training, since the adjacency matrix A is sparse, weighted cross-entropy is used as the loss function, defined as:

(90) LL=−∑iW(Ailog(A^i)+(1−Ai)log(1−A^i)),

where *i* is the index set of the adjacency matrix, W is the positive sample weight, equal to the ratio of the number of non-connected edges to the number of connected edges in the adjacency matrix.

In 2019, Petar Veličković et al. [97] proposed contrast SSL, which constructs contrast maps through corruption operations, specifically by preserving the adjacency matrix (maintaining topological structure) while randomly scrambling the order of node features (disrupting local semantic correspondences):

(91) $$\left(\overset{\sim }{X},\overset{\sim }{A})=C(X,A)=(X_{\mathrm{shuffled}},A\right)$$

To distinguish positive and negative samples, we employ a Discriminator:

(92) $$D\left(h,s\right)=\sigma \left(h^{⊤}Ws\right)$$

where $H={\left[h_{1},h_{2},\ldots ,h_{N}\right]}^{⊤}\in R^{N\times F}$, and *W* is a learnable parameter matrix.is a node embedding matrix. $h_{i}$ represents the low-dimensional representation of node i, and s denotes the graph representation obtained by applying an aggregation function to the node embedding matrix.

(93) $$L=\frac{1}{N}\sum_{i=1}^{N}logD(h_{i},s)+\frac{1}{N}\sum_{i=1}^{N}log(1-D({\overset{\sim }{h}}_{i},s))$$

Existing approaches can be broadly categorised according to their training objectives, including generation-based, property-driven, contrastive, and hybrid methods, forming a unified framework for advancing graph representation learning [98]. And here is a detailed comparison of the four GCNs in Table 7.

#### 3.4.3. Spatio-Temporal-Related GNNs

Traditional GNNs assume static, unchanging graph structures, making it difficult to capture the temporal evolution characteristics prevalent in biological systems. To overcome this limitation, Spatio-Temporal GNNs couple graph convolutions with time series modelling (such as RNNs and Transformers), simultaneously capturing spatial dependencies and temporal dynamics.

Specifically, in functional magnetic resonance imaging (fMRI), functional connectivity (edges) between brain regions (nodes) dynamically alters with cognitive tasks or pathological states; spatio-temporal GNNs can model these time-varying brain networks for early prediction of Alzheimer’s disease or epilepsy. Similarly, in electronic health record (EHR) analysis, patient visit events can be structured as disease–symptom–medication heterogeneous graphs evolving over time, where spatio-temporal models capture disease progression trajectories and treatment response patterns. Moreover, within single-cell multi-omics time series, the topological structure of cellular state graphs alters during development or perturbations, and spatio-temporal GNNs can reveal the dynamic behaviour of key regulatory nodes [99]. Spatio-temporal graph convolutions not only extend GNNs’ modelling capabilities for dynamic biological processes but also provide a novel computational paradigm for understanding the temporal mechanisms of complex diseases and enabling personalised prognostic assessment.

Methodologically, temporal information is typically incorporated through two main strategies. One approach integrates spatiotemporal similarity directly during graph construction, connecting nodes based on both spatial proximity and temporal continuity. The other approach models spatial (or logical) and temporal relationships separately to derive static and dynamic representations, which are then fused into a unified embedding. Together, these strategies offer principled ways to encode temporal evolution into graph structures and make spatio-temporal architectures particularly suitable for biomedical dynamic scenarios.

**Figure 9:**
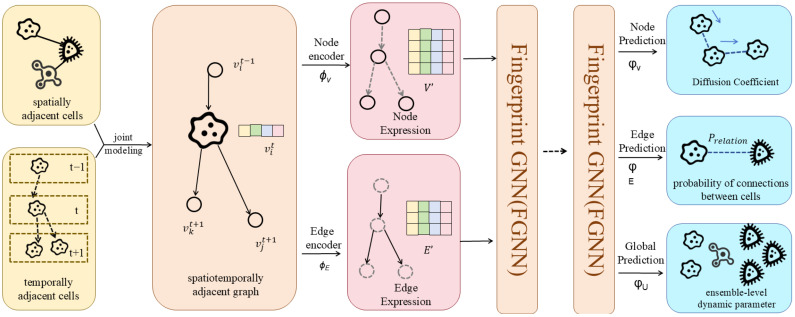
MAGIK framework.

For spatiotemporal sequence fusion, Jesús Pineda et al. [100] proposed the MAGIK framework, designed to model the motion of cells, organelles, and individual molecules across multiple spatial and temporal scales. This method performs fusion at the graph construction level through three major steps, the details of which are shown in Figure 9:

(1) Graph Construction: Nodes (V) represent detected biological entities, while edges (E) connect detections that are adjacent in space and time. Each node encodes biological descriptors such as mass, morphology, and intensity-related features, whereas edges encode relational features, e.g., the Euclidean distance between cell centroids. Spatially and temporally associated detections are connected, typically constrained within two frames (temporal) and within 20% of the full field of view (spatial).

(2) Local Spatiotemporal Neighbourhood Aggregation: For each edge, a feature vector $e_{ij}^{\prime \prime }$ is formed by concatenating the source node features, target node features, and edge attributes. These edge features are then weighted by spatial distance $w_{ij}$ to define a *learnable local receptive field*, enabling adaptive modelling of heterogeneous motion scales. The weighted edge features are aggregated into the target node and concatenated with its current state, followed by a linear transformation to obtain the node’s updated local representation ${\tilde{h}}_{i}$.

(94) $$w_{ij}=exp(-{\left(\frac{d_{ij}^{2}}{2\sigma^{2}}\right)}^{\beta })$$

(95) $${\overset{\sim }{h}}_{i}=W_{\overset{\sim }{H}}[v_{i}^{\prime },\sum_{j\in N_{i}}w_{ij}e_{ij}^{\prime \prime }]$$

(3) Global Spatiotemporal Aggregation: A gated multi-head self-attention mechanism is employed to update the node hidden states. The gating vector $G^{\left(z\right)}$, generated through a sigmoid activation, regulates the contribution of each attention head. This design enables the model to cluster attention heads according to spatiotemporal similarity, thereby extending the temporal receptive field from the local neighbourhood to the global trajectory level.

(96) $$\begin{array}{cc} V\prime \prime \left(z\right)\; & =\;{attn}^{\left(z\right)}\left(H\right)\\ & =\;G^{\left(z\right)}⊙\left(softmax\left(\frac{1}{\sqrt{c}}Q^{\left(z\right)}{K^{\left(z\right)}}^{⊤}\right)P^{\left(z\right)}\right) \end{array}$$

In 2025, Eleni Giovanoudi [101] et al. proposed another strategy for integrating GNNs with spatiotemporal sequence data. Their framework processes spatial and temporal information through distinct modules and fuses the resulting embeddings via a linear layer to produce a unified representation. This approach was applied to gene co-expression prediction, significantly enhancing the ability to uncover dynamic patterns in biological time-series data. Moreover, it provided interpretable gene-module delineations that can support disease mechanism analysis, early diagnosis, drug response prediction, and cancer subtype identification.

Specifically, for static data, the model first constructs a weighted gene correlation graph $G_{G}$, on which a multi-layer Graph Convolutional Network is applied to extract global co-expression features, yielding the global gene embeddings $V_{G}$. A linear decoder then reconstructs the expression matrix to compute the reconstruction loss $L_{rec}^{G}$.

For dynamic (temporal) data, a set of Euclidean distance-based nearest-neighbour graphs $\left\{G_{D}^{1},\ldots ,G_{D}^{t-1}\right\}$ is constructed between each pair of consecutive time points. Each dynamic graph is encoded using a Graph Attention Network (GAT), augmented with a custom Temporal Attention (TA) module. The TA layer computes an attention mask $m_{k-1}$ for the previous time step, which is element-wise multiplied with the current graph’s node features before being fed into the GAT layer. This design enables explicit temporal dependency propagation, allowing information from the preceding time step to directly influence the representation of the subsequent one.

(97) $$\begin{array}{rr} m_{k-1}^{\left(y\right)} & =TA\left(H_{k-1}^{D\left(y\right)}\right)\\ {\hat{H}}_{k}^{D\left(y\right)} & =H_{k}^{D\left(y\right)}⊙m_{k-1}^{\left(y\right)}\\ H_{k}^{D\left(y+1\right)} & =GAT\left({\hat{H}}_{k}^{D\left(y\right)}\right) \end{array}$$

The encoded representations are independently decoded through linear layers to obtain the reconstruction loss $L_{rec}^{D}$ and the temporal consistency loss $L_{con}$. The final objective function is defined as follows:

(98) $$L=L_{rec}^{G}+L_{rec}^{D}+\lambda L_{con}$$

**Figure 10:**
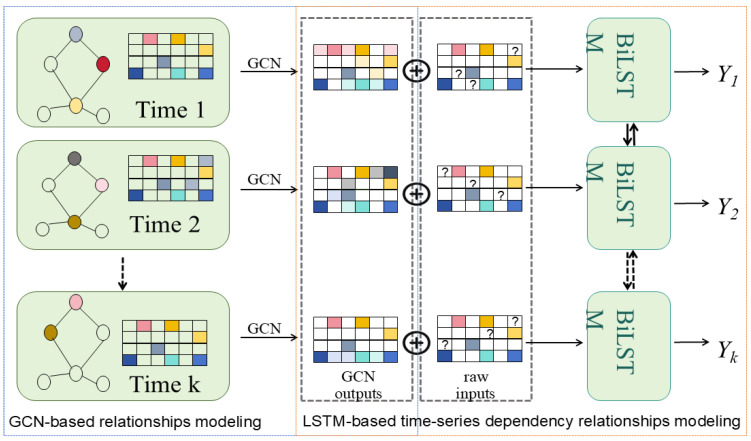
The pipeline of NetOIF.

In addition to parallel architectures that place the GNN and temporal processing modules side by side, there also exist serial integration approaches. In 2022, Xiaoli Jing et al. introduced NetOIF [102], as shown in Figure 10, which incorporates an LSTM into the GNN framework to jointly perform missing value imputation and future time-point prediction for temporal omics data.

Specifically, during the graph construction phase, each time point t corresponds to a graph $G_{t}=(V,E,X_{t})$, where nodes represent genes or proteins and edges denote biological network relationships (with possible missing node features). The GCN module utilises the adjacency matrix A to propagate information among connected nodes.

(99) $$Z_{t}=\mathrm{ReLU}({\tilde{D}}^{-1/2}(\;A+I\;){\tilde{D}}^{-1/2}X_{t}W)$$

The output $Z_{t}$ represents the topology-refined node features, which are used to infer missing values and restore the natural tendency of similar features among neighbouring nodes. Subsequently, $Z_{t}$ is fed into a bidirectional LSTM (BiLSTM) network, where the GCN output $Z_{t}$ and the original input $X_{t}$ are weighted and fused to capture both spatial and temporal dependencies.

(100) $$X_{t}^{\prime }=\alpha X_{t}+(1-\alpha )Z_{t}$$

Subsequently, the sequence $\left\{X_{1}^{\prime },X_{2}^{\prime },\ldots ,X_{k}^{\prime }\right\}$ is fed into a bidirectional LSTM (BiLSTM) to model temporal dependencies across consecutive time points.

(101) $$Y_{t+1}=\vec{h_{t}}+\overset{\leftarrow }{h_{t}}$$

Here, $\vec{h_{t}}$ and $h_{t}$ denote the hidden states of the BiLSTM layers in the forward and backward directions, respectively. 

This model is capable of performing two tasks simultaneously:

Data Imputation: Utilising the graph data from the previous k−1 time points to complete the missing values at time k.

Data Forecasting: Employing a sliding window strategy to predict the omics data at the future time point k+1.

#### 3.4.4. GNN-Based Diffusion Model

The diffusion model is a generative approach that produces images by reversing a noising process [103]: it “recovers” plausible data from simple, unstructured noise. Its operation consists of two phases: the forward process progressively corrupts training data (e.g., images) by adding Gaussian noise in multiple steps, while the reverse process trains a neural network (such as U-Net) to iteratively denoise pure noise, ultimately reconstructing novel data samples. Initially applied in AIGC, diffusion models are now widely integrated with graph neural networks (GNNs). The former provides powerful, sequential generation capability to yield high-quality and diverse samples, while the latter specialises in handling non-Euclidean data (e.g., graphs, point clouds, molecular structures) and effectively captures topological relationships and dependencies between nodes. As a result, Diffusion-GNN models show broad application prospects in scenarios involving the generation or processing of data with inherent relational structures, such as molecular graph generation, single-cell sequencing, and drug design.

**Figure 11:**
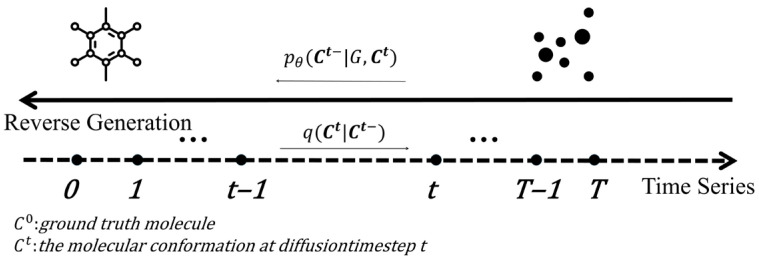
Simple architecture of GNN-based Diffusion Model.

One major application of diffusion-type GNNs is in the generation of molecular graphs. The basic method is shown in Figure 11. Generally, processing such models falls into two categories: (1) de novo generation of molecular graphs from basic structures, evaluated on datasets such as QM9, MOSES, and GuacaMol using metrics like validity/uniqueness/novelty, NLL, and FCD; and (2) property-conditioned molecular generation, where molecules are designed to satisfy specific graph-level attributes.

In 2022, Minkai Xu et al. [104] first introduced a GNN-based diffusion model for molecular graph generation. The core idea involves learning the reverse diffusion process to recover molecular geometric distributions from noise. Here, the diffusion model defines the noising and denoising processes of molecular conformations—gradually perturbing real conformations into Gaussian noise and then reconstructing the structure via a learned reverse Markov chain. The Graph Field Network (GFN) acts as the denoising network, predicting refined molecular representations at each timestep based on the molecular graph and the current noisy conformation.

In 2023, Clément Vignac [105] proposed DiGress, which replaces continuous Gaussian noise with discrete Markov “graph edits” (e.g., edge additions/deletions), maintaining sparsity and structural interpretability. The model also incorporates a classifier to guide the generation toward desired molecular properties. In 2024, Han Huang et al. [106] introduced JODO (Joint 2D–3D Diffusion Model), enabling simultaneous modelling of 2D topology and 3D geometry in molecular generation. This approach combines graph diffusion with equivariant coordinate updates, synchronously learning bond connectivity and spatial coordinate distributions during denoising. Later in 2025, Donghan Wang et al. [107] proposed GADIFF, which enhances GNN representations by integrating Graph Isomorphism Networks (GIN) to capture local information from subgraphs with different edge types (e.g., atomic bonds, angle/torsional interactions, long-range interactions), and employs multi-head self-attention (MSA) as a noise-attention mechanism to capture global molecular information.

In 2024, YuChen Liu et al. [108] extended Diffusion-GNN to single-cell RNA sequencing (scRNA-seq) analysis. By constructing cell–cell interaction graphs, the method models gene expression propagation and state transitions among cells. It simulates the dynamic equilibrium of expression diffusion across cellular neighbourhoods using a learnable attention mechanism to represent “diffusion rates” between cells. This offers a unified graph-learning framework for clustering, lineage tracing, and cell fate inference, providing an interpretable and biologically consistent approach for scRNA-seq data analysis.

#### 3.4.5. GNN with Contrastive Learning

In bioinformatics, self-supervised learning methods based on contrastive learning prevail most commonly. Mainstream contrastive learning approaches primarily involve contrasting two augmented graphs [109]. These methods are constructed based on the similarity of node features across different augmented views and consist of three steps in total.

Step 1: Graph Data Augmentation

This step generates diverse augmented views of the input graph, injecting prior knowledge of data distribution to enable the model to learn invariance to specific perturbations. Primary augmentation techniques include node dropping, edge perturbation, random sampling, etc. Two differently augmented graphs, denoted as and, are selected as source data for contrastive learning.

Step 2: GNN-based Encoder

This step extracts graph-level representations by mapping the augmented graphs to low-dimensional vectors. Specifically, z1=fG^1,z2=fG^2 where $f\left(\cdot \right)$ denotes the encoder function. These vectors are then projected into the contrastive learning space.

Step 3: Contrastive Loss Function

The loss function maximises consistency between positive pairs and discriminates against negative pairs. The normalised temperature-scaled cross-entropy loss (NT-Xent) is adopted:

(102) $$l_{n}=-log\frac{exp(sim(z_{n,i},z_{n,j})/\tau )}{\sum_{n^{\prime }=1,n^{\prime }\neq n}^{N}exp(sim(z_{n,i},z_{n^{\prime },j})/\tau )},$$

in which,

- sim$\left(\cdot \right)$ is the cosine similarity function;
- τ is a temperature hyperparameter controlling the sharpness of the distribution;
- The denominator aggregates augmented views of other graphs within the same batch as negative pairs. For a batch size of N,2N augmented graphs are generated, and the loss is computed as the average over all positive pairs.

In bioinformatics, high-quality labelled data are scarce because data labelling relies heavily on extensive biological experiments. Contrastive learning reduces dependence on labelled data, learns representations invariant to data perturbations, enhances model robustness on unseen data, mitigates overfitting, and facilitates transfer to downstream tasks—thus, it is widely adopted.

In 2023, Gu et al. [110] proposed the HEAL method, introducing contrastive learning to protein function prediction. For a single protein graph, HEAL generates an alternative view via smooth perturbation. Through contrastive learning, HEAL learns higher-quality and more discriminative protein graph representations. By forcing the model to recognise similarities between different “views” of the same protein and push apart representations of distinct proteins, HEAL enables the model to focus on intrinsic, invariant structural and functional features rather than over-relying on noise or specific details in training data, thereby enhancing generalisation.

Wang et al. [111] applied this approach to molecular machine learning. Specifically, in molecular graph modelling, atom masking, bond deletion, and subgraph removal are employed as augmentation methods:

Atom masking: Randomly masks a subset of atomic features with a special token, compelling the model to infer masked atom information from local contexts.

Bond deletion: Randomly removes a fraction of chemical bonds, simulating bond cleavage—a more aggressive augmentation requiring the model to understand atomic connectivity.

Subgraph removal: Removes a continuous subgraph (including atoms and bonds) starting from randomly selected atoms, combining atom masking and bond deletion to force attention on key functional motifs.

Two views of the same molecule generated via these augmentations form a positive pair, while any two views from different molecules in a batch are treated as negative pairs.

Also in 2023, Liu et al. [112] proposed the Muse-GNN model, applying contrastive learning to gene association prediction. Here, contrastive learning serves as a self-supervised strategy, maximising mutual information between functionally similar genes via positive-negative graph interactions while distinguishing dissimilar genes, thereby enhancing cross-modal integration of gene representations.

In multi-omics analysis, beyond contrastive learning between two augmented graphs, some models construct graphs of the same biological entities across different omics layers (e.g., genomics and transcriptomics) and perform cross-omics contrastive learning on them. In 2024, Chen Zhao et al. [113] proposed the CLCLSA model, which integrates embeddings from multiple omics data through contrastive learning combined with self-attention. Specifically, the model first applies a feature-level self-attention mechanism to learn the importance of each feature within each omic, suppressing noisy and low-informative features; then, a cross-omics self-attention layer captures inter-omic relationships. The two-level weighted embeddings are concatenated into a unified representation and fed into a classifier. In 2025, Zhenglong Cheng et al. [114] introduced scMGCL, which extends this idea to single-cell multi-omics analysis. scMGCL treats RNA (scRNA-seq) and ATAC (scATAC-seq) as two mutually enhanced modalities, representing the transcriptomic and epigenomic layers, respectively, to interpret gene expression regulation at the cellular level. Each modality constructs its own KNN graph and encodes it through a GCN, after which contrastive learning is performed by taking embeddings of the same cell across modalities as positive pairs and embeddings of different cells across modalities as negative pairs. The model employs a contrastive loss to maximise the similarity of positive pairs and minimise that of negative pairs, thereby aligning cross-modal cellular representations in a unified latent space.

Additionally, Deep Graph Infomax [97] (DGI) employs a contrastive approach that contrasts augmented graphs with corrupted graphs. To distinguish true local-global relationships from spurious correlations, DGI generates negative graphs via structural reorganisation of the original graph. A discriminator models the association between true/false sample pairs and full graph embeddings, capturing global structural relationships by maximising positive class probabilities and minimising negative ones:

(103) $$L=\frac{1}{N+M}\left(\sum_{i=1}^{N}E_{\left(X,A\right)}\left[logD\left({\tilde{h}}_{i},\vec{s}\right)\right]+\sum_{j=1}^{M}E_{\left(\bar{X},A\right)}\left[log\left(1-D\left({\tilde{\bar{h}}}_{j},\vec{s}\right)\right)\right]\right)\;where\;D\left({\overset{\to }{h}}_{i},\overset{\to }{s}\right)=\sigma \left({\overset{\to }{h}}_{i}^{T}W\overset{\to }{s}\right)$$

Here, $\left({\tilde{h}}_{i},\vec{s}\right)$ denotes a positive pair (node ${\overset{\to }{h}}_{i}$ exists in the graph $\overset{\to }{s}$), so it should output a probability near 1, and the first term maximises this. Conversely, it is a negative pair (node is “generated” or “corrupted” and does not belong to $\overset{\to }{s}$).

In 2022, Liu et al. developed GraphCDR [115] based on this method for drug discovery. The model constructs a corrupted CDR graph from resistant responses in the training set. Since resistant responses inherently carry opposite information to sensitive responses, contrasting the original (sensitive-response-based) graph with the corrupted graph enhances embedding discriminability.

In 2022, Ningyu Zhang et al. [115] proposed the OntoProtein model, the first knowledge-graph-based protein pre-training model. This approach applies knowledge graphs to the process of generating negative samples by incorporating the concept of entity-relation triples. The graph is represented as a combination of multiple triples, where entities include Gene Ontology (GO) terms and proteins. Negative samples are generated by replacing the head or tail entities (i.e., nodes in the graph) within the triples. The model aims to minimise the distance d(h, t) of positive samples/true triples while maximising the distance of negative samples/false triples.

(104) $$L_{KE}=-log\sigma (\gamma -d(h,t))-\frac{1}{n}\sum_{i=1}^{n}log\sigma (d(h_{i}^{\prime },t_{i}^{\prime })-\gamma )\;where\;d_{r}\left(h,t\right)=\left\|h+r-t\right\|$$

Here, *h* represents the embedding of the head entity, *t* the embedding of the tail entity, and *r* the embedding of the relation.

Furthermore, this model also optimises the sampling strategy. When constructing negative samples, replacements are made only within the same semantic branch. For instance:

For GO–GO relations, only nodes belonging to the same category (MFO, CCO, BPO) are replaced.

For Protein–GO relations, only the tail entity (the GO term) is replaced, while ensuring its category remains consistent.

This approach generates negative samples that are biologically and semantically closer to the positive samples, making them more challenging. This, in turn, forces the model to learn more discriminative protein-function representations, thereby significantly enhancing the quality of the knowledge embeddings and the performance of downstream prediction tasks.

#### 3.4.6. Transformer-Enhanced GNNs

Recently, the incorporation of Transformer architectures into graph neural networks (GNNs) has attracted growing attention in the field of bioinformatics. The Transformer is a sequence model based purely on the attention mechanism, proposed by Ashish Vaswani et al. [64], and was initially applied to the field of natural language processing (NLP). Compared with CNNs and RNNs, the Transformer can capture global dependencies within a sequence using only a single layer, making it more effective than CNN-based methods for identifying global sequence patterns relevant to functional prediction.

In 2019, Xiaolong Fan et al. [116] first integrated the Transformer into graph neural networks (GNNs) to address the traditional GNNs’ inability to effectively distinguish the importance of nodes or layers. Their model focused more on key node variables within a graph, significantly improving performance, interpretability, and the ability to capture information across multi-hop neighbours, thereby overcoming the limitations of local convolution.

**Figure 12:**
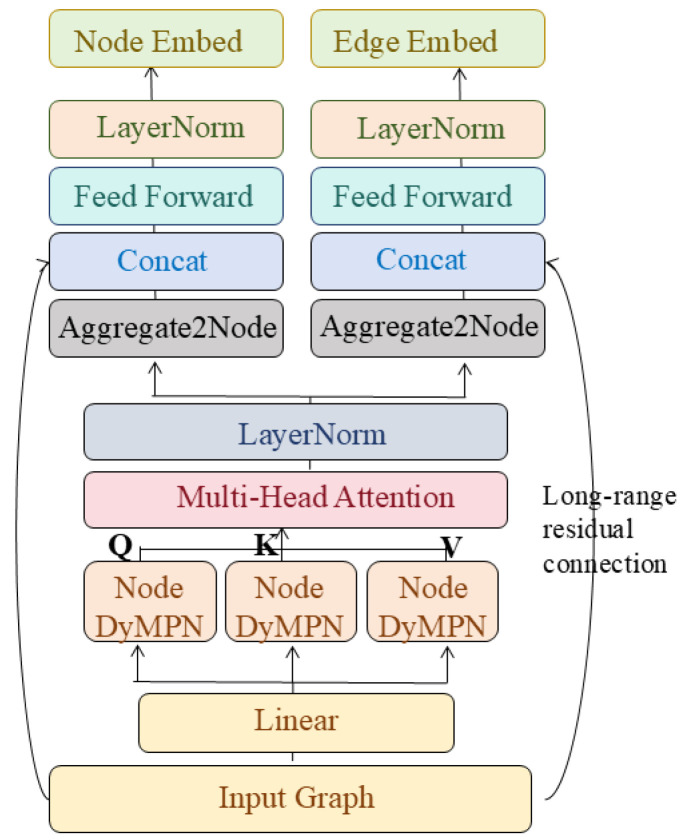
The pipeline of GROVER.

In 2020, Yu Rong et al. [117] proposed GROVER, as Figure 12 shows, applying this innovative hybrid framework to molecular representation learning. Specifically, a customised GNN (called dyMPN) was used to extract local structural information from molecular graphs, generating query, key, and value vectors, which were then fed into a Transformer encoder to model global inter-node relationships. This approach effectively mitigated the issues of limited labelled data and poor generalisation to novel molecules.

In 2021, Yue Cao and Yang Shen [118] introduced TALE, a Transformer–GNN-based model for protein function annotation. Without relying on structural or network data, TALE achieved high accuracy and generalisation using only protein sequence information. The method employs Transformer encoder layers to process position-encoded amino acid sequences, generating sequence embeddings. Then, Gene Ontology (GO) term embeddings are mapped into the same latent space as the protein embeddings. A similarity matrix (M) is computed between the protein representation (P) and GO term representation (Q), followed by 1D convolution and max-pooling to derive attention weights (a). The final sequence–label joint representation is obtained as ($e\;=\;a^{T}P$).

Recently, the Transformer-GNN models have been increasingly adopted in drug–target affinity (DTA) prediction. Generally, two major integration strategies exist:

Protein-side Transformer integration [119,120]: The Transformer is applied to encode macromolecular targets. Since target protein sequences contain long-range dependencies—such as distant residues forming a shared active site—the Transformer effectively captures contextual and domain-level interactions, while GNNs aggregate drug molecular features from graph representations. The combined features are fused through fully connected layers to yield a binding affinity representation.

Joint Transformer integration [121,122]: The Transformer is applied to fused drug–target representations. Here, GNNs are first used to analyse different types of graphs (e.g., molecular graphs, drug–disease association graphs, and drug–drug interaction graphs). These heterogeneous views are projected into a unified feature space and subsequently fed into the Transformer to capture global dependencies and multi-view correlations, thereby enabling fine-grained modelling of drug–target interactions.

#### 3.4.7. Hypergraph Neural Networks for Higher-Order Biological Interactions

While most graph neural network (GNN) formulations model biological systems using pairwise relationships, many biomolecular processes naturally involve higher-order interactions that cannot be fully captured by simple edges. Examples include protein complexes composed of multiple subunits, combinatorial gene regulation involving several transcription factors, metabolic reactions with multiple substrates and products, and pathway-level regulatory modules. Hypergraphs provide a natural representation for such systems by allowing hyperedges to connect more than two nodes simultaneously.

Hypergraph neural networks (HNNs) extend standard GNN frameworks by enabling message passing over hyperedges, thereby modelling group-wise dependencies and collective interactions [123,124]. Representative approaches include clique-expansion-based methods, star-expansion formulations, and direct hyperedge-level aggregation schemes, each offering different trade-offs between expressive power and computational complexity. In bioinformatics, HNNs have been applied to tasks such as gene function prediction, disease–gene association analysis, and cancer-related gene prioritisation, where higher-order biological context plays an important role [124].

Despite their conceptual appeal, hypergraph-based models are often simplified to pairwise graph representations in practical pipelines, motivated by scalability constraints, data sparsity, and compatibility with mature GNN architectures. In many studies, hyperedges derived from pathways, complexes, or multi-omics groupings are projected into weighted graphs or factorised into bipartite structures before learning. As a result, graph-based and hypergraph-based approaches should be viewed as complementary rather than mutually exclusive, with the choice depending on data availability, biological resolution, and computational considerations.

In this review, we focus primarily on graph-based neural representations while explicitly acknowledging hypergraph neural networks as an important extension for modelling higher-order biological interactions. Rather than presenting a full taxonomy of HNN methods, we highlight their conceptual role and practical integration within graph-based bioinformatics workflows, and refer interested readers to recent dedicated studies for more detailed treatment.

## 4. Existing GNNs in Bioinformatics Tasks

The ultimate value of graph neural networks in bioinformatics lies not in architectural novelty but in their ability to address long-standing biological questions that are difficult to resolve using conventional modelling approaches. Across molecular, cellular, and clinical domains, GNNs have enabled a shift from feature-centric prediction toward relational and systems-level inference, allowing researchers to reason explicitly about interactions, pathways, and network context.

Across application domains, GNN-based approaches demonstrate consistent value in relational modelling, while sharing common limitations related to data bias, validation, and biological interpretability.

In this section, we organise existing applications of GNNs around core bioinformatics tasks, highlighting how graph-based learning reframes biological questions, what types of biological insight can be gained, and where current approaches remain limited.

Unlike traditional deep learning models that primarily handle Euclidean data such as images or sequences, GNNs can effectively capture topological dependencies and relational information within non-Euclidean biological networks. This capability has led to a surge of studies applying GNNs across diverse areas of bioinformatics, ranging from molecular-level chemical property prediction to system-level disease association analysis. In studies focusing on drug-related applications, the roles of graph neural networks are typically categorised into molecular property and activity prediction, interaction prediction, synthetic route prediction, and de novo drug design [7,125]. Meanwhile, in bioinformatics research, their applications are mainly divided into disease association prediction, drug development, and medical imaging [126].

Therefore, based on recent surveys and representative studies, the overall applications of GNNs can be systematically summarised into five major domains:

(1) disease association prediction, enabling the discovery of potential disease-related genes and RNAs;

(2) drug development and discovery, supporting drug–target interaction modelling and de novo molecule design;

(3) protein structure and function analysis, assisting in protein folding, binding, and interaction prediction;

(4) multi-omics analysis, facilitating integrative learning across heterogeneous biological data;

(5) medical imaging and multimodal fusion, advancing precision diagnosis through structural and phenotypic graph representations.

In the following sections, we will systematically elaborate on the role and methodologies of GNNs in each of these five domains, highlighting representative models, datasets, and future research directions.

### 4.1. Disease Association Prediction

Discovering factors associated with various diseases is an important task in bioinformatics. Because the causes of diseases are numerous and complex, conventional machine learning models fail to capture the intricate topological relationships among pathogenic molecules and factors. Therefore, graph neural networks are introduced to represent the nonlinear relationships between diseases and other entities in biological networks, incorporating convolution operations to process features across subgraphs.

In disease association prediction, GNN-based models shift the analytical focus from isolated gene-level signals to network-contextualised disease mechanisms. By embedding genes, proteins, and phenotypes within interaction networks, these approaches enable the identification of disease-associated modules rather than single candidate genes. Such network-level representations support biological interpretation by highlighting dysregulated pathways and interaction patterns, thereby facilitating hypothesis generation for downstream experimental validation and therapeutic targeting.

In such prediction tasks, biomedical entities are typically represented as nodes, including diseases, genes/protein-coding genes (PCGs), and miRNAs (sometimes also lncRNAs, proteins, or individual samples). The edges are generally divided into two categories: similarity edges, which describe similarities between entities (e.g., disease–disease or miRNA–miRNA), and association edges, which represent relationships between different types of entities (e.g., known disease–gene (D–G) or disease–miRNA (D–M) associations). The objective is to train a GCN model to score and rank unknown disease–molecule (gene/miRNA, etc.) pairs, thereby uncovering potential associations that can provide insights into disease mechanisms and therapeutic strategies.

While GNN-based disease association models effectively integrate heterogeneous biological and clinical data, their reliability remains constrained by incomplete ground truth, population bias, and the difficulty of disentangling correlation from causal molecular mechanisms.

In general, studies on the mechanisms of disease onset follow two major directions: the miRNA–disease route and the gene–disease route, which explain disease mechanisms from the perspectives of causation and expression, respectively. These approaches, empowered by GNN-based modelling, provide a deeper understanding of the molecular interactions underlying complex diseases.

#### 4.1.1. miRNA–Disease

In 2019, Xiaoyong Pan and Hong-Bin Shen proposed DimiG [11], the first graph convolutional network model for inferring disease-associated microRNAs. Instead of relying on known miRNA–disease pairs, DimiG integrates PCG–PCG, PCG–miRNA, and PCG–disease networks with tissue expression profiles to transfer disease labels from protein-coding genes to miRNAs. The model formulates prediction as a node-classification problem on a heterogeneous graph and achieves superior performance over traditional random-walk and matrix-factorisation baselines. Extensive case studies on cancers and inflammatory diseases confirm its robustness and biological interpretability.

In 2021, Yulian Ding et al. proposed VGAE-MDA [127]. It constructs subnetworks based on the similarities of miRNAs and diseases, respectively, and employs two variational graph autoencoders (VGAEs) to calculate association scores for each subnetwork. Finally, the model integrates these results to obtain the overall miRNA–disease association scores. While Lei Li proposed GCAEMDA [128], different from VGAE-MDA, GCAEMDA uses the discriminative model Graph Convolutional Autoencoder (GCAE), which is more efficient and suitable for large-scale graph tasks. Yanyi Chu et al. proposed MDA-GCNFTG [129], which introduces the Gaussian Interaction Profile (GIP) kernel similarity to capture the statistical principle that “similar miRNAs tend to be associated with similar diseases”. It enables the model to predict new diseases without known related miRNAs, as well as new miRNAs without known associated diseases.

In 2022, Guanghui Li et al. proposed GATMDA [130], which constructs linear features of diseases and miRNAs based on disease-lncRNA and miRNA–lncRNA association networks and captures nonlinear features using a graph attention network (GAT). The linear and nonlinear features are then integrated through a random forest algorithm to infer potential disease–miRNA associations.

In 2024, Yufang Zhang proposed ReHoGCNES [131], proposed ReHoGCNES, regularising similarity and association graphs via kNN-based degree equalisation to enhance training stability and performance. Separately, Tian and Han et al. introduced MGCNSS, constructing reliable negative samples from unlabelled data using distance-based selection.

#### 4.1.2. Gene–Disease

In 2019, Vikash Singh and Pietro Lio were the first to apply a generative graph neural network to disease–gene prediction DGP [132]. They introduced the variational graph autoencoder VGAE and its extension for heterogeneous graphs C–VGAE, which effectively prioritises candidate genes for various diseases through powerful latent embeddings learned from disease-gene networks, demonstrating superior performance compared to baseline random walk--based methods.

In 2024, PiJing Wei et al. [133] proposed PDGCN. It integrates multiple types of data features, including mutation and expression profiles, together with network structural features extracted using Node2vec, to construct a sample-gene interaction network. The prediction is performed using a graph convolutional neural network model with a conditional random field (CRF) layer, which demonstrates significantly better performance than comparable methods on cancer-related databases. Then, CuiXiang Lin et al. proposed LIMO–GCN [134], a hybrid framework that combines the robustness of linear models against overfitting with the representation power of graph convolutional networks. The method achieved state-of-the-art performance in predicting Alzheimer’s disease-associated genes and further validated its predictions using molecular evidence derived from heterogeneous genomic data, demonstrating the model’s accuracy and biological reliability. The performance of each method is shown in Table 8.

It should be noted that most node-level benchmarks (e.g., citation networks, molecular property graphs) report accuracy or F1 due to class imbalance, whereas graph-level classification tasks often adopt ROC-AUC to accommodate multi-label settings and varying positive–negative ratios. Regression-based molecular property prediction typically uses MAE or RMSE. Readers should note that datasets differ substantially in task formulation, graph size, label sparsity, and heterogeneity; therefore, results across datasets are not directly comparable and should be interpreted within their task-specific context.

In disease association prediction, tasks often involve binary or highly imbalanced link prediction. The area under the ROC curve (AUC) measures the model’s ability to discriminate positive and negative associations, while the precision-recall curve (PRC) is particularly informative under strong class imbalance by emphasising performance on the positive class.

### 4.2. Drug Development and Discovery

Drug property prediction can be regarded as a subcategory of protein structure and function prediction. However, due to its broad application prospects and distinct research focus compared with other types of protein function analysis, it is often treated as an independent field.

In drug design, graph-based modelling generally falls into two categories. The first is the molecular graph, where atoms or functional groups are represented as nodes and chemical bonds as edges. The goal is to predict the intrinsic properties of a single drug molecule. The second is the relation graph, in which nodes represent drugs and biological targets, while edges denote interactions such as drug–drug synergy/antagonism or drug–target associations. The feature matrix encodes the attributes of each biological entity—for drugs, these include atom type, bond type, degree, and aromaticity; for target proteins, sequence and structural descriptors are typically used.

GNNs have shown promise in accelerating molecular screening and drug-target prioritisation, but their practical impact is limited by dataset bias, sparse experimental validation, and challenges in translating predicted affinities into actionable pharmacological insights.

Commonly, based on the specific application scenario, drug property prediction tasks can be categorised into three major types: drug-drug interaction (DDI) prediction, drug-target interaction (DTI) prediction, and drug design and repositioning.

#### 4.2.1. DTI Prediction

In 2019, Wen Torng et al. first utilised GCNs to predict drug-target interactions [144]. They trained two GCNs, one driven by a combination of classification labels, to automatically extract features from a pocket graph and the other from a two-dimensional ligand graph. This allowed them to predict protein-ligand interactions. In 2022, Tuan Nguyen et al. proposed GraphDRP [145], which uses a molecular graph and a drug–cell target bilayer graph: drugs are first represented as molecular graphs, directly capturing the bonds between atoms. Cell lines are then described as binary vectors of genomic aberrations. Finally, a relationship graph is used to combine drug-cell line pairs and predict their response values. In 2024, Haiou Qi proposed GLCN–DTA [146], which integrates graph learning modules into existing graph architectures. This approach aims to learn a soft adjacency matrix for protein and drug molecular graphs, thereby improving the model’s generalisation capabilities.

#### 4.2.2. DDI Prediction

In 2018, Marinka Zitnik et al. proposed the Decagon method for modelling the side effects of polypharmacy [147]. This method constructs a multimodal graph of protein-protein interactions, drug-protein-target interactions, and polypharmacy side effects, ultimately representing the drug–drug interaction model. By incorporating marker-protein and drug–target information, this method can predict not only side effects but also the specific type of side effects. In 2022, Qidong Liu et al. proposed the M2GCN method [148]. Similarly, M2GCN constructs three relationship graphs to supplement drug association information, performs graph aggregation on each subgraph, and then uses consistency regularisation to align different subgraphs, further improving model performance.

In 2022, Shaghayegh Sadeghi proposed DDIPred [149], which constructs only a drug–drug feature graph, allowing it to learn drug relationships from molecular information itself. At the same time, feature representation was optimised: Self-referencing embedded strings (SELFIES) and a vector representation of drug text data (Doc2Vec) were used to extract drug SMILES, and the optimised feature matrix was then used in GCN training. In 2023, Yi Zhong et al. proposed DDI-GCN [150], which combines GCN with BiGRU–Att adaptive receptive field selection and co-attention on molecule pairs to highlight substructures that lead to interactions. In 2025, Yeon Uk Jeong et al. proposed a new DDI method [151]: Based on the entire DDI graph, it generates an “interaction candidate list” for each drug, directly assigning scores to all unknown drug pairs, effectively addressing the data imbalance problem.

#### 4.2.3. Drug Design and Repositioning

In 2024, Xinliang Sun proposed AdaDR [152], which treats drug repositioning as drug–disease link prediction on heterogeneous graphs, learning over two spaces: a topology GCN on the known drug–disease bipartite network, and a feature-GCN on kNN graphs built from drug/disease similarities. An attention module adaptively fuses both embeddings, with a consistency constraint aligning them, yielding superior AUROC/AUPRC and validated case studies. In 2024, Jiyeon Han proposed DRSPRING [153], which targets combination therapy: a first GCN predicts drug-induced gene-expression profiles; a second, integrated DrugCellGCN, fuses chemical graphs, gene–gene networks, drug–target links, basal expression, and (measured/predicted) perturbation profiles to predict Loewe synergy, generalising to unseen drugs/cell lines and surpassing baselines.

The performance of each method is shown in Table 9. In drug development and discovery, models support drug–target interaction prediction or molecular property regression. Classification-based tasks adopt AUC and PRC, whereas regression tasks use the root mean squared error (RMSE) to quantify the deviation between predicted and true molecular properties. The Concordance Correlation Coefficient (CCp) further evaluates the degree of agreement between predictions and ground truth by combining accuracy and precision.

In conclusion, in drug development and discovery, GNNs provide a unified framework for integrating molecular structure, target interaction, and biological context. Beyond improving compound screening efficiency, graph-based models support mechanism-oriented reasoning by identifying structure–activity relationships, off-target effects, and network-level drug responses. This systems-level perspective is particularly valuable for drug repurposing and polypharmacology, where therapeutic efficacy emerges from coordinated modulation of multiple targets.

### 4.3. Protein Structure and Function Analysis

Proteins perform many essential roles in living organisms, such as catalysing chemical reactions, coordinating signalling pathways, and providing structural support to cells. To elucidate the mechanisms of life, it is crucial to analyse protein structure and function.

In graph-based protein structure–function analysis, the nodes are typically amino-acid residues (sometimes atoms or surface points/patches), while the edges encode covalent bonds, sequence adjacency, and/or 3D proximity (e.g., k-nearest neighbours or distance/contact thresholds); for PPIs, cross-protein candidate edges represent residue–residue contacts. Graph-based models capture structural dependencies and interaction patterns in proteins, yet their biological interpretability and generalisation remain sensitive to data quality, structural resolution, and the availability of experimentally validated annotations.

In general, protein structure–function analysis can be grouped into three categories: protein–protein interaction prediction (PPI), protein function prediction, and protein design.

#### 4.3.1. Protein–Protein Interaction

Protein–protein interactions (PPIs) are a cornerstone of protein analysis and drug discovery: they shape signalling, receptor binding, and immune transmission. In 2017, Alex Fout et al. first applied graph convolutional networks to PPIs [162]: nodes are sequence-annotated residues and edges denote residue–residue relations; the model classifies whether any cross-protein residue pair forms an interface contact, outperforming SVM baselines. In 2021, Federico Baldassarre proposed GraphQA [163], using GCNs on residue–contact graphs to jointly learn local and global quality scores, thereby prioritising more plausible protein structures. In 2021, Bowen Dai and Chris Bailey-Kellogg introduced Pinet [164], upgrading from residue graphs to protein surface point clouds that encode geometry, charge, and hydrophobicity, and predicting partner-specific interface regions (not just residue contacts), enabling transfer to tasks like antibody–antigen interfaces. In 2022, Kanchan Jha et al. encoded each protein’s residue graph with GCN/GAT [165], applied global pooling to obtain protein embeddings, concatenated them, and predicted pairwise interaction—moving beyond residue-pair prediction to protein-pair-level PPI classification.

#### 4.3.2. Protein Function Prediction

Active sites, pockets, and binding interfaces in a protein’s tertiary/quaternary structure largely determine its function; these sites are often conserved in both sequence and structure. Therefore, graph-based modelling of protein structure and sequence is crucial for function prediction.

In 2021, Vladimir Gligorijević et al. introduced DeepFRI [166], an early GCN-based framework for protein function prediction that fuses protein language-model embeddings with structure-derived features, outperforming sequence-only CNN baselines. Ronghui You proposed DeepGraphGO [167], adopting a multi-species training strategy—one model across organisms—to boost performance for sparsely annotated species. In 2022, Chenguang Zhao et al. presented PANDA2 [168], which models the Gene Ontology DAG with GNNs to learn hierarchical representations while integrating Transformer-based protein embeddings. In 2023, Peishun Jiao et al. proposed Struct2GO [169], converting AlphaFold2 structures to residue-contact graphs with hierarchical self-attention pooling and fusing them with SeqVec for multi-label GO prediction—effective even without PPI data. In 2024, Yaan J. Jang introduced PhiGNet [106], a statistics-informed graph network that predicts function directly from sequence, enabling structure-free inference.

#### 4.3.3. Protein Design

In 2019, Ingraham et al. introduced a Structured Transformer for graph-based protein design [170]. They represented backbones as k-NN residue graphs with geometric edge features (distances, directions, orientations) and used graph attention with an autoregressive decoder to model p (sequence structure), achieving strong sequence recovery and generalisation to unseen folds with large speedups over Rosetta. In 2020, Strokach et al. proposed ProteinSolver [171], framing inverse folding as a constraint satisfaction problem on residue–residue interaction graphs. Trained via masked reconstruction on massive sequence–structure data, it rapidly generates sequences consistent with target folds, matches or outperforms traditional pipelines on stability/design benchmarks, and includes experimental validation and accessible tooling.

The performance of each method is shown in Table 10. In protein structure and function analysis, evaluation metrics reflect the complexity of structural and binding prediction tasks. AUC and AUPR are used for classification scenarios such as interface or binding site prediction. Regression metrics such as RMSE assess quantitative structural deviations. Domain-specific metrics, including R-Target, Fmax, and Smin, measure alignment consistency, the balance between precision and recall under optimal thresholds, and the minimal semantic distance between predicted and true functional annotations, respectively.

In conclusion, for protein structure and function analysis, GNNs enable learning representations that couple spatial organisation with biochemical properties, supporting functional inference beyond static structural prediction. By modelling residue interactions and structural motifs as graphs, these approaches facilitate the identification of functional sites, allosteric mechanisms, and structure–function relationships. Such insights are critical for understanding protein dynamics and guiding experimental design in structural biology.

### 4.4. Multi-Omics Analysis

The integration of multi-omics data offers a comprehensive view of biological systems, but the data are inherently heterogeneous and highly interconnected. Graph Convolutional Networks effectively model these complex relationships by representing genes, metabolites, microbes, and clinical variables as nodes in a graph, with biological interactions as edges. By learning feature propagation across multi-layer biological networks, GCN/GNN enable accurate prediction of disease-associated microbes, key biomarkers, and molecular subtypes. Thus, they provide a powerful framework for uncovering hidden structures within multi-omics systems.

In 2021, Tongxin Wang et al. proposed MOGONET [178], which applied GCN to omics-specific learning and cross-omics association learning for the first time, achieved effective multi-omics data classification, and proved to be superior to other methods in different biomedical classification applications of mRNA expression data, DNA methylation data, and microRNA expression data. In 2022, Xiao Li and Jie Ma developed a multi-omics integration model MoGCN [179] based on the graph convolutional network (GCN) for cancer subtype classification and analysis, using an autoencoder (AE) and similarity network fusion (SNF) methods to reduce the dimension of multi-omics data and construct patient similarity networks. In 2024, Bingjun Li and Sheida Nabavi used biological molecules as nodes (genes, miRNAs) to construct a heterogeneous multi-layer supra-graph containing both intra-group (gene-gene, miRNA-miRNA) and cross-group (miRNA–gene) connections, and compared GCN and GAT [180]. The conclusion is that GAT is better for small graphs/less information; GCN is more stable for large graphs/more information.

In multi-omics analysis and medical imaging with multimodal fusion, tasks often involve phenotype prediction, patient stratification, or survival analysis. Here, F1-score and Accuracy summarise classification performance, AUC captures discriminative power under imbalanced conditions, and the concordance index (c-index) quantifies survival or risk-ranking consistency in clinical outcome prediction. Due to the common use of the same dataset, the performance of this category model was summarized together with the multimodal transport category.

GNNs enable integrative analysis across heterogeneous omics layers, but inferred cross-modal relationships often reflect statistical association rather than direct molecular causality, necessitating careful validation and biological contextualisation.

In conclusion, in multi-omics analysis, GNNs address the challenge of integrating heterogeneous molecular layers by explicitly modelling cross-modal relationships. Graph-based integration enables the discovery of coordinated regulatory programmes that span genomic, transcriptomic, epigenomic, and proteomic data. This relational perspective supports biologically interpretable patient stratification and provides a foundation for hypothesis-driven investigation of disease heterogeneity and treatment response.

### 4.5. Medical Imaging and Multimodal Fusion

In the modern medical field, with the widespread adoption of medical imaging, increasingly complex and diverse data have been generated. Each patient now produces data that exhibits high-dimensional, multimodal, and heterogeneous characteristics. A single modality often fails to comprehensively reveal disease states, whereas integrating multimodal information, such as imaging, clinical, and omics data, holds the potential to overcome this limitation, thereby improving diagnostic accuracy, prognosis prediction, and personalised treatment performance. In imaging-based and multimodal applications, graph neural networks enable the abstraction of imaging data into biologically meaningful relational representations. By modelling spatial or population-level relationships among regions, cells, or patients, these approaches move beyond pixel-level pattern recognition toward biomarker discovery and systems-level interpretation. When integrated with molecular or clinical data, graph-based models provide a bridge between imaging phenotypes and underlying biological mechanisms, aligning imaging analysis with the broader goals of biomolecular research. Among them, Graph Neural Networks (GNNs) and their classical variant, the Graph Convolutional Network (GCN), demonstrate unique potential in multimodal medical analysis due to their natural ability to model relationships among entities and capture structured interactions.

Graph-based multimodal frameworks support joint reasoning over imaging, molecular, and clinical features, although weak supervision, limited interpretability, and scarce prospective validation continue to hinder clinical translation. Based on the scope and domain of the constructed graphs, the studies can be categorised into three types: population graphs, brain graphs, and pathology/WSI graphs. Population graphs refer to subject-level graphs where each node represents an individual patient and edges encode demographic or clinical similarity. Brain graphs denote functional or structural connectivity networks derived from neuroimaging modalities, with nodes corresponding to anatomical regions and edges representing correlations or fibre connections. Whole-slide image (WSI) graphs model histopathology images by treating patches or superpixels as nodes with spatial adjacency or morphological similarity as edges. These definitions standardise our discussion across heterogeneous biomedical graph settings. In Table 11, we also compared those models in this application field.

#### 4.5.1. Population Graph Analysis

In 2017, Sarah Parisot [181] combined multimodal medical analysis with GCNs. Using brain imaging data, she formulated population-level disease prediction as a semi-supervised node classification problem on a population graph, where each node represented an individual patient and the edges encoded phenotypic similarities (e.g., gender, acquisition site, age). The model achieved state-of-the-art performance on two datasets, ADNI (Alzheimer’s Disease Neuroimaging Initiative) and ABIDE (Autism Brain Imaging Data Exchange). In 2019, Anees Kazi et al. built upon this work and proposed a novel InceptionGCN [182] model featuring multiple convolutional kernel sizes. Within the same layer, different nodes were convolved with different receptive field sizes in parallel, making the model more adaptable to the heterogeneity of real-world population graphs.

#### 4.5.2. Brain Graph Analysis

In 2018, Sofia Ira Ktena et al. applied GCNs to brain analysis [183], where each node represented a brain region of interest (ROI), and edges denoted spatial proximity or average functional connectivity between ROIs. Each node carried a feature vector encoding its functional connectivity strength with all other ROIs. In 2021, to understand the association between brain regions and specific neurological disorders or cognitive stimuli, Xiaoxiao Li proposed BrainGNN [184], which analyses fMRI data to discover neural biomarkers. To leverage both the topological and functional characteristics of fMRI, the study introduced a novel ROI-aware graph convolution (Ra-GConv) layer.

In 2023, Hejie Cui et al. proposed BrainGB [185], the first unified and reproducible benchmark framework for brain network GNNs. It systematically standardised the construction process for both functional and structural brain networks and decomposed GNN design into four modular components: node features, message passing, attention, and readout.

In 2024, Kaizhong Zheng proposed BPI-GNN [186], which introduced prototype learning into brain network GNNs to refine the stratification of depression. The model simultaneously distinguished patients from healthy controls and automatically identified biologically meaningful disease subtypes. Furthermore, it designed a subgraph generation process to select discriminative connections (edges representing ROI functional connectivity).

#### 4.5.3. Whole Slide Image (WSI) Analysis

In 2021, Richard J. Chen et al. proposed Patch-GCN [187], which serves as a fundamental approach for WSI analysis. The model treats an entire whole-slide histopathology image as a “point-cloud-like” patch graph for survival prediction. Each patch is considered a node and connected based on its true spatial adjacency within the slide, forming a homogeneous graph in which all nodes share the same semantic type (image patches) and edges represent a single relationship (spatial proximity). CNNs extract patch-level features, followed by GCN-based residual message passing and global attention pooling to produce patient-level representations.

In 2022, Yonghang Guan et al. introduced the Node-Aligned GCN [188], which performs global clustering on all patch features to construct a visual vocabulary. Each WSI’s instances are then divided into several sub-bags according to the vocabulary, establishing cross-slide node alignment. Representative instances are selected from each sub-bag as nodes, addressing instability caused by extremely large WSIs, slide-level labels, and inconsistent node definitions across slides. In 2023, Tsai Hor Chan proposed an Heterogeneous Graph Representation Learning framework for WSI analysis [189], overcoming the limitations of prior homogeneous graph + cluster pooling methods that failed to capture the diversity of cell types and relationships and tended to overparameterize.

In 2024, Xiao Xiao et al. proposed the TCGN model [190], which optimised the network structure by integrating CNN + Transformer + GNN within a unified vision backbone. The model combines convolutional and Transformer modules with graph–node co-embedding to predict gene expression from a single local Spot image, achieving both high accuracy and interpretability without relying on spatial coordinates or global contextual information.

**Table 11:** Performance Comparison of Predictive Models for Multi-omics & multimodal fusion.

Methods	Category	Training Datasets	Metrics	Headline Result
GCN [49]	population diagnosis	ABIDE	Accuracy	0.704
			AUC	0.75
		ADNI	Accuracy	0.8
			AUC	0.875
InceptionGCN [182]	population diagnosis	TADPOLE	Accuracy	0.8853
		ABIDE	Accuracy	0.6928
s-GCN [183]	brain network metric learning	ABIDE	AUC	0.58
		UK biobank	AUC	0.73
BrainNetCNN [191]	brain network metric learning	Biopoint	F1-score	0.6558
		HCP		0.9096
GAT [65]	brain network metric learning	Biopoint	F1-score	0.7508
		HCP		0.77
GraphSAGE [60]	brain network metric learning	Biopoint	F1-score	0.7555
		HCP		0.886
PR-GNN [192]	brain network metric learning	Biopoint	F1-score	0.752
		HCP		0.9109
BrainGNN [184]	brain network metric learning	Biopoint	F1-score	0.758
		HCP		0.9434
SIB [193]	brain network metric learning	ABIDE	F1-score	0.62
		Rest-meta-MDD		0.65
		SRPBS		0.69
ProtGNN [194]	brain network metric learning	ABIDE	F1-score	0.68
		Rest-meta-MDD		0.59
		SRPBS		0.79
BrainIB [195]	brain network metric learning	ABIDE	F1-score	0.71
		Rest-meta-MDD		0.65
		SRPBS		0.84
BPI-GNN [186]	brain network metric learning	ABIDE	F1-score	0.72
		Rest-meta-MDD		0.72
		SRPBS		0.92
DeepGraphConv [196]	WSI, Cancer Survival Prediction	TCGA	c-Index	0.62
Patch-GCN [187]	WSI, Cancer Survival Prediction	TCGA	c-Index	0.636
Cluster-to-conquer GCN [197]	WSI, Cancer Typing Analysis	TCGA-NSCLC	Accuracy	0.873
			AUC	0.938
		TCGA-RCC	Accuracy	0.919
			AUC	0.987
Node-Aligned GCN [188]	WSI, Cancer Typing Analysis	TCGA-NSCLC	Accuracy	0.902
			AUC	0.952
		TCGA-RCC	Accuracy	0.954
			AUC	0.992
HEAT [189]	WSI, Cancer Typing Analysis	TCGA	AUC	0.928
			Accuracy	0.927
			Macro-F1	0.933
MoGCN [179]	Multi-omics Cancer Subtype Classification	TCGA (BRCA, pan-kidney)	F1-score	0.901
SNF+GCN [198]	Multi-omics Cancer Subtype Classification	TCGA (BRCA, pan-kidney)	F1-score	0.889
AE+GCN [199]	Multi-omics Cancer Subtype Classification	TCGA (BRCA, pan-kidney)	F1-score	0.789
GrassmannCluster + GCN [200]	Multi-omics Cancer Subtype Classification	TCGA (BRCA, pan-kidney)	F1-score	0.843

## 5. Discussion: Domain-Specific Considerations of Applying GNNs in Bioinformatics

Graph neural networks have shown strong capability in modelling relational structure in biomedical research. However, biological data possess characteristics that are fundamentally different from social, citation or recommendation system graphs. These include incomplete experimental evidence, context-dependent interactions, dynamic reconfiguration of networks, and stringent requirements for mechanistic interpretability and clinical reliability. In this Discussion Section, we examine the major challenges in applying graph neural networks to biological and clinical environments and provide perspectives for future research directions.

### 5.1. Data and Graph Construction and Representation in Biological Systems

Biological data introduces unique constraints that fundamentally differentiate graph construction from other application domains. Unlike social or citation networks, biological graphs such as gene regulatory networks, protein interactions and cell–cell communication structures are often incomplete, noisy and context dependent. These characteristics impose additional requirements on GNN-based modelling and heavily influence downstream performance.

Therefore, constructing a biologically reliable graph remains one of the central challenges in computational biology. Protein interaction networks merge diverse experimental evidence and contain a significant proportion of interactions with weak or indirect support. Regulatory graphs derived from bulk or single-cell transcriptomic correlations reflect both biological signals and confounding effects from cell type composition or technical noise. Disease comorbidity networks reflect demographic bias rather than true causal associations.

Graph structure learning methods, such as embedding-based rewiring or curvature-based sparsification, mostly optimise mathematical smoothness criteria. These methods often fail to account for biochemical principles. They may introduce edges between co-expressed but functionally unrelated genes or remove rare yet essential regulatory interactions, such as tumour suppressor links.

Future graph construction strategies should integrate curated biochemical knowledge, including pathway annotations, regulatory hierarchies, and experimentally verified interactions. Perturbation data from CRISPR screens, drug inhibition experiments or time-resolved signalling studies can be used to separate causal regulation from correlation. In addition, edges should be represented with uncertainty scores rather than binary values. Multimodal heterogeneous graphs that align genomics, epigenomics, proteomics, metabolomics, imaging and clinical data can further improve biological fidelity.

Although this review primarily focuses on biomolecular, cellular, and clinically oriented graphs that arise in bioinformatics and biomedical research, other large-scale biological graph types, such as ecological food webs or phylogenetic networks, represent complementary domains with distinct modelling assumptions and are promising directions for future exploration of graph neural networks.

### 5.2. Model Design and Architectural Adaptation for Biological Tasks

When designing GNN architectures for bioinformatics, the primary challenge shifts from generic message passing efficiency to the ability to capture hierarchical, multi-scale and heterogeneous biological structures. Biological systems operate across molecular, cellular and tissue levels, requiring models that can integrate structurally diverse node and edge types while preserving biological interpretability.

GNN architectures increasingly incorporate attention mechanisms, learned feature factors or cross-channel aggregators that appear to improve interpretability. Nevertheless, in biological systems, model explanations must reflect experimentally testable mechanisms rather than purely statistical associations. A high attention weight between two proteins may arise from co-localisation or co-expression, yet functional experiments may reveal no direct interaction. Conversely, mean-based aggregation may dilute critical regulatory signals, such as dominant kinases that orchestrate downstream cascades through a small number of high-impact edges.

Ultimately, the relevance of architectural adaptation lies in its ability to preserve biologically meaningful signals that can inform molecular interpretation and downstream experimental investigation, such as dominant regulatory interactions, functional domains, or pathway-level dependencies.

### 5.3. Interpretability and Biological Credibility

A critical distinction between bioinformatics and other machine learning fields is the indispensable requirement for scientific interpretability. Biomedical researchers must understand why a model makes a prediction, which genes or interactions contribute to the decision and how these align with known biological mechanisms. This expectation places interpretability at the core of GNN development in bioinformatics.

True interpretability in bioinformatics requires alignment between model reasoning and the hierarchical organisation of biological processes. These include transcription factor to gene regulation, domain-specific protein interactions, metabolic pathway flow and clinically relevant disease ontologies. Future architectures should incorporate ontology-aware message passing that respects the hierarchical constraints of Gene Ontology terms, the structural organisation of protein domains and the semantics of clinical phenotypes derived from resources such as HPO. They must also ensure cross-modal consistency for scenarios where variants influence transcriptional output, which subsequently affects protein abundance and interaction profiles. Current models such as FactorGCN and CatGCN point toward this direction, but substantial effort is still required to integrate pathway logic, molecular structure information and uncertainty quantification into the message passing framework. Ultimately, interpretability should enable the extraction of hypotheses that can be validated through experiments such as motif enrichment analysis, targeted gene perturbation or structural docking.

### 5.4. Training Paradigms and Data Scarcity Training Stability, Noise Robustness and Generalisation

Biomedical datasets are characterised by a unique combination of vast unlabelled structures and extremely limited high-quality labels. A single cell atlas contains millions of cells, yet only a very small fraction of regulatory edges are validated. Drug–target interaction networks contain hundreds of thousands of candidate links, yet only a small proportion have biochemical or structural confirmation. Furthermore, clinical outcomes are often inconsistent, sparse and highly heterogeneous due to patient-specific factors.

The training dynamics of GNNs become considerably more complex in bioinformatics because of high measurement noise, severe sparsity and limited labelled samples. These factors amplify over-smoothing, overfitting and instability in message propagation. Therefore, techniques such as graph sparsification, data augmentation and semi-supervised learning are especially important in biological settings.

Current self-supervised learning paradigms provide scalable representation learning but frequently ignore the underlying biological meaning of perturbations and dependencies. Contrasting random subgraphs or disrupting node attributes fails to capture the causal logic of gene regulation, signal transduction or drug response. Biomedicine provides natural and biologically informed pretext tasks. These include predicting phenotypic consequences of gene knockouts, reconstructing developmental trajectories consistent with RNA velocity, simulating perturbation responses in CRISPR datasets, estimating enhancer promoter connectivity from chromatin landscapes and predicting structural compatibility between molecules and protein targets. Such tasks supply mechanistic signals that can inform the embedding space and improve the biological fidelity of learned representations.

Regularisation strategies also require domain awareness. Techniques such as DropEdge or DropNode may inadvertently remove rare but essential genes, for example, TP53 or EGFR, which would disrupt core disease mechanisms. More suitable strategies should incorporate biological centrality measures, pathway significance or experimentally supported essentiality scores. Combining self-supervised learning with domain-specific sampling priors will lead to representations that capture true biological mechanisms rather than generic topological patterns.

These biologically informed training paradigms are particularly valuable because they align model objectives with experimentally observable perturbations, enabling closer coupling between computational inference and empirical validation.

### 5.5. Dynamic and Temporal Graphs

Biological systems evolve continuously through mutation accumulation, cellular differentiation, immune infiltration and therapeutic intervention. Static GNNs are fundamentally unable to represent these dynamic processes because topology and node attributes change simultaneously. Although temporal GNNs can model sequential signals in modalities such as fMRI or electronic health records, most methods assume that the graph structure remains fixed while node embeddings evolve.

To accurately represent biological dynamics, future models must learn both node trajectories and evolving connectivity patterns. Cancer progression provides a clear example where new mutations create regulatory links, clonal expansions reshape pathway activity and targeted therapies selectively remove specific molecular interactions. Developmental biology features time-dependent lineage bifurcations that reorganise transcription factor hierarchies, while immune responses in infection or inflammation involve rapid, dynamic rewiring of cell communication networks unattainable with static edges. Models that integrate continuous time representations or neural ordinary differential equation formulations may better reflect biochemical kinetics and signalling dynamics. When combined with biological constraints, such as the directional nature of signalling cascades and the hierarchical structure of differentiation, dynamic graphs can offer deeper mechanistic insight into disease progression and treatment response.

### 5.6. Scalability, Robustness, and Clinical Evaluation in Bioinformatics

Scalability is paramount in contemporary bioinformatics, as single-cell datasets may comprise tens of millions of nodes, whilst genome-wide variation maps contain billions of edges. Though sample-based graph neural networks enhance computational efficiency, they frequently compromise biological resolution. Simplified aggregation strategies may overlook rare cell populations driving disease or eliminate low-degree nodes representing crucial regulatory factors. Future scalable GNNs must integrate centrality statistics, criticality scores, or prior knowledge of regulatory importance during graph simplification to preserve pathway-level accuracy.

Robustness is equally vital. Biological datasets are susceptible to random gene expression, sequencing losses, labelling errors, batch effects, and population heterogeneity. Moreover, minor perturbations in high-impact genes or clinically relevant laboratory variables may substantially alter downstream interpretative outcomes. Adversarial and noise robustness should therefore be evaluated through biologically meaningful invariants, such as chemical validity in molecular graphs, evolutionary conservation in variation graphs, or pathway consistency in gene regulatory networks.

Beyond accuracy metrics such as node classification or link prediction AUC, clinically aligned evaluation criteria are urgently needed. Models deployed in healthcare settings must prioritise calibration, uncertainty quantification, and counterfactual robustness. A system predicting “high sepsis risk” with 95% confidence is clinically unusable if it cannot explain the underlying cause or suggest actionable interventions. Hence, future evaluations should include actionability scores, intervention sensitivity, and consistency with known drug mechanisms. Likewise, adversarial robustness is critical: GNNs should resist both deliberate and stochastic perturbations by enforcing domain-specific invariants, such as chemical validity constraints for molecules or evolutionary conservation for genomic variants.

### 5.7. Future Directions: Advancing GNNs for Biological Discovery

Looking forward, the advancement of GNNs in bioinformatics will depend on their ability to address domain-specific limitations, including incomplete biological knowledge, dynamic regulatory mechanisms and the integration of multi-modal omics data. Progress in these directions will not only improve predictive performance but also enhance the biological relevance and translational value of GNN-based models.

The future of GNNs in biomedicine lies in the integration of graph learning with biological priors, experimental evidence and clinical reasoning. Progress will depend on methods that couple computational inference with experimentally testable hypotheses. Predicted regulatory modules should guide CRISPR perturbations to refine graph topology; drug repurposing models should yield testable interactions for validation via structural assays or organoid screening; and patient-specific graphs should integrate multi-omics, spatial architecture, and longitudinal clinical data into a unified framework.

Through such integration, GNNs can evolve from descriptive pattern recognisers into prescriptive engines for biomedical discovery. By aligning computational representations with the mechanistic structure of biological systems and the practical requirements of clinical applications, graph neural networks are poised to provide insights that can reshape disease modelling, therapeutic design and precision medicine.

## 6. Conclusions

Graph neural networks have rapidly emerged as a powerful computational paradigm for modelling the relational structure inherent to biological systems. Whether it be molecular maps, protein interaction networks, gene regulatory circuits, or multi-omics association graphs, graph neural networks can effectively preserve the topological structure and semantic features of underlying biological processes while integrating heterogeneous signals. In this survey, we synthesised methodological foundations, architectural developments and application-specific adaptations that have shaped the use of GNNs in bioinformatics, highlighting how design choices in graph construction, model formulation and training strategies jointly determine downstream performance and interpretability.

A central insight emerging from this review is that many of the challenges encountered in applying GNNs to bioinformatics are not merely technical limitations but reflect fundamental properties of biological knowledge itself. Biological graphs are not complete or objective representations of an underlying ground truth; rather, they are epistemic constructs assembled from partial, noisy, context-dependent, and sometimes conflicting experimental evidence. Consequently, uncertainty, ambiguity, and approximation are intrinsic to biological network modelling. These characteristics distinguish biological graphs from many benchmark datasets and impose inherent constraints on what can be learned, generalised, and interpreted from graph-based models.

From this perspective, no single model architecture, optimisation strategy, or dataset can fully capture the complexity of living systems across molecular, cellular, and clinical scales. Effective biological graph learning therefore requires a diversity of complementary approaches, each addressing different aspects of biological organisation and data uncertainty. As discussed throughout this review, progress depends on the integration of biologically informed graph construction, architecture designs that reflect interaction specificity, spatial and hierarchical structure, probabilistic treatments of uncertainty, robust and scalable optimisation strategies, and evaluation protocols aligned with biological and clinical decision-making. Rather than seeking a universal solution, GNN research in bioinformatics must embrace methodological plurality guided by explicit biological assumptions and use-case-specific constraints.

Importantly, advancing GNNs for biological discovery also requires close coupling between computational inference and experimental validation. Predictions derived from graph-based models should be interpreted as hypotheses that guide targeted experiments, such as CRISPR perturbations, structural assays, or functional screening, which in turn refine graph representations and modelling assumptions. This iterative feedback loop is essential for transforming GNNs from descriptive pattern recognition tools into engines for mechanistic insight and causal reasoning.

Looking forward, promising directions include biologically grounded self-supervised and foundation-model approaches for graphs, causal and mechanistic GNN formulations, dynamic and spatiotemporal modelling of regulatory processes, and integrative frameworks that unify sequence, structure, expression, spatial organisation, and phenotype data. Equally critical is the development of standardised benchmarks, transparent evaluation criteria, and reproducible datasets that reflect real biological complexity rather than idealised graph assumptions.

In summary, graph neural networks represent a transformative step toward modelling biological complexity by enabling relational, multi-scale, and system-level reasoning. When developed with explicit awareness of biological constraints and epistemic uncertainty, and when tightly integrated with experimental and clinical practice, GNNs hold substantial potential to reshape disease modelling, therapeutic discovery, and precision medicine.

## Figures and Tables

**Table 1:** Representative Benchmark Datasets for Graph Learning in Bioinformatics.

Dataset	Task Type	Graph Type	Data Source	Version/Access Time
QM9	Molecular property prediction	Molecular graph	GDB-17	Original release
ZINC (subset)	Molecular property prediction	Molecular graph	ZINC database	Filtered benchmark subset
MUTAG	Molecular classification	Molecular graph	NCI	Fixed benchmark
PTC	Molecular classification	Molecular graph	NCI	Fixed benchmark
D&D	Protein classification	Protein structure graph	PDB-derived	Fixed benchmark
PROTEIN	Protein function prediction	Protein graph	Curated dataset	Fixed benchmark
MoleculeNet (e.g., Tox21, HIV)	Multiple molecular tasks	Molecular graph	Multiple sources	As defined in Wu et al.

**Table 2:** Major Biological Databases and Knowledgebases Used for Dataset Construction.

Resource	Resource Type	Data Provided	Typical Use in Graph Learning
ChEMBL	Chemical database	Bioactive compounds, targets, assays	Drug–target interaction datasets
STRING	PPI knowledgebase	Protein–protein interactions	PPI graph construction
KEGG	Pathway database	Metabolic & signalling pathways	Pathway-informed graphs
HPRD	Protein interaction database	Curated PPIs	Tissue-specific networks
GeneMANIA	Functional association resource	Gene–gene associations	Gene prioritisation

**Table 3:** Dataset statistics commonly reported in graph learning benchmarks.

Datasets	Category	Key Characteristics	Graphs	Nodes	Edges	Features	Classes
PPI	Systems Biology	Physical PPIs; tissue context; multilayer networks	24	56,944	818,716	50	121
D&D	Drug-like Molecule	Protein graphs; secondary structure; ligand context	1178	284.31	715.65	82	2
PROTEIN	Drug-like Molecule	Sequence–structure features; functional classes	1113	39.06	72.81	4	2
QM9	Quantum/Property Prediction	Small molecules; quantum properties; structure–property	133,885	-	-	-	-
ZINC	Quantum/Property Prediction	Drug-like compounds; chemical diversity; virtual screening	12,000	23	49	28	-
MoleculeNet	Drug-like Molecule	Standard benchmarks; multiple molecular tasks	7831	-	-	1024	12
ChEMBL	Drug-like Molecule	Bioactivity data; compound–target interactions	100,000	-	-	-	-
NCI-I	Bio-Chemical Graph	Anticancer screening; structural features	4110	29.87	32.3	37	2
PTC	Bio-Chemical Graph	Toxicity labels; chemical structure	344	25.5	-	19	2
MUTAG	Bio-Chemical Graph	Nitroaromatic compounds; structure–activity	188	18	20	7	2

**Table 4:** The comparison between spectral and spatial GCNs.

	Spatial GCNs	Spectral GCNs
Efficiency	Handles large graphs, uses partial nodes, and employs node sampling.	Increases with graph size, involving feature vector computation or processing the entire graph.
Flexibility	Works with diverse image types.	Designed for undirected graphs; converts directed graphs to undirected.
Universality	Supports weight sharing across positions and structures with local node convolution.	Assumes a static graph, making the addition of new nodes challenging.

**Table 5:** Comparison of Bernoulli Sampling and Random-Walk Sampling.

Item	Bernoulli Sampling (B-Dropping)	Random-Walk Sampling (RW-Dropping)
**How nodes are sampled**	Keep each node independently with probability p; drop the rest. Edges remain only if both endpoints are kept.	Run a random walk, ignoring revisits and “jumping” to an unvisited node when stuck, until a target number of distinct nodes is reached.
**Expected subgraph size**	Nodes: n=⌊pN⌋. Edges: m=M(n2)(N2). Produces ER-like subgraphs.	Deterministic node count per sample; pathwise selection via walks. Slower due to the step-by-step procedure.
**Structure preservation**	Structure-agnostic; weakly preserves local clusters; low clustering coefficient.	Statistically preserves local topology; higher C; subgraphs tend toward small-world (σ > 1).
**When to prefer**	Very large graphs or when speed is critical, when strong stochastic regularisation is desired.	When preserving local topology matters (e.g., node classification on graphs with strong clustering), you can afford extra sampling cost.

**Table 6:** Characteristics of Different Graph Data Perturbation and Optimisation Strategies.

Attribute/Method	Dropout	DropNode	DropEdge	Graph Structure Optimisation
**Dropped object**	Feature dimensions	Nodes (and incident edges)	Edges	Edges/Node
**Operates on**	Features inside layers	Subgraph/sampler	Adjacency during training	Preprocessing of the input graph
**Adjacency modified?**	No	Yes (via sampled subgraph)	Yes	Yes (fixed)
**Random each epoch**	Yes	Yes	Yes	No
**Mitigates over-smoothing**	No	Yes	Yes	Yes
**Keeps all nodes**	Yes	No	Yes	Sometimes yes
**Typical cost**	Efficient	Medium	Efficient	Often Costly

**Table 7:** Comparative Analysis of Graph Self-Supervised Learning Methods.

Aspect	Generation-Based	Auxiliary-Based	Contrast-Based	Hybrid
**Core idea**	Reconstruct graph signals (features/adjacency) from perturbed inputs.	Predict handcrafted graph properties (e.g., clusters, pairwise relations) as pseudo-labels or regression targets.	Maximise agreement (often via MI) between positive views and minimise for negatives across augmented graph views.	Combine two or more pretext paradigms to leverage complementary supervision signals.
**Typical pretext**	Feature/edge/adjacency reconstruction loss between the original and reconstructed graph.	Classification or regression on auxiliary properties (clustering labels, pair distances/similarities, centrality, etc.).	Contrastive objectives (e.g., MI estimation) across node/subgraph/graph-level views with positives vs. negatives.	Joint objectives (e.g., reconstruction + contrastive; property tasks + contrastive), multi-task pretraining.
**Data requirements**	Single graph with perturbations to features/structure.	Same graph plus a mapping to properties (clustering, partitioning, distances, similarities).	Multiple augmented views of the graph at chosen granularity; careful augmentation design.	Inputs required by each chosen component; often multiple views plus property mappings/reconstruction.
**Need negatives?**	No.	No.	Often yes (negatives or debiased variants); some methods reduce explicit negatives but still contrast views.	Depends on included components (may inherit contrastive negatives).
**Augmentations**	Light perturbations: feature masking/noise, edge dropout, subgraph sampling.	Not required beyond what the property extractor uses (e.g., clustering/partitioning).	Crucial and non-trivial on graphs (feature masking/shuffling, edge edits, subgraphing, diffusion, etc.).	Often combines augmentations with property/reconstruction pipelines.
**Training scheme compatibility**	Pretrain, finetune or joint with downstream loss as regularisation.	Pretrain, finetune or joint training with the downstream decoder.	Pretrain contrastively finetune, variants also use joint training.	Multi-task pretraining, finetune, or alternating/weighted joint training.
**Example**	GAE, VGAE, superGAT	SimP-GCN	GRACE, MVGRL, GCC, MICRO-Graph	LnL-GNN, MVMI-FT, GPT-GNN
**Function**	$\theta^{*},\phi^{*}=arg{min}_{\theta ,\phi }L_{ssl}$ $\left(p_{\phi }(f_{\theta }(\overset{\sim }{G})),G\right)$	$\theta^{*},\phi^{*}=arg{min}_{\theta ,\phi }L_{ssl}$ $\left(p_{\phi }(f_{\theta }(G)),c\right)$	$\theta^{*},\phi^{*}=arg{min}_{\theta ,\phi }L_{ssl}$ $\left(P_{\phi }(f_{\theta }({\bar{G}}^{\left(1\right)}),f_{\theta }({\bar{G}}^{\left(2\right)}))\right)$	$\theta^{*},\phi^{*}=arg{min}_{\theta ,\phi }$ $\sum_{i=1}^{N}\alpha_{i}L_{ssl_{i}}(f_{\theta },p_{\phi_{i}},D_{i})$

**Table 8:** Performance Comparison of Predictive Models for Disease Association Prediction.

Methods	Category	Training Datasets	Metrics	Headline Result
DimiG [135]	miRNA–disease Association	GTEx (Genotype-Tissue Expression Project)	AUC	0.748
			PRC	0.765
CNC [135]	miRNA–disease Association	GTEx (Genotype-Tissue Expression Project)	AUC	0.518
			PRC	0.599
miRBD-i-max [136]	miRNA–disease Association	GTEx (Genotype-Tissue Expression Project)	AUC	0.716
			PRC	0.434
VGAE-MDA [127]	miRNA–disease Association	HMDD v2.0 (Human MicroRNA Disease Database, version 2.0)	AUC	0.9394
			PRC	0.939
DBN-MF [137]	miRNA–disease Association	HMDD v2.0 (Human MicroRNA Disease Database, version 2.0)	AUC	0.9169
			PRC	0.9043
GCAEMDA [128]	miRNA–disease Association	HMDD v3.2	AUC (5-fold CV)	0.9415
			AUC (global LOOCV)	0.9505
NIMCGCN[138]	miRNA–disease Association	HMDD v3.2	AUC (5-fold CV)	0.9378
			AUC (global LOOCV)	0.941
MDA-GCNFTG [129]	miRNA–disease Association	HMDD v2.0 (Tp-Balanced)	AUC (5-fold CV)	0.9973
			PRC (5-fold CV)	0.9977
GATMDA [130]	miRNA–Target Association	miRTarBase+ TarBase v8+ miRecords	AUC	0.93684
			PRC	0.95327
NeoDTI [139]	miRNA–disease Association	HMDD v2.0 (Tp-Balanced)	AUC	0.8301
			PRC	0.8568
MSGCL [140]	miRNA–disease Association	HMDD v2.0 (Tp-Balanced)	AUC	0.837
			PRC	0.9162
AMHMDA [141]	miRNA–disease Association	HMDD v2.0 (Tp-Balanced)	AUC	0.9176
			PRC	0.9167
MGCNSS [142]	miRNA–disease Association	HMDD v2.0 (Tp-Balanced)	AUC	0.9874
			PRC	0.9882
		HMDD v2.0 (positive:negative = 1:5)	AUC	0.9586
			PRC	0.9758
ReHoGCNES-MDA [131]	miRNA–disease Association	HMDD v2.0 (Tp-Balanced)	AUC	0.9998
			F1-Score	0.9957
Node2Vec [143]	disease–gene association	Stanford Biomedical Network Dataset Collection	AUC	0.86
			AP	0.846
DGP(C-VGAE) [132]	disease–gene association	Stanford Biomedical Network Dataset Collection	AUC	0.908
			AP	0.913
PDGCN [133]	personalised cancer driver gene prediction	TCGA (The Cancer Genome Atlas)	AUC	0.928
			PRC	0.813
LIMO-GCN [134]	disease–gene association	AD GWAS summary statistics	AUC	0.943
			PRC	0.728

**Table 9:** Performance Comparison of Predictive Models for Drug Development.

Methods	Category	Training Datasets	Metrics	Headline Result
Graph-CNN [144]	Drug–Target Interactions	DUD-E	AUC	0.886
3D-CNN [154]	Drug–Target Interactions	DUD-E	AUC	0.868
tCNNs [155]	Drug–Target Interactions	GDSC v6.0 (mixed test)	RMSE	0.0244
			CCp	0.916
GraphDRP-GCN [145]	Drug–Target Interactions	GDSC v6.0 (mixed test)	RMSE	0.0259
			CCp	0.9216
GraphDRP-GIN [145]	Drug–Target Interactions	GDSC v6.0 (mixed test)	RMSE	0.0244
			CCp	0.931
GraphDRP-GAT [145]	Drug–Target Interactions	GDSC v6.0 (mixed test)	RMSE	0.025
			CCp	0.927
GraphDRP-GCN_GAT [145]	Drug–Target Interactions	GDSC v6.0 (mixed test)	RMSE	0.0243
			CCp	0.9308
GLCN-DTA [146]	Drug–Target Interactions	Davis (Kd)	MSE	0.0215
			Cl	0.903
			r2m	0.72
Decagon [147]	Drug–Drug Interactions	TWOSIDES	AUC	0.872
			PRC	0.832
RESCAL [156]	Drug–Drug Interactions	TWOSIDES	AUC	0.693
			PRC	0.613
DeepWalk [157]	Drug–Drug Interactions	TWOSIDES	AUC	0.761
			PRC	0.737
M2GCN [148]	Drug–Drug Interactions	SIDER + OFFSIDES + TWOSIDES +PPI + Drug–Target	AUC	0.9483
			PRC	0.9531
SLiCE [148]	Drug–Drug Interactions	SIDER + OFFSIDES + TWOSIDES +PPI + Drug–Target	AUC	0.84
			PRC	0.8528
DDIPred [149]	Drug–Drug Interactions	BIOSNAP DDI network	AUC	0.996
			PRC	0.967
DDI-GCN [150]	Drug–Drug Interactions	BIOSNAP DDI network	AUC	0.999
			PRC	0.99
DDI-OCF [151]	Drug–Drug Interactions	DrugBank v5.1.9 + TWOSIDES	AUC	0.881
			PRC	0.624
Ada-DR [152]	Drug Repositioning	Gdataset + Cdataset + LRSSL + Ldataset	AUC	0.937
			PRC	0.576
iDrug [158]	Drug Repositioning	Gdataset + Cdataset + LRSSL + Ldataset	AUC	0.892
			PRC	0.143
BNNR [159]	Drug Repositioning	Gdataset + Cdataset + LRSSL + Ldataset	AUC	0.919
			PRC	0.282
DRHGCN [159]	Drug Repositioning	Gdataset + Cdataset + LRSSL + Ldataset	AUC	0.931
			PRC	0.488
DRWBNCF [160]	Drug Repositioning	Gdataset + Cdataset + LRSSL + Ldataset	AUC	0.906
			PRC	0.453
DRSPRING [153]	Drug combination	O’Nell Dataset (Leave Combinations out)	MSE	100.5704
			PCC	0.8193
DeepSynergy [161]	Drug combination	O’Nell Dataset (Leave Combinations out)	MSE	96.9472
			PCC	0.8041

**Table 10:** Performance Comparison of Predictive Models for Protein Analysis.

Methods	Category	Training Datasets	Metrics	Headline Result
GCN (Node&Edge Average) [162]	Protein–protein interface prediction	Docking Benchmark Dataset v5 (DBD 5)	AUC	0.898
DCNN [59]	Protein–protein interface prediction	Docking Benchmark Dataset v5 (DBD 5)	AUC	0.828
DTNN [162]	Protein–protein interface prediction	Docking Benchmark Dataset v5 (DBD 5)	AUC	0.882
GraphQA [163]	Protein–protein interface prediction	CASP13 stage-2	RMSE	0.13
			R	0.855
			R-Target	0.779
ModFOLD7_rank [172]	Protein–protein interface prediction	CASP13 stage-2	RMSE	0.156
			R	0.872
			R-Target	0.742
ProQ3D [173]	Protein–protein interface prediction	CASP13 stage-2	RMSE	0.146
			R	0.802
			R-Target	0.637
Pinet [164]	Protein–protein interface prediction	DBD5	AUC	0.877
			PRC	0.734
BIPSPI [174]	Protein–protein interface prediction	DBD5	AUC	0.827
			PRC	0.429
GAT + LSTM-based LM [164]	Protein–protein interface prediction	Human & *S. cerevisiae*	AUC	0.9838 (Human), 0.9724 (*S. cerevisae*)
			PRC	0.9896 (Human), 0.9650 (*S. cerevisae*)
DeepFRI [166]	Protein function prediction	PDB700	Fmax	0.657
DeepGO [175]	Protein function prediction	PDB700	Fmax	0.525
DeepGraphGO [167]	Protein function prediction	CAFA4	Fmax	0.623
			AUPR	0.543
NetKNN [167]	Protein function prediction	CAFA4	Fmax	0.426
			AUPR	0.276
PANDA [176]	Protein function prediction	CAFA3	Fmax	0.486
			Smin	11.751
			AUPR	0.396
PANDA2 [168]	Protein function prediction	CAFA3	Fmax	0.598
			Smin	9.67
			AUPR	0.964
DeepGOPLUS [177]	Protein function prediction	CAFA3	Fmax	0.585
			Smin	8.824
			AUPR	0.536
GAT-GO [169]	Protein function prediction	Human proteins with AlphaFold2 structures	Fmax	0.633
			AUC	0.912
			AUPR	0.776
Struct2GO [169]	Protein function prediction	Human proteins with AlphaFold2 structures	Fmax	0.701
			AUC	0.969
			AUPR	0.796
PhiGNet [106]	Protein function prediction	CAFA3	Fmax	0.606
			AUPR	0.571

## Data Availability

No new data were created or analyzed in this study.
